# Support Vector Machine Classification of Streptavidin-Binding Aptamers

**DOI:** 10.1371/journal.pone.0099964

**Published:** 2014-06-13

**Authors:** Xinliang Yu, Yixiong Yu, Qun Zeng

**Affiliations:** 1 College of Chemistry and Chemical Engineering, Hunan Institute of Engineering, Xiangtan, Hunan, China; 2 State Key Laboratory of Chemo/Biosensing and Chemometrics, College of Chemistry and Chemical Engineering, Hunan University, Changsha, Hunan, China; 3 G1302, Lushan International Experimental School, Changsha, Hunan, China; 4 Department of Neurological Surgery, Xiangtan Central Hospital, Xiangtan, Hunan, China; Imperial College London, United Kingdom

## Abstract

**Background:**

Synthesizing and characterizing aptamers with high affinity and specificity have been extensively carried out for analytical and biomedical applications. Few publications can be found that describe structure–activity relationships (SARs) of candidate aptamer sequences.

**Methodology:**

This paper reports pattern recognition with support vector machine (SVM) classification techniques for the identification of streptavidin-binding aptamers as “low” or “high” affinity aptamers. The SVM parameters *C* and *γ* were optimized using genetic algorithms. Four descriptors, the topological descriptor *PW4* (path/walk 4 - Randic shape index), the connectivity index *X3A* (average connectivity index chi-3), the topological charge index *JGI2* (mean topological charge index of order 2), and the free energy *E* of the secondary structure, were used to describe the structures of candidate aptamer sequences from SELEX selection (Schütze *et al.* (2011) PLoS ONE (12):e29604).

**Conclusions:**

The predicted fractions of winning streptavidin-binding aptamers for ten rounds of SELEX conform to the aptamer evolutionary principles of SELEX-based screening. The feasibility of applying pattern recognition based on SVM and genetic algorithms for streptavidin-binding aptamers has been demonstrated.

## Introduction

Aptamers are structured single-stranded oligonucleotides that show an affinity toward a variety of targets, including proteins, viruses, and whole cells [1], [2]. Compared to antibodies, aptamers are economical and easy to synthesize and modify, possess long-term stability, and display low immunogenicity, fast blood clearance, rapid tissue and tumor penetration. Furthermore, unlike antibodies developed *in vivo*, aptamers can be developed *in vitro* [3]–[5]. Aptamers, a promising class of compounds, both for target recognition and therapy, can be derived from a process termed Systematic Evolution of Ligands by EXponential enrichment (SELEX). This is a reiterative process of partitioning of aptamer candidates from non-binding sequences by an affinity method, followed with amplification of the bound sequences by polymerase chain reaction (PCR) [5].

The conventional SELEX method usually takes 10–15 cycles of selection and amplification, which are labor-intensive and time-consuming [6]. Furthermore, the consumption of samples/reagents is relatively high. By applying the relationship between the chemical structure of a molecule and its biological activity or properties, i.e., the structure–activity relationship (SAR) model, pattern recognition can be used to screen a series of candidate molecules, including those not yet synthesized, on the computer in order to select the structures having the desired set of predicted activities/properties [7]. It is then possible to select the most promising candidates for synthesis, laboratory testing, and optimization. Thus, the pattern recognition approach based on such SARs as classification models can conserve resources and accelerates the process of selecting candidates for any purpose.

Streptavidin is widely used as a detection tool in biology research because of the strongest non-covalent interaction known in nature between streptavidin and biotin [8], [9]. In addition, biotin-streptavidin system can be explored for detection of infection and tumor in clinical medicine [10]. Support vector machines (SVMs) are a popular technique for classification. The aim of this paper is to develop a pattern recognition model for aptamers against streptavidin, using SVM as the classification technique. A genetic algorithm is employed to find suitable parameters for SVM model that has relatively optimal prediction performance.

## Materials and Methods

### Data set

Table S1 shows the candidate aptamer sequences, which were taken from Reference [9]. These candidate aptamers were obtained through ten rounds of SELEX selection and the 100 most frequent clones of each round were listed. Each of the clones contains 40 bases with a tolerance of two bases. The candidate aptamers were split into two sets: a training set and a test set. The training set includes the sequences from the 1st and 10th rounds of SELEX selection. The sequence R10#86 in the 10th round recurs in the 1st selection round, i.e., R1#1. In addition, the sequences R1#11 and R1#60 have no pair structure. Therefore, the three sequence, R10#86, R1#11 and R1#60 are removed from the data set of the 1st round selection. Totally, 197 different sequences were obtained to generate the training set. The test set contains the sequences from the rest of SELEX rounds, i.e., the 2nd, 3rd, 4th, 5th, 6th, 7th, 8th, and 9th rounds. Similarly, the sequences R2#55, R2#63 and R2#94 without pair structure are deleted from the test set.

In general, the binding affinity of aptamers with targets increases exponentially with increasing selection rounds. Such exponential increase in binding affinity is obvious during the first few rounds. In the end, the affinity is close to a saturation point after a certain number of rounds are carried out, and subsequently the binding affinity does not increase obviously [11]–[13]. Thus, the class labels (or target values) of sequences from the 1st round of SELEX were set as 1, denoting the low affinity and specificity aptamer candidates. The class labels of sequences in the 10th round were defined as 2, denoting the high affinity and specificity aptamer candidates. The training set was used to train and optimize algorithm parameters of SVM models. The test set was used later to evaluate predictive performance of the developed model.

### Molecular descriptor calculation

The RNAstructure package (version 5.3) was used for prediction of secondary structures of candidate aptamers by minimizing the free energy (*E*) [14]. The loop structures were adopted to calculate molecular descriptors for corresponding candidate aptamers. Since the size of the loop is important for binding, the priority was given to the loops with 5–7 nucleotides, which were selected to calculate descriptors. Besides the free energy descriptor (*E*), three groups of molecular descriptors were calculated with Dragon software [15], which are 119 topological descriptors, 21 topological charge indices, and 33 connectivity indices. Totally, 174 molecular descriptors were calculated for each sequence. To calculate molecular descriptors, the loop of each aptamer candidate was sketched using ChemBioDraw Ultra 11.0 [16], and optimized using molecular mechanics (MM2) in ChemBio3D Ultra 11.0 until the *rms* of gradient value became smaller than 0.1 kcal/mol Å. The energy minimized molecules were then used as the inputs for Dragon software [15].

Topological descriptors based on a graph representation of a molecule can describe one or more such chemically interesting features as size, shape, symmetry, branching and cyclicity. They can also reflect chemical information like atom type and bonding environments [15]. Connectivity indices are calculated from the non- hydrogen part of a molecule where each vertex (non-hydrogen atom) is weighted by the vertex degree, i.e., the number of connected non-hydrogen atoms [17]. Topological charge descriptors are derived from an unsymmetrical matrix CT, whose single element *CT*_ij_ is equal to the vertex degree *δ*_i_ of the *i*th atom under the condition of *i*  =  *j*. Otherwise, *CT*_ij_ equals to the difference of *m*_ij_ and *m*_ji_. Here *m*_ij_ and *m*_ji_ are elements of the matrix obtained by multiplying the adjacency matrix by the reciprocal square distance matrix [15], [18]. For each path of length *k*, a topological charge index *GGIk* is defined as the half-sum of all charge terms *CT*_ij_ (absolute values) corresponding to pair of vertices with topological distance equal to *k* [15], [18].

### Principle of support vector machine classification

SVMs are known as maximum-margin classifiers, since they find the optimal hyperplane that is equidistant from two classes and defined by a number of support vectors. In general, the larger the margin or distance between these parallel hyperplanes, the smaller the generalization error of the classifier will be [19]–[22].

Let (**x**_i_; *y*_i_) be a set of training examples, where *i*  =  1, 2 …, *n*, **x** *_i_* ∈ R*^d^* is an input vector, *y_i_* ∈ {−1, 1} is its corresponding desired output, i.e., a constant denoting the class to which that point **x** *_i_* belongs, *n* is the number of training data, and *d* denotes the number of dimensions of input data.

The SVM requires the solution of the following optimization problem
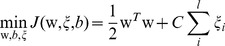
(1)subject to:
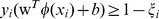
(2)

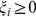
(3)


Here **w** is the weight vector, *b* is the bias, *ξ* is a non–negative slack variable for the data points, and *C* is a penalty factor that controls the tradeoff between the complexity of the decision function and the number of training examples misclassified. SVM maps input vectors **x** *_i_* into a higher (may be infinite) dimensional space, where a margin hyperplane with the maximal margin is constructed. Under constraints 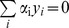 and 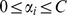, the optimization problem becomes
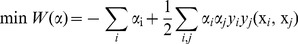
(4)


Quadratic programming method can be adopted to solve the above extreme problem. The points **x** *_i_* with *α*_i_>0 are called support vectors. Patterns with 0<*α*_i_ < *C* are called unbounded support vectors, while those with *α*_i_  =  *C* are called bounded support vectors [22]. SVM can be easily generalized to non–linear decision surfaces by replacing the inner product (x*_i_*•x*_j_*) with a kernel function *K*(x*_i_*,x*_j_*). The Gaussian radial basis function kernel (RBF) is a popular kernel function used in SVM classification and can be expressed with
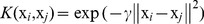
(5)


Here *γ* is a kernel parameter. For the SVM classification models based on the RBF kernel, two parameters, *C* and *γ*, should be carefully tuned to the problem at hand. If the factor *C* is too large, a large penalty is assigned to non-separable points, which leads to store too many support vectors and thus over fit. On the other hand, if *C* is too small, an under fitting can occur. The *γ* parameter specifies the radius of the RBF, also exerting a strong impact on model performance [23].

In this paper, we used the genetic algorithm to optimize the SVM parameters, *C* and *γ*. Genetic algorithm belongs to the family of evolutionary algorithms, which generate solutions to optimization problems using techniques inspired by the evolution of species emphasizing the law of survival of the strongest [24]. It uses random mutation, crossover and selection procedures to breed better models or solutions from an originally random starting population or sample [25]

The parameter values used in our experiments were set as following: the population size of genetic algorithm being 20, evolutionary generation being 200, and both the SVM parameter, *C* and *γ*, being selected in the ranges [1, 1000]. The 5-fold cross validation procedure was carried out for the training set during the optimization of SVM parameters, *C* and *γ*. LibSVM package [26] was used to develop the SVM models.

## Results and Discussion

The selection of appropriate theoretical descriptors is crucial to obtain good classification performance. With the number of selection rounds being the dependant variable and 174 molecular descriptors being the independent variables, the correlation between independent and dependent variables was analyzed with stepwise multiple linear regression (MLR) in SPSS 11.5, to select an optimal subset of variables used as the inputs of the SVM model. Four descriptors, the topological charge index *JGI2* (mean topological charge index of order 2), the topological descriptor *PW4* (path/walk 4 - Randic shape index), the connectivity index *X3A* (average connectivity index chi-3), and the free energy (*E*) of the secondary structure were obtained to describe the structure features of each candidate aptamer, which are listed in Table S2.

Molecular connectivity indices are used widely in various areas of physical, chemistry, biology, pharmacology, polymer, and environmental science. One of the most important reasons about their successful applications is that these indices are based on sound chemical, structural (topologic and geometrical), and mathematical grounds. The descriptor *X3A* (a connectivity index; average connectivity index chi-3) belongs to average connectivity indices *XkA*, which are obtained by dividing each connectivity index by the number of paths involved in its calculation [15]

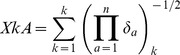
(6)


Where *δ*_a_ represents the corresponding vertex degree, *k* is the total number of *m*th order subgraphs, and *n* is the number of vertices in the subgraph. The descriptor *X3A* (*k* = 3 for *XkA*) relates the characteristic dimension of the molecule to the atomic parameters (quantum number, bond indexes, etc.) [27]. *X3A* can denote the molecular size and the electronic distribution of a loop, which is related to the induced fitting behavior and molecular recognition of an aptamer.

Mean topological charge indices *JGIk* (from order 1 to order 10) are obtained by dividing the corresponding topological charge index (*GGIk*) by the total number of summation terms in *GGIk*. The mean topological charge index *JGI2* (*k* = 2 for *JGIk*) is related to the topological valence of the atoms and the net charge transferred from the atom *j* to the atom *i* [15].

Path/walk Randic shape indices (*PWk*) are calculated by summing the ratios of the atomic path count over the atomic walk count of the same order *k* and then dividing by the number of non-H atoms [15], [28]. DRAGON calculates path/walk shape indices from order 2 up to 5. The index of first order is not provided as the counts of the paths and walks of length one are equal and, therefore, the corresponding molecular index always equals one for all molecules [15]. Since path/walk count ratio is independent of molecular size, the topological descriptor *PW4* (path/walk 4 - Randic shape index) can be considered as a shape descriptor.

**Figure 1:**
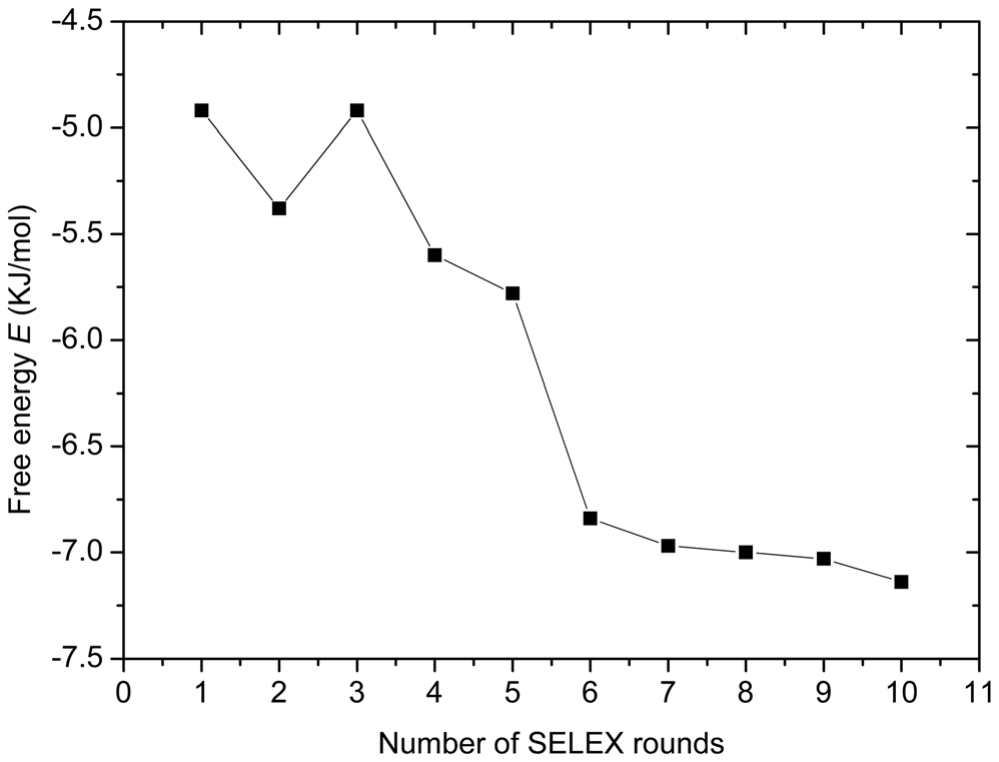
Correlation of the mean free energy and the number of performed rounds.

The free energy *E* of an aptamer secondary structure is approximated as the sum of individual contributions from loops, stacked base pairs, and other secondary structure elements. Aptamer molecules fold by intramolecular base pairs and are stabilized by hydrogen bonds that form between the base pairs along the DNA or RNA molecule. In addition, base pair stacking in a helix also stabilizes the molecule and decreases the free energy of the folded aptamer. Thus the free energy (*E*) of the secondary structure reflects the conformational stability of an aptamer. Generally, an aptamer with a high free energy *E* (absolute value) does not mean a stronger binding with its targets. Because the binding is an aptamer-target interaction provided by different intermolecular interactions such as electrostatic interactions between charged groups, stacking of aromatic structures contained in organic compounds and the nucleobases, hydrogen bonds, and the complementary in three-dimensional shape [29], while the free energy here is the property of an aptamer only. For streptavidin-binding aptamers, a longer stem of an aptamer secondary structure leads to a larger descriptor *E* (absolute value). On the other hand, a longer stem can more effectively maintain the loop and bulge structures for binding [8]. Therefore, for a streptavidin DNA aptamer, its free energy *E* is correlated with its binding ability to streptavidin. Figure 1 shows the correlation of the mean free energy (*E*) of the secondary structure and the number of rounds of SELEX [9]. As can be seen, the descriptor *E* decreases with the increasing number of performed rounds.

Selecting appropriate values for parameters, *C* and *γ*, is also important for SVM performance. The optimization results show that the relatively optimal SVM model possesses parameters *C* of 705.933 and *γ* of 749.802. The model based on *C* = 705.933 and *γ* = 749.802 has classification accuracy of 97.98% for the training set. To evaluate the model, we calculated prediction values for the test set, which are listed in Table S2. In Reference [9], at identical concentration (1 mM), the binding affinity of eight sequences of R10#1, R10#2, R10#4, R10#6, R10#10, R10#17, R10#62, and R10#86, were studied. Only the sequence R10#62 has low prediction class labels being 1, which is acceptable since R10#62 really has a weak binding ability to streptavidin [9].

**Figure 2:**
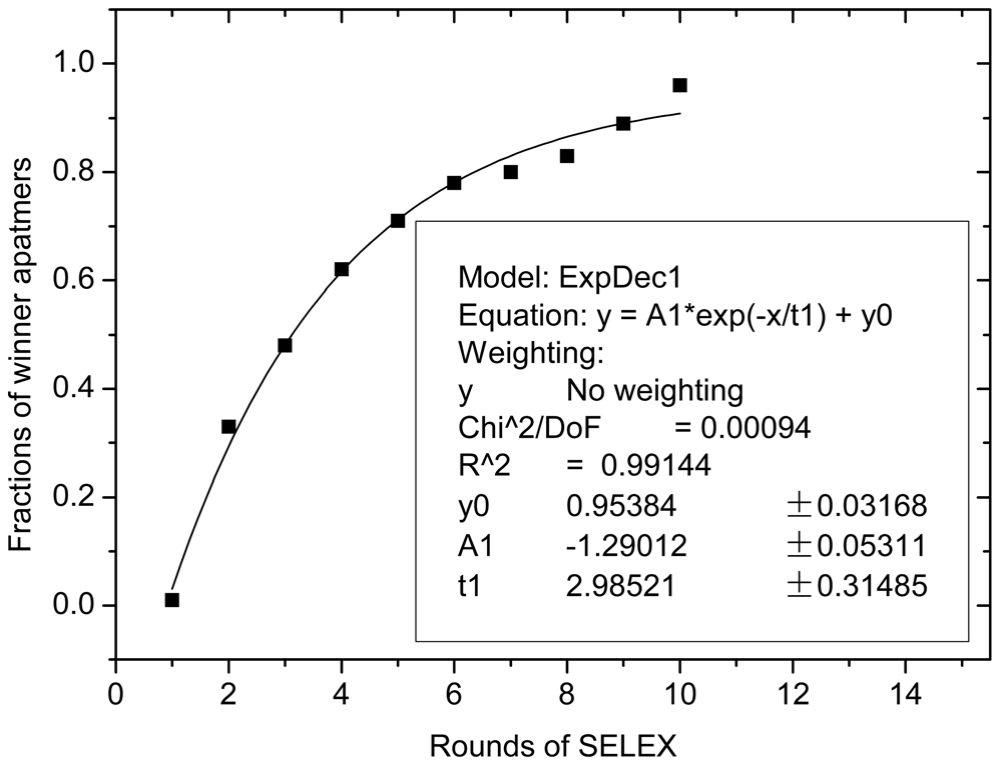
Evolutionary curve of streptavidin-binding aptamers.

For *in vitro* DNA aptamer selection procedures based on SELEX technology, starting point of each SELEX process is a synthetic random DNA oligonucleotide library consisting of a multitude of single-stranded DNA fragments (approximately 10^15^) with different sequences. After the library and the target molecules are incubated for binding, unbound oligonucleotides are removed by several stringent washing steps of the binding complexes. The target-bound oligonucleotides being eluted and subsequently amplified by PCR are then used to generate a new enriched pool for the next selection round. During the SELEX process, the average binding affinity of the selected sequence against specific targets can increase exponentially with the number of selection rounds, just as the word “exponential” suggests in the term of Systematic Evolution of Ligands by EXponential enrichment (SELEX) [11]–[13]. The fractions of winning aptamers (i.e. their prediction class labels being 2) from the first round to 10th rounds of experiments are 0.01, 0.33, 0.48, 0.62, 0.71, 0.78, 0.80, 0.83, 0.89, and 0.96, respectively. We can obtain the following fitting curve and the corresponding exponential equation in Figure 2, which shows the average binding affinity of the candidate sequences towards streptavidin increases exponentially during the first six rounds of SELEX (the experimental results also show that the binding signal increased strongly until round six [9]). After that, the affinity is close to the saturation point, the fractions of winning aptamers being 08, although the binding affinity increases gently in subsequent rounds. Obviously, the prediction result consists with the aptamer evolutionary principles of SELEX based screening, as stated above [11]–[13].

## Conclusions

The four descriptors, *PW4*, *X3A*, *JGI2*, and *E*, reflecting the structures of candidate aptamer sequences, were used as the input variables of SVM model. Genetic algorithms were chosen to optimize the SVM parameters, *C* and *γ*. The relatively optimal SVM model with parameters *C* of 705.933 and *γ* of 749.802 has classification accuracy of 97.98% for the training set. Furthermore, the prediction fractions of winning aptamers from the 1st round to 10th round are 0.01, 0.33, 0.48, 0.62, 0.71, 0.78, 0.80, 0.83, 0.89, and 0.96, respectively. The prediction result consists with the aptamer evolutionary principles of SELEX based screening, which shows that pattern recognition for streptavidin-binding aptamers is successful. The investigation may encourage the application of pattern recognition methods to the designs of candidate aptamers.

## Supporting Information

Table S1 **List of 995 candidate aptamer sequences for streptavidin containing 40 bases with a tolerance of two bases.**
(DOC)

Table S2 **List of the topological charge index** ***JGI2*** **, topological descriptor** ***PW4*** **, connectivity index** ***X3A*** **, the free energy** ***E*** **, and predicted class labels for 995 candidate aptamer sequences.**
(DOC)
