# Supplementary material for: Support Vector Machine Classification of Streptavidin-Binding Aptamers
Source: PLoS One. 2014 Jun 13;9(6):e99964. doi: 10.1371/journal.pone.0099964 (PMC4057401; doi:10.1371/journal.pone.0099964)
Supplement: Table S1 — List of 995 candidate aptamer sequences for streptavidin containing 40 bases with a tolerance of two bases. (DOC) [file pone.0099964.s001.doc]

**Table S1**  **995 candidate aptamer sequences for streptavidin^a^**

| **No.** | **Sequence** |
| --- | --- |
| R1#1 | ATCTCCGATTGCCCCACGACGCAGTGGTCGGAGATACTTT |
| R1#2 | CAGGTGATTTGCCACGAGAGTCATACAGTCCTTGTGTGGG |
| R1#3 | GACGCCAGGGCGTGTGTGCCTGAGATGTCACCAGGGAGTGC |
| R1#4 | AGTACCTGTCAGAGTAGATTCGCGATGGTATCAAGATGCG |
| R1#5 | ACGGTTATAGGGAGTCTGTCTGGTTTTGAAGTGGTATCAC |
| R1#6 | TGGACCTGTCTCGCGGGCGTCTGAATTGTTATTTTTGCCG |
| R1#7 | GATTCAACGCCGTTTCGGTTTGAACTTAGCCGGGATCCCA |
| R1#8 | CCACGCTTGGGGACATACCATGAATGGGTATGCTGTCCA |
| R1#9 | TGTTTCGCCTTGTTCTTGCGGCCTTCCCTCCGAGGGTGAT |
| R1#10 | GCGCAATCTCTGATCGACCAGGAGGGAGCGAAACATGGTG |
| R1#12 | GGCTGGGTACATCGGAAAGGTACTGTCTTACGCTCAGTCC |
| R1#13 | GAATCCCGCGTGACCAATTACGCTGGATGCCGAGTGCC |
| R1#14 | CATGGCGCAGTTCGTGGGGCCATGGAGTCTCACGCAGTG |
| R1#15 | GGGGGGCAACCCACGGGGACGTTAGACAGCCTCGGCTAA |
| R1#16 | CTGTGCCTGTTTGTCAACATTAGGGATGATTCGGTGACAC |
| R1#17 | TAATAGCGCTTCCGGTCAGGGCCTTAAAACTGACCGCGGA |
| R1#18 | ATATGGACCTTTCCCACCACCCACTGTAATCATTCCCGT |
| R1#19 | AGGCGACACAAGCCGTATGCCCTAACTCGTTACCGTGCCT |
| R1#20 | TAGTACTTCCCGTTTCTGGGTTTGTAACCTTCCCTCCCGT |
| R1#21 | TAGACACCCCGAGTTGCCATTCATTTGCATATGCGTTACT |
| R1#22 | GAGGGTGGCGAGGAAACCGGTTAGTAGGATCTCTTGGATT |
| R1#23 | GGGAGTCGGGTACTTTTCGGTTTGGTCAAGCATAGATGCG |
| R1#24 | CGTTGGTCACTCTGATTTGGTCCGAGGTAGACTTCGGCCC |
| R1#25 | CGGTTTTACTTTTCCTCATGCTTTCGGAAGAACGCCTAAT |
| R1#26 | TTTGGAGGTAAGTTTGGCGCCTTCCAATCATCCCTCCGTG |
| R1#27 | TCTTAGCGGCCTGCGACGTATTATTCACCGCGCTATGGCT |
| R1#28 | CCTCGAAAGGTATATTAGGGAAGGTTTATATGCAATGCAT |
| R1#29 | TGTCCCCTGTCTGTTCACACGCGGTCTCTTGTACGACCCG |
| R1#30 | TTTACCTTGTGGTCCTGATTATGAGTTATTGGTCGGTGTG |
| R1#31 | ACGCTGCCAGATGTTTCCGGGCAGCTTTGTGTCGCCGCGT |
| R1#32 | GGACCCTGGGACTCCTACTTAGGTAGGTTACCCCTTGTCC |
| R1#33 | CATAGCGATGGAATCCAGTCGCATGGAATTGCAAAGGCTC |
| R1#34 | TGTGCGAAGTATGCATAACAGTAATCTGGTCTGTCTTCC |
| R1#35 | GAACGAGGTGCAGATGTTATCTGTTGGCCCAGGGACCACC |
| R1#36 | AGCCCTGGGCTATAATTTGGAGCATCAGAAGACAATTAGG |
| R1#37 | GCATCAAGGCCACCATGTCGATGTTTTTTAATTATTGTTC |
| R1#38 | AGGGTAACCGACCCCGTGACTGCACGCTCCGTTTGGCGCC |
| R1#39 | CGCTCAAAGGGATTCGATGTGTTTAGGACTGTAGTGTACC |
| R1#40 | GGACAGAATGTCGCTTTGTACACGTGGGGTTTGATTCTGT |
| R1#41 | GTGGCATGTGCGGCAAATGACCTTATGCGTTTCGCAGGCT |
| R1#42 | GCATTTCAGGCTCATCAGGACAAAGCTCGCAACTAATCGC |
| R1#43 | CCAGGCATAGTGACCTTCTCAAAATCAGTGAGCACCTGCA |
| R1#44 | CAGGTTCGGCCACGTATTGCCACGCACGTTACACGAATCT |
| R1#45 | CGTGTGTAAAAGTCTGCTGGACTATTCCCGAGATCGTC |
| R1#46 | CAAATATATTTTGATAATACAATTAAAGATCTTAACGGCT |
| R1#47 | GCGGCGCTAATGGACTCTCGAACGATAGTGCTCGTCCC |
| R1#48 | GGCACAGGGACGAGAATCGGACCCTGTGTTCACCGGCCCT |
| R1#49 | AACATAACATAAGGGTAATCTTGTCCTCGCTTATTTAGGT |
| R1#50 | AGGTTCACCAATCGTACATCTTCCTCTTTGCCATGTTGGA |
| R1#51 | TCGTTACCTTGTTGGGGTGGGTGTAACACCCTGGCCCAAA |
| R1#52 | GGTGTACGCCGACTCGTTTCGGGGTTTGCCGGTACATATT |
| R1#53 | GCTTGGCATGTTCGGGGGTCTAGACGCACGTACACCATTG |
| R1#54 | ACACGGGATAGCACATTAGGAGCATTCTATGCTCTTAGCT |
| R1#55 | CATAACGAGTGGTTATGAGATTGTTCTTTCATTTGCTAGT |
| R1#56 | GCCAAACCCACAATAGTTTATATTTTCGATGTACACGGT |
| R1#57 | TGCCACAATTGCGGGTAAAACCACGAATGGAACAGTTC |
| R1#58 | GCTGAACAATCCCACACTGGGCCTTGTTAACAGTGAGGGC |
| R1#59 | GCCCAGAGCTAAAAGAAATGGGTGTTAATCCGATCGCAC |
| R1#61 | TTTACCCGCGATCTGAGGTACGGATTACCGGGCAGTGTGA |
| R1#62 | AGCAGTACCGTAGGTGGTTACGTCACTGAGGTGATACCAG |
| R1#63 | ACCGTCGTCATAAACCGCTTTTTTATCTCCGGCACTGCCC |
| R1#64 | ACGTGCTCTTGAGATACCTCAGTATTCGAGGGTTAGGCT |
| R1#65 | GGGCACTCAACGGTTCTAGGGATTCGAGCAGTAGCTTCAC |
| R1#66 | GGTGAGATAGGGCCGGTGGACTCAAGGTTGTTTCGGTCCA |
| R1#67 | CTAGACCTAGTGCAAAGGCAAGAGCATATGACGCCTTGC |
| R1#68 | GGTACGCGGCTGCGGTAAGCCGTAGCCGTGACTATGCGT |
| R1#69 | TCGGACTATCCCGTTAGTGCGCTCAAGACAGGGTACCACG |
| R1#70 | ACTCAAGTAACAGATTGCTGATAGCAACGTCCGAAGTGCA |
| R1#71 | TCATATCCCCGGTCTCACCAGGGCGGGCCTTGGGATGTGC |
| R1#72 | GGCCATCCTTTTCAGCGCAGGGGTCACTTACGTGACAGGG |
| R1#73 | AGGCCACGAAGTGTCCTGCTTCTATAGAGGAGTATTTTCT |
| R1#74 | GTGGCGCACCGGAACTTTTGTCGAGGGTGCTTGTAGGTC |
| R1#75 | CACCGGGTCGGCCCTTGTTTGTCACGCCCTGGAGATATCC |
| R1#76 | GGGGCGCAGGTAGCAACTAAGATATCTCGGGACGTGCTGA |
| R1#77 | TTCGGAGAGTGCTGTTCCTGGGCATTCCCGGTGACTGCCC |
| R1#78 | CGCCTCCCCGTGGCTCCCATAATTCACGTCAAGCTGAAAC |
| R1#79 | CGCCGCCTATCGGTCTCATGGATTTACGCCTCGCCTCTTT |
| R1#80 | CGCCCAACGTCGTACCCTGGTCCCCATTCAAGGCGAATGT |
| R1#81 | GGTTTCGTGCGCTAAAGTGGCCAACACGACGTTTCTTGCG |
| R1#82 | GTAGCAGCACAGCACTCCGGCTGAACGCAGGCAGCGGGTG |
| R1#83 | ACCGTGGTCCGGAATGCTTACCTCGACGTCCCTCACGCC |
| R1#84 | CCGGTGATCGTCTGACCGATAGCGGATTATGCCCACCGGC |
| R1#85 | AGGGCGTATGTCGATCCCAGCCAAAACCTAGACTCTATAG |
| R1#86 | GGCGTGGCATCCACCGTCTTTGTACGGACATCTGCGTGCA |
| R1#87 | ATGCTAAGGGAATAATAGATTCCGTGGGTCACCCATTGAT |
| R1#88 | CCAATAGACTAGTCTTTAGTTTCTGTATGGGTGTCGGGA |
| R1#89 | TGGTGTGCTCCCACGGGGCTAGAAACCCCCCTTCTGGTGC |
| R1#90 | TAACGGGGTACACCGTGATGTTCTGATAAGTCGCCCACGC |
| R1#91 | AGGAATGGGCTCGGCTATGCATGTCTAAGCATCCATCGGA |
| R1#92 | GGAGTCCGGACGAAAAGCGATCATTGATCACACCGGACCT |
| R1#93 | GGGCCCCTGTCGGCGTACTTACTTTGCATTTTCTGCGCAC |
| R1#94 | GGTCGATGGCAGATAGTCTTAGATCACGATACTGGCGCCG |
| R1#95 | GCGCTGAACTATAACAAAAGCCGTCGCCGGTTCGTTGATT |
| R1#96 | CAGTTGCCGGTAGTACCGTATCTGCGATGTGGCACCCGTA |
| R1#97 | CGTTGGTTAGCATTCGGGCTTAGAAGCCGGATGTTGTGTG |
| R1#98 | CTGTTGCCCACTACTAAAGTCTGTTGTATGTCGCAAATCC |
| R1#99 | AGCCGTGTGGTAGATCGTAGGAACTCGACTTCTTTCGTCA |
| R1#100 | TCGTTAAGGGATGCATAGATTACAGAAGGCTAAACGCCCA |
| R2#1 | CGAGCTCAGCTAGGTACTACGCCCCGTGGTGCGAGGTCAT |
| R2#2 | GGACCTAAGGGCTACGTGTCAACAGGCCGCCGGCAAGTG |
| R2#3 | CAGGTGATTTGCCACGAGAGTCATACAGTCCTTGTGTGGG |
| R2#4 | ATCTCCGATTGCCCCACGACGCAGTGGTCGGAGTTACTTT |
| R2#5 | GCACCTGAGTCATGGACAGTCCAGGGAGTGACACCGAGCC |
| R2#6 | CATCACAGTCTCTGTAACGCATCCACGCCCTTTGCGGTAG |
| R2#7 | CAGGCAGATGGCATCATACAGTCTTTGTCGGCATACGTGG |
| R2#8 | GCGCTGCTATCCAGTGGAAAGTAGCTCTCTTGTCGGGTCT |
| R2#9 | GGGGGGCGCACTTTGCGTGTCAAATAGTGCCCGACTTGTA |
| R2#10 | TTGACCTTCCCGCTAACGTGAAGAGTCGGGGACCACCCTA |
| R2#11 | GTCATTGTTGCTTTGCGGGAGCGCAAATCAGACTGGTCCT |
| R2#12 | TCAGTAACGCGAATATGCATCGGCGTCAGACAGTCTACGG |
| R2#13 | GCGATCGCGTTGTTGCCAGGCCCAATCCCATGAATCGTAG |
| R2#14 | GCCAGAACGCACCGATCGCAGGTTTCTGTGAGCAGGGGG |
| R2#15 | GCGATCGCGTCGTTGCCAGGCCCAATCCCATGAATCGTAG |
| R2#16 | AATAGCCCCCTGACGCAAGGGTTAGAGCAGTAATCATGGT |
| R2#17 | TCGTATTGGTTTTTCGTGAGTCAACAGTCCCCAACTAGGC |
| R2#18 | TCCCGAGAGGATGGAGGCCGCCGCCATATATTACCTGTG |
| R2#19 | CTTGACGCTTCGGGGACCCGACTGTGTTTCTAAACTGTTG |
| R2#20 | GGGCCGTCCTTAGTATGCGTATGCCCGTACGATGTCCCTAT |
| R2#21 | CGGGCTAGCAATTCCGTGGCTCAAACAATCGGGGTACTAG |
| R2#22 | AACCGCTATCCGTTGAGCCAGTACGCGTTCTCCCGTCTGC |
| R2#23 | CTCCATGGTCAGTCCGCTGCGTAGTCTGGTATGGTACAT |
| R2#24 | CTGGCGTATTCGGACCGCTGGGTGACGCAACCCTCCTAAT |
| R2#25 | GTCAATAATAATCACTGTTGTTGTTCAGTCCTAGCCTATG |
| R2#26 | ATGGAAGTTTGTCAATGTCAGGGATAATCTCAGGGTGGTA |
| R2#27 | GGAGGCCTAAGTCACAGAGGAAAGCCGGCTGGTGGACACT |
| R2#28 | GTTGCAGGTTCTACGCGGTCACAGTACAGTCATGACGT |
| R2#29 | TGTACGGGTTCACACAGTACCGTCGTATAGGGCGGGACTT |
| R2#30 | TAGGGCGATTAACGCAACCGGCGCAGGTTTTAAACGCGCT |
| R2#31 | CATTCATAGTCCAGCCGAGTTGCTAACTGTTAATTCTGGA |
| R2#32 | TTGATTGCCACCCGCCGCAGGGTTCAAAAGCCGCATTGCT |
| R2#33 | AGACTCGGGGCCAGACTGCAGATTGACTGCTATTTGGATT |
| R2#34 | GTGTAGGATCATCGCCCCCACGACGCAGTGGGTGATCGTA |
| R2#35 | CACCGCTATCCGCCGCAGGATTGGGAGCATAGATACACGC |
| R2#36 | AAAGTCGAGGCTGGGTGCCACGCCGCCAAATACCCACAG |
| R2#37 | GACTCCGGGACATGCCCATGTCGCAATGGTGGCCCGGATT |
| R2#38 | GTTCGATTGCCGGGCGACGCAGCCCTCGAACTCTCAGACG |
| R2#39 | GCGATCGCGTCGTTGCCAGGCCCAACCCCATGAATCGTAG |
| R2#40 | GATCCCCGTGTAACCGCTTTTCGCCGCAGAAATTACACTT |
| R2#41 | CGAGCTCAGCTAGGTACTATGCCCCGTGGTGCGAGGTCAT |
| R2#42 | ATGGGCGCTGATCGTAACCTGTAAGGGTTTAATACCAGTT |
| R2#43 | CGTCACTGGTCTTACTAGGCCGAGCAGTTCATCTTGAT |
| R2#44 | TGATGAGCTGCCGTCAGCTTTCGGTTAACGGCACGTTGTT |
| R2#45 | AGGGCGTCCGACGTCACAAGAGACAATCTCGTCTCTGTAC |
| R2#46 | CGCGAAACCGAATGCCCAAGTTACCAAGCAATACTTCGCT |
| R2#47 | CGGGTGATCCGTGCACCGGGTCAACCGGGAGCCGGTATGG |
| R2#48 | CATGGCGGACCAGACGAATATTTCTCATATAGTTAACGCA |
| R2#49 | TCCACGGAATTGCTATAGGGAGACAATAAAACCCCATACC |
| R2#50 | TATCCTCACAGACCGCCGACACGGGCACAGGCCAGGTCTG |
| R2#51 | ACTCGCGGACGACACTCCGGCCGTCGGGATGCAGGGTAGG |
| R2#52 | ATACAGACTCAGGACCTTCATTTGGTACAAGCCCCAGTAC |
| R2#53 | CGCCACTGTTTGAGGTTATTTCTCCATTACATGGGTCAGT |
| R2#54 | GAGATAATCCACTGCTGGTTACGTTCCAGCGTAGGGGT |
| R2#56 | TAAGAGTTAGATTTCTGAAGTACACACGTACGATCGTTTT |
| R2#57 | GGGGGTGAACCGCAGCCGTGCGAACGCGCTTTGGGCACCG |
| R2#58 | CGACGTGACACCGTCTACCTGCCATCGCTATAGGACGGAG |
| R2#59 | TTGCCATATTCTATGCCCCCCAAGTAGGTGGGCTCATAT |
| R2#60 | GAGTGTAGTGTTTGGAACGATGAAGTGCCTACTCCAAGGC |
| R2#61 | CCGGGAGCACAAATAGTCCCCAACATGACCCCCCGAGGT |
| R2#62 | CGTACGCTAGCCATTTATCAATGTATCGGGCATATGGGG |
| R2#64 | TACGGTACGGGGGCCAGTGGGCAGCTTCGGGCGGGAGGGC |
| R2#65 | AGTTCAGGTGTGCTTGGAGTGGAGTAGAGGGGGCACGGAG |
| R2#66 | TCGTAGTATTAGGAGCTCCTAGTACAACGTTTTTGCTATA |
| R2#67 | GTGCTGTAAGCCATGGATTATTTGTTCGGACTGTTAGGAT |
| R2#68 | TTGTTCAAGCTTCTGCTGCCGGATGCGTGGGTACACCGGT |
| R2#69 | CCGGCCCGGTACTTACTGTGGGGCAGTGGAGCTAGACT |
| R2#70 | CCGTACTGGCGCGGTCAGGGGATTGGAATCAGCTAGGTA |
| R2#71 | AAGGTTGATGGCAACTTTGCAAGCGCCGGTCTATGGGCTT |
| R2#72 | GTGCGAGGCTACTGGGTTATTGTCCCGCGCAGGGTAATTC |
| R2#73 | TTACAAACTGTTAAGTTTCCACACGTTGCAGTTTGCCTGT |
| R2#74 | GATTACGGGGTCTCAGGATTATGCGATCGGTAAGGCAAGG |
| R2#75 | TACTCGTTAATACCCGGGCGAGCACAGCTGTAATTTCTT |
| R2#76 | CCCGCTTAAATGGTGTGGACCTGTCGTGTTAGACCCCTGT |
| R2#77 | TCTGCAGTGCTGTACGCGGCTTCGAAGTTTAGTGATAAAT |
| R2#78 | GATGACCGTGCACGGAGTAGAAGTAGGATTAGGGGCAAAT |
| R2#79 | CCGGGTTGCTAGTATCATGGGGGTCGGGGGAACCGGAGGC |
| R2#80 | GGGGTTCAATCACGTGACTTGATACCCATGGGGGCCAGTC |
| R2#81 | GATCGAAGGACGGCAAGCACTAGTTGGGATCCGCTCGTAC |
| R2#82 | AAGAACAGCAAAATGTGTTATAAATTCACGTGCTTTTACT |
| R2#83 | TTGTTCAGGCCTGTTACATTGTTGTTATAGGGTGCTGCAC |
| R2#84 | TCCTAGATTTGTGTTCAAAGCCTCGGCAGCGTCGACCTC |
| R2#85 | CGGTGACTCCATGGGGTAGATCTCGAATGTGGTTATGACA |
| R2#86 | TAGTAGTCCACATCTCTGGTCCGTGGCTGAAGTCCGCTAG |
| R2#87 | AATCGTATTAGTAGTAACGAAACCATCCCTATATGACGGT |
| R2#88 | CTGATTCTGCCACGTATAGATAACTAGTCCTTCGGTTACG |
| R2#89 | CCCCAATGGAATTTGGGCCGGGGCATGTGAGACATTGCGA |
| R2#90 | CCATAAGGATTACTCGACTAACCGAGATACTCCCGTACAC |
| R2#91 | CCTGGTGCATCTTGCAGGTGTCCTCGTAAAGGCCTCCT |
| R2#92 | CGATGCAGTCTGAATGCGATTACAATCAACGGCTCACCTA |
| R2#93 | GGGATCGGACATTTTATCTCGGGTTTCTCTTCGCGGGC |
| R2#95 | ACGGGCCTTTTGAGTAGCCTTTGCATTGGGGAACGTTTCT |
| R2#96 | GCTTGCGGGGCTACTGGCTTAAACGTTCGCGCTGCTGCAGC |
| R2#97 | CCAACTTCGGTTTCGGCGTAGATCCCACCGAGGGTGACTA |
| R2#98 | TCACCGCTCTATGACCTTGTTGGTGACAGGTCGTCAGCTA |
| R2#99 | GGTCAATCCTTCAGCTTCGTCCAGGGGTACCTAGGTTGTT |
| R2#100 | AGCACACGCCCCAAACTGCCCCTAGATAACGAGCACCGAA |
| R3#1 | ATCGCCCCCTGACGCAAGGGTAGAGCTACTCATTCGGTCG |
| R3#2 | ACGCGCCGATCGCAGGCTAGAGCTAATGGCTCAGGTTTAT |
| R3#3 | GCACCTGAGTCATGGACAGTCCAGGGAGTGACACCGAGCC |
| R3#4 | CGAGCTCAGCTAGGTACTACGCCCCGTGGTGCGAGGTCAT |
| R3#5 | CAGACGCACCTGGCGCAAGGTTCTGAAGATCAGTTACGTG |
| R3#6 | CCTATGACGCGTCCGACGCAGGACTCATACGGGTGCTCGT |
| R3#7 | AGTGAGTTGCCCCCCGACGCAGGGGCTCACGCTTGCTTA |
| R3#8 | TCGCGCCGATCGCAGGCAAGAGCTCTCCCGTCACGAGTGG |
| R3#9 | GCGCTGCTATCCAGTGGAAAGTAGCTCTCTTGTCGGGTCT |
| R3#10 | CAGGTGATTTGCCACGAGAGTCATACAGTCCTTGTGTGGG |
| R3#11 | CAGGCAGATGGCATCATACAGTCTTTGTCGGCATACGTGG |
| R3#12 | CACGCGACCGGCGCAGGTCTGAGGGCAGGTCCAGTAATAT |
| R3#13 | GTTATTGGAGTTGCCCCGTGACGCAACGGCTCCAAAACTGA |
| R3#14 | CCACGATTGCTCCCCAACGCCGGGGTCGGGGAAACGCGTC |
| R3#15 | GGGGGGCGCACTTTGCGTGTCAAATAGTGCCCGACTTGTA |
| R3#16 | GGGCCGTCCTTAGTATGCGTATGCCCGTACGATGTCCCTAT |
| R3#17 | CATCACAGTCTCTGTAACGCATCCACGCCCTTTGCGGTAG |
| R3#18 | TCGCCAATGTGACGCTTCCGGCGCAGGAATCACATTCTTC |
| R3#19 | ACTTGGACCCGATGCCCGTGGCGCAACGGTCGGGTCCTTC |
| R3#20 | GCTCCGGACATCGCTACCCGACGCAGGGTTGTCCTTGGTTA |
| R3#21 | ATCGCCCCCTGACGCAAGGGTAGAGCTACTCATTCGGTCA |
| R3#22 | ATCGTTGACCAGGACGCGTTCGGCGCAGAACTCATGGTCA |
| R3#23 | CTACATGAGGTTCTCAGATGCCCCTGTCGCAAGGGTCTG |
| R3#24 | ATCGCCACCTGACGCAAGGTTAGACCAGGGACCGATGCGC |
| R3#25 | GGACCTAAGGGCTACGTGTCAACAGGCCGCCGGCAAGTG |
| R3#26 | ATCATTCACGCGGCCGACGCAGGCCTGAAAGATATCCTCG |
| R3#27 | CCCGTGGATTAACGCTTATGTCGCAATAATTAATCTCGCG |
| R3#28 | CTTGAATCCACGCGCCGATCGCAGGCTGGATTTAGACCGC |
| R3#29 | CCGACGCACCCGGCGCAGGGTTCGGAGATCTAGTTAATTA |
| R3#30 | GCGATCGCGTCGTTGCCAGGCCCAATCCCATGAATCGTAG |
| R3#31 | ACACACGCGGATAGGCGCATCCTGGGTAGGGGATTACGAA |
| R3#32 | ATCGCCCCCTGACGCAAGGGTAGAGCTACTCATTCGGTCT |
| R3#33 | CCGGGCGCCGTGCTTCGGACGCTCGTATTGCTCGATCCGG |
| R3#34 | ATCGCCCCCTGACGCAAGGGTAGAGCTACTCATTCGGTCC |
| R3#35 | CATGAACGCTCCGGTTGCAGGATTCATGAAGCGCGTTAA |
| R3#36 | ACGCGCCGATCGCAGGCTATGGGCGGGCTCGGAGTCATTT |
| R3#37 | GCGATCGCGTTGTTGCCAGGCCCAATCCCATGAATCGTAG |
| R3#38 | CACGCTACTGTCGCAAGTATGAGAGCGGATCAGCGTAGT |
| R3#39 | CTTCCGATGAGTACGCCCCGATCGCAGGGTACTGACTAGG |
| R3#40 | CAGTGGACATTCGCGCCGATCGCAGGCAATGACTATGGAG |
| R3#41 | AACCGCTATCCGTTGAGCCAGTACGCGTTCTCCCGTCTGC |
| R3#42 | GAACGCACGGATCGCCGTTTCAGGGCCGGTGCAGTGCTAC |
| R3#43 | CCCGTGGATTAACGCTTATGTCGCAATAATTAATCTCGTG |
| R3#44 | TTGACCTTCCCGCTAACGTGAAGAGTCGGGGACCACCCTA |
| R3#45 | CGAGCTCAGCTAGGTACTATGCCCCGTGGTGCGAGGTCAT |
| R3#46 | ATCAGCAGGGTGGACTTCGATGTCGTTGTGCAAACCGGTC |
| R3#47 | ATGGAAGTTTGTCAATGTCAGGGATAATCTCAGGGTGGTA |
| R3#48 | GGCAGGGAGAGTGGCGCACGAACTAATGTTCAGGTTGTTG |
| R3#49 | CGATGAGGATCAATCGCTGTCCGGCGCAGGACTTGTCCTC |
| R3#50 | GTTGCAGGTTCTACGCGGTCACAGTACAGTCATGACGT |
| R3#51 | GTCATTGTTGCTTTGCGGGAGCGCAAATCAGACTGGTCCT |
| R3#52 | GTCAGGCGCACCGGTCGCAGGTCCTGACAGTGAGCCCTAC |
| R3#53 | CGGGCTAGCAATTCCGTGGCTCAAACAATCGGGGTACTAG |
| R3#54 | TCAGTAACGCGAATATGCATCGGCGTCAGACAGTCTACGG |
| R3#55 | ATGGACCGCTATCCGCCGCAGGATTCCAGGCTTTCTGGGA |
| R3#56 | TACGCGCCGATCGCAGGCTAAGCGGGGGCGGGGGAAGAG |
| R3#57 | TATAGTAGAAGGCTTCACGCGCGTCCGGCGCAGGACCGTG |
| R3#58 | ATCGCCCCCTGACGCAAGGGTAGAGCTACTCATCCGGTCG |
| R3#59 | GGAGGCCTAAGTCACAGAGGAAAGCCGGCTGGTGGACACT |
| R3#60 | CACGCTACTGTCGCAAGTATGAGAGCGGATCAGCGTAGG |
| R3#61 | ATCGCCCCCTGACGCAAGGGTAGAGCTACTCATTCGGTTG |
| R3#62 | ATGCCCCGGTCGCAGGGTAGAGTATATTAGGTGTACACCA |
| R3#63 | GGTCAATCCTTCAGCTTCGTCCAGGGGTACCTAGGTTGTT |
| R3#64 | CAGCGAGTAGGCGGGCAGTTTCGCGCCGATTGCAGGCAAAC |
| R3#65 | ATCGCCCCCTGACGCACGGGTAGAGCTACTCATTCGGTCG |
| R3#66 | GCTTGCGGGGCTACTGGCTTAAACGTTCGCGCTGCTGCAGA |
| R3#67 | ATCGCCCCCTGACGCAAGGGGAGAGCTACTCATTCGGTCG |
| R3#68 | TCCCGAGAGGATGGAGGCCGCCGCCATATATTACCTGTG |
| R3#69 | CTTGACGCTTCGGGGACCCGACTGTGTTTCTAAACTGTTG |
| R3#70 | TCGTCGGGAGCCGGGGTAAGATAGTCCTCTGGCCATCTGG |
| R3#71 | GTGACGCGGTTGACGCAGACCTCACTGTGGGCAGTAACGG |
| R3#72 | GACGCTTATGTCGCAATAATCAGTAGTTGACCGATGTCTC |
| R3#73 | ATCGCCCCCTGACGCAAGGGTAGAGCTACTCATTCGGCCG |
| R3#74 | GGGGGGCGCATTTTGCGTGTCAAATAGTGCCCGACTTGTA |
| R3#75 | ATCGCCCCCTGACGCAAGGGCAGAGCTACTCATTCGGTCG |
| R3#76 | TAATCGGCCTGGCGCCCGTATTGCTCGGACATGCCGAA |
| R3#77 | AGAACGCTAATGTCGCAATTATTCATTGGTCGGGTATGGG |
| R3#78 | TATTCCCGTGAGCACGCCCGTGTTGCTCGGTGCTCTCAAT |
| R3#79 | ATCGCCCCCTGGCGCAAGGGTAGAGCTACTCATTCGGTCG |
| R3#80 | GGCACTCAGCTTCCGGTGATAACTTCATCCTACTGATGGT |
| R3#81 | AATTGCTCCCCTGACGCAAGGGGTTAGACTTACGATTCC |
| R3#82 | CTCCATGGTCAGTCCGCTGCGTAGTCTGGTATGGTACAT |
| R3#83 | ACGCGCCGATCGCAGGCTAGAGCTAATGGCTCCGGTTTAT |
| R3#84 | TGTGCTCAGAGGCTTCGCGTACGGCGCAGTACAAGTCCTA |
| R3#85 | AACTTTCCGCTAATCGCCGCAGATTAAAGTTATTGCTGCT |
| R3#86 | ATCGCCCCCTGACGCAAGGGTAGAGCTACTCCTTCGGTCG |
| R3#87 | CGGTCGCGTCCGTCGCAGGACACCGTTTTGCAGAAGGTAA |
| R3#88 | ATCTCCGATTGCCCCACGACGCAGTGGTCGGAGTTACTTT |
| R3#89 | ACGCGCCGATCGCAGGCTAGAGCTAATGGCTCAGGCTTAT |
| R3#90 | TGATCGCCCGGACGCGTGTGACGCAACACTCCGAGACGAA |
| R3#91 | ATCGCCCCCTGACGCAAGGGTAGAGCTACTCGTTCGGTCG |
| R3#92 | GAGCTTACGCGTGATTGGGTCACTAAGCTAAGGGTTCGGG |
| R3#93 | ATTGCCCCCTGACGCAAGGGTAGAGCTACTCATTCGGTCG |
| R3#94 | ACCGCCCCCTGACGCAAGGGTAGAGCTACTCATTCGGTCG |
| R3#95 | TGCCGATCCTCGTACGTTGGTGACACTAGCCGTGGCAGTG |
| R3#96 | CAAATCACGCGCCGGTTGCAGGCTGATCTGCGGCTTCCTA |
| R3#97 | TGTGATTCTAGGTGAACGCAATGATCGCAATTTTCACCGA |
| R3#98 | ATCGCCCCCTGACGCAAGGGTAGAGCTACTCATTCGGTAC |
| R3#99 | AATATAACGCTCCCGACGCAGGGATTATATTAAGTCCGTT |
| R3#100 | GCAATACTCGTTAAAAGATAGTATTGCCCTCTGGTTGAGT |
| R4#1 | ATCGCCCCCTGACGCAAGGGTAGAGCTACTCATTCGGTCG |
| R4#2 | ACGCGCCGATCGCAGGCTAGAGCTAATGGCTCAGGTTTAT |
| R4#3 | CAGACGCACCTGGCGCAAGGTTCTGAAGATCAGTTACGTG |
| R4#4 | CACGCGACCGGCGCAGGTCTGAGGGCAGGTCCAGTAATAT |
| R4#5 | TCGCGCCGATCGCAGGCAAGAGCTCTCCCGTCACGAGTGG |
| R4#6 | AGTGAGTTGCCCCCCGACGCAGGGGCTCACGCTTGCTTA |
| R4#7 | ACTTGGACCCGATGCCCGTGGCGCAACGGTCGGGTCCTTC |
| R4#8 | GTTATTGGAGTTGCCCCGTGACGCAACGGCTCCAAAACTGA |
| R4#9 | GCTCCGGACATCGCTACCCGACGCAGGGTTGTCCTTGGTTA |
| R4#10 | CCTATGACGCGTCCGACGCAGGACTCATACGGGTGCTCGT |
| R4#11 | TCGCCAATGTGACGCTTCCGGCGCAGGAATCACATTCTTC |
| R4#12 | CCACGATTGCTCCCCAACGCCGGGGTCGGGGAAACGCGTC |
| R4#13 | ATCGTTGACCAGGACGCGTTCGGCGCAGAACTCATGGTCA |
| R4#14 | CTTGAATCCACGCGCCGATCGCAGGCTGGATTTAGACCGC |
| R4#15 | CTTCCGATGAGTACGCCCCGATCGCAGGGTACTGACTAGG |
| R4#16 | ATCGCCACCTGACGCAAGGTTAGACCAGGGACCGATGCGC |
| R4#17 | CTACATGAGGTTCTCAGATGCCCCTGTCGCAAGGGTCTG |
| R4#18 | ATCATTCACGCGGCCGACGCAGGCCTGAAAGATATCCTCG |
| R4#19 | CAGTGGACATTCGCGCCGATCGCAGGCAATGACTATGGAG |
| R4#20 | GAACGCACGGATCGCCGTTTCAGGGCCGGTGCAGTGCTAC |
| R4#21 | CATGAACGCTCCGGTTGCAGGATTCATGAAGCGCGTTAA |
| R4#22 | ATCGCCCCCTGACGCAAGGGTAGAGCTACTCATTCGGTCA |
| R4#23 | GTCAGGCGCACCGGTCGCAGGTCCTGACAGTGAGCCCTAC |
| R4#24 | CCGACGCACCCGGCGCAGGGTTCGGAGATCTAGTTAATTA |
| R4#25 | GACGGTGCTGCTGTACTTGGGTGTTGCGCCACACGCGCC |
| R4#26 | ACGCGCCGATCGCAGGCTATGGGCGGGCTCGGAGTCATTT |
| R4#27 | ACGCGCCGATCGCAGGCTAGAGCTAATGGCTCAGGGTTAT |
| R4#28 | ACACACGCGGATAGGCGCATCCTGGGTAGGGGATTACGAA |
| R4#29 | GTCGCGGGTGGCGCAACCCACAGAGAAATAGTCGGCGATA |
| R4#30 | CCGGGCGCCGTGCTTCGGACGCTCGTATTGCTCGATCCGG |
| R4#31 | CACGCTACTGTCGCAAGTATGAGAGCGGATCAGCGTAGT |
| R4#32 | ATCAGCAGGGTGGACTTCGATGTCGTTGTGCAAACCGGTC |
| R4#33 | GTGACGCGGTCGACGCAGACCTCACTGTGGGCAGTAACGG |
| R4#34 | CACGCTACTGTCGCAAGTATGAGAGCGGATCAGCGTAGG |
| R4#35 | ATCGCCCCCTGACGCAAGGGTAGAGCTACTCATTCGGTCT |
| R4#36 | CCCGTGGATTAACGCTTATGTCGCAATAATTAATCTCGCG |
| R4#37 | CGATGAGGATCAATCGCTGTCCGGCGCAGGACTTGTCCTC |
| R4#38 | ATCGCCCCCTGACGCAAGGGTAGAGCTACTCATTCGGTCC |
| R4#39 | GAGCTTACGCGTGATTGGGTCACTAAGCTAAGGGTTCGGG |
| R4#40 | ACGCGCCGATCGCAGGCAAGAGCTCTCCCGTCACGAGTGG |
| R4#41 | ATCGCCCCCTGACGCAAGGGTAGAGCTACTCATCCGGTCG |
| R4#42 | AGTGAGTTGCTCCCCGACGCAGGGGCTCACGCTTGCTTA |
| R4#43 | TATTCCCGTGAGCACGCCCGTGTTGCTCGGTGCTCTCAAT |
| R4#44 | ATCGCCCCCTGACGCAAGGGCAGAGCTACTCATTCGGTCG |
| R4#45 | TATAGTAGAAGGCTTCACGCGCGTCCGGCGCAGGACCGTG |
| R4#46 | GCCAGAACGCACCGATCGCAGGTTTCTGTGAGCAGGGGG |
| R4#47 | ATCGCCCCCTGACGCAAGGGGAGAGCTACTCATTCGGTCG |
| R4#48 | ACGCGCCGATCGCAGGCTAGAGCTAATGGCTCCGGTTTAT |
| R4#49 | CCCGTGGATTAACGCTTATGTCGCAATAATTAATCTCGTG |
| R4#50 | ACGCGCCGATCGCAGGCTAGAGCTAATGGCTCAGGCTTAT |
| R4#51 | CACGCGACCGGCGCAGGTCTGAGGGCAGGTCCAGTAATAC |
| R4#52 | TCCAGGGAATGACGCTCGTGTTGCTCGATCACCCTCAAGG |
| R4#53 | ATCTCCGATTGCCCCACGACGCAGTGGTCGGAGTTACTTT |
| R4#54 | ATGGACCGCTATCCGCCGCAGGATTCCAGGCTTTCTGGGA |
| R4#55 | ACGCGCCGATCGCAGGCTAGAGCTAATGGCTCAGGTTTGT |
| R4#56 | CAGACGCACCTGGCGCAAGGTTCTGAGGATCAGTTACGTG |
| R4#57 | CAGCGAGTAGGCGGGCAGTTTCGCGCCGATTGCAGGCAAAC |
| R4#58 | ACGCGCCGATCGCAGGCTAGAGCTAATGGCTCAGGTCTAT |
| R4#59 | CAGACGCACCTGGCGCAAGGTTCTGAAGATCAGTTACGCG |
| R4#60 | AATTGCTCCCCTGACGCAAGGGGTTAGACTTACGATTCC |
| R4#61 | ATCGCCCCCTGACGCACGGGTAGAGCTACTCATTCGGTCG |
| R4#62 | GTTGCGACGCGTCCGACGCAGGACTCGCACATTAGTTGAC |
| R4#63 | ATCGCCCCCTGACGCAAGGGTAGAGCTACTCATTCGGTTG |
| R4#64 | GTGACGCGGTTGACGCAGACCTCACTGTGGGCAGTAACGG |
| R4#65 | TCCGTGGTGACCAGTGCTGAGCGCTCGTGTTGCTCGACG |
| R4#66 | ACGCGCCGATCGCAGGCTAGAGCTAATGGCTCAGGTTTAC |
| R4#67 | TACGCGCCGATCGCAGGCTAAGCGGGGGCGGGGGAAGAG |
| R4#68 | CACGCGACCGGCGCAGGTCTGAGGGCAGGTCCAGTAGTAT |
| R4#69 | GCTTGACCGAACGCACGGATCGCCGTTTCGGAGATAATTG |
| R4#70 | GTTATTGGAGTTGCCCCGTGACGCAACGGCTCCCAAACTGA |
| R4#71 | CGGTCGCGTCCGTCGCAGGACACCGTTTTGCAGAAGGTAA |
| R4#72 | AATAGGATGCCCTCGTCGCAGAGGTCCTATTATCCAGG |
| R4#73 | ATCGCCCCCTGACGCAAGGGTAGAGCTACTCGTTCGGTCG |
| R4#74 | GTTATTGGAGTTGCCCCGTGACGCAACGGCTCCAAAGCTGA |
| R4#75 | ACGCGCCGATCGCAGGCTAGAGCTAATGGCTCAGGATTAT |
| R4#76 | ATCGCCCCCTGACGCAAGGGTAGAGCTACCCATTCGGTCG |
| R4#77 | ATCGCCCCCTGACGCAAGGGTAGAGCTACTCATTCGGCCG |
| R4#78 | GTTATTGGAGTTGCCCCGTGACGCAACGGCTCCAACACTGA |
| R4#79 | ACGCGCCGATCGCAGGCTAGAGCTAATGGCTCAGGTTCA |
| R4#80 | CAGACGCACCTGGCGCAAGGCTCTGAAGATCAGTTACGTG |
| R4#81 | ATCGCCCCCTGACGCAAGGGTAGAGCCACTCATTCGGTCG |
| R4#82 | CAAAGCGCGGCCGGCGCAGGCCCTTTGGAGCAGTCTTAGG |
| R4#83 | CCTATGACGCGTCCGGCGCAGGACTCATACGGGTGCTCGT |
| R4#84 | ACAGGCCCTGGGGGGCTATCACGCACCCGGCGCAGGGTTG |
| R4#85 | GAACGCACGGATCGCCGTTTCAGGGCCGGTGCAGTGCTAT |
| R4#86 | AGATAACTGGCGCGTCCGTCGCAGGACCCAGTTACCTCA |
| R4#87 | GTTATTGGAGTTGCCCCGTGACGCAACGGCTCCACAACTGA |
| R4#88 | GTTATTGGAGTTGCCCCGTGACGCAACGGCTCCAAAACTGG |
| R4#89 | ACGCGCCGATCGCAGGCTAGAGCTAATGGCTCAGGTTGAT |
| R4#90 | TGATCGCCCGGACGCGTGTGACGCAACACTCCGAGACGAA |
| R4#91 | CAGACGCACCTGGCGCAAGGTTCTGAAGATCAGTTACGTA |
| R4#92 | ATCATTCACGCGGCCGACGCAGGCCTGAAAGATATCCCCG |
| R4#93 | ACTTGGACCCGATGCCCGTGGCGCAACGGTCGGGGCCTTC |
| R4#94 | ACGCGCCGATCGCAGGCTAGAGCTAATGGCTCAGGTTTCT |
| R4#95 | AACTTTCCGCTAATCGCCGCAGATTAAAGTTATTGCTGCG |
| R4#96 | AACTTTCCGCTAATCGCCGCAGATTAAAGTTATTGCTGCT |
| R4#97 | ATCGCCCCCTGACGCAAGGGAAGAGCTACTCATTCGGTCG |
| R4#98 | ATCGCCCCCTGACGCAAGGGTAGAGCTACTCATTTGGTCG |
| R4#99 | TTCGTTGCCCATACGACGCAGTATGCGAAAGACAAATAGT |
| R4#100 | ACCGCCCCCTGACGCAAGGGTAGAGCTACTCATTCGGTCG |
| R5#1 | ACGCGCCGATCGCAGGCTAGAGCTAATGGCTCAGGTTTAT |
| R5#2 | ATCGCCCCCTGACGCAAGGGTAGAGCTACTCATTCGGTCG |
| R5#3 | CAGACGCACCTGGCGCAAGGTTCTGAAGATCAGTTACGTG |
| R5#4 | CACGCGACCGGCGCAGGTCTGAGGGCAGGTCCAGTAATAT |
| R5#5 | ACTTGGACCCGATGCCCGTGGCGCAACGGTCGGGTCCTTC |
| R5#6 | GTTATTGGAGTTGCCCCGTGACGCAACGGCTCCAAAACTGA |
| R5#7 | TCGCCAATGTGACGCTTCCGGCGCAGGAATCACATTCTTC |
| R5#8 | AGTGAGTTGCCCCCCGACGCAGGGGCTCACGCTTGCTTA |
| R5#9 | ATCGCCCCCTGACGCAAGGGTAGAGCTACTCATTCGGTCA |
| R5#10 | CCGACGCACCCGGCGCAGGGTTCGGAGATCTAGTTAATTA |
| R5#11 | ATCATTCACGCGGCCGACGCAGGCCTGAAAGATATCCTCG |
| R5#12 | ATCTCCGATTGCCCCACGACGCAGTGGTCGGAGTTACTTT |
| R5#13 | CATGAACGCTCCGGTTGCAGGATTCATGAAGCGCGTTAA |
| R5#14 | ATCGCCACCTGACGCAAGGTTAGACCAGGGACCGATGCGC |
| R5#15 | TCGCGCCGATCGCAGGCAAGAGCTCTCCCGTCACGAGTGG |
| R5#16 | CAGTGGACATTCGCGCCGATCGCAGGCAATGACTATGGAG |
| R5#17 | CTTGAATCCACGCGCCGATCGCAGGCTGGATTTAGACCGC |
| R5#18 | ACGCGCCGATCGCAGGCTAGAGCTAATGGCTCAGGGTTAT |
| R5#19 | ACGCGCCGATCGCAGGCTATGGGCGGGCTCGGAGTCATTT |
| R5#20 | GATTGCCCCTCGACGCAGAGGTCTGAGTTGGTACAAACTC |
| R5#21 | ATCGCCCCCTGACGCAAGGGTAGAGCTACTCATTCGGTCT |
| R5#22 | GTGACGCGGTCGACGCAGACCTCACTGTGGGCAGTAACGG |
| R5#23 | CACGCGACCGGCGCAGGTCTGAGGGCAGGTCCAGTAATAC |
| R5#24 | ATCGCCCCCTGACGCAAGGGTAGAGCTACTCATTCGGTCC |
| R5#25 | CCACGATTGCTCCCCAACGCCGGGGTCGGGGAAACGCGTC |
| R5#26 | GAACGCACGGATCGCCGTTTCAGGGCCGGTGCAGTGCTAC |
| R5#27 | ACGCGCCGATCGCAGGCTAGAGCTAATGGCTCAGGTTTAC |
| R5#28 | CAGACGCACCTGGCGCAAGGTTCTGAAGATCAGTTACGCG |
| R5#29 | ACGCGCCGATCGCAGGCTAGAGCTAATGGCTCCGGTTTAT |
| R5#30 | ATCGTTGACCAGGACGCGTTCGGCGCAGAACTCATGGTCA |
| R5#31 | ACGCGCCGATCGCAGGCTAGAGCTAATGGCTCAGGCTTAT |
| R5#32 | ACGCGCCGATCGCAGGCTAGAGCTAATGGCTCAGGTTTAA |
| R5#33 | GCTCCGGACATCGCTACCCGACGCAGGGTTGTCCTTGGTTA |
| R5#34 | CAGACGCACCTGGCGCAAGGTTCTGAAGATCAGTTACGTA |
| R5#35 | ATCGCCCCCTGACGCAAGGGTAGAGCTACTCATTCGGTTG |
| R5#36 | AGTGAGTTGCTCCCCGACGCAGGGGCTCACGCTTGCTTA |
| R5#37 | AATTGCTCCCCTGACGCAAGGGGTTAGACTTACGATTCC |
| R5#38 | CAGACGCACCTGGCGCAAGGTTCTGAGGATCAGTTACGTG |
| R5#39 | GTTCGATTGCCGGGCGACGCAGCCCTCGAACTCTCAGACG |
| R5#40 | GACGGTGCTGCTGTACTTGGGTGTTGCGCCACACGCGCC |
| R5#41 | ACACACGCGGATAGGCGCATCCTGGGTAGGGGATTACGAA |
| R5#42 | CACGCGACCGGCGCAGGTCTGAGGGCAGGTCCAGTAATAA |
| R5#43 | ACGCGCCGATCGCAGGCTAGAGCTAATGGCTCAGGTTCA |
| R5#44 | GTCGCGGGTGGCGCAACCCACAGAGAAATAGTCGGCGATA |
| R5#45 | GTCAGGCGCACCGGTCGCAGGTCCTGACAGTGAGCCCTAC |
| R5#46 | ACGCGCCGATCGCAGGCTAGAGCTAATGGCTCAGGTTTGT |
| R5#47 | GCCAGAACGCACCGATCGCAGGTTTCTGTGAGCAGGGGG |
| R5#48 | CTACATGAGGTTCTCAGATGCCCCTGTCGCAAGGGTCTG |
| R5#49 | CCTATGACGCGTCCGACGCAGGACTCATACGGGTGCTCGT |
| R5#50 | ATCGCCCCCTGACGCAAGGGTAGAGCTACTCATCCGGTCG |
| R5#51 | ATCGCCCCCTGACGCAAGGGGAGAGCTACTCATTCGGTCG |
| R5#52 | ATCGCCCCCTGACGCAAGGGTAGAGCTACTCATTCGGTAC |
| R5#53 | ATTGCCCCCTGACGCAAGGGTAGAGCTACTCATTCGGTCG |
| R5#54 | CACGCGACCGGCGCAGGTCTGAGGGCAGGTCCAGTAATAG |
| R5#55 | CACGCGACCGGCGCAGGTCTGAGGGCAGGTCCAGTAGTAT |
| R5#56 | TTCGTTGCCCATACGACGCAGTATGCGAAAGACAAATAGT |
| R5#57 | ATCGCCCCCTGACGCAAGGGCAGAGCTACTCATTCGGTCG |
| R5#58 | ACGCGCCGATCGCAGGCTAGAGCTAATGGCTCAGGTCTAT |
| R5#59 | ACGCGCCGATCGCAGGCTAGAGCTAATGGCTCAGGATTAT |
| R5#60 | ACGCGCCGATCGCAGGCTAGAGCTAATGGCTCAGGTTTCT |
| R5#61 | GTTATTGGAGTTGCCCCGTGACGCAACGGCTCCCAAACTGA |
| R5#62 | TACGCGCCGATCGCAGGCTAAGCGGGGGCGGGGGAAGAG |
| R5#63 | CAGACGCACCTGGCGCAAGGTTCTGAAGATCAGTTACGTC |
| R5#64 | ATCGCCCCCTGACGCAAGGGTAGAGCTACTCATTCGGTAG |
| R5#65 | ATCGCCCCCTGACGCACGGGTAGAGCTACTCATTCGGTCG |
| R5#66 | CAGACGCACCTGGCGCAAGGTTCTGAAGATCAGTTACGTT |
| R5#67 | ACGCGCCGATCGCAGGCTAGAGCTAATGGCTCAGGTTGAT |
| R5#68 | ACGCGCCGATCGCAGGCTAGAGCTAATGGCCCAGGTTTAT |
| R5#69 | CACGCGACCGGCGCAGGTCTGAGGGCAGGCCCAGTAATAT |
| R5#70 | CAGACGCACCTGGCGCAAGGCTCTGAAGATCAGTTACGTG |
| R5#71 | ACAGGCCCTGGGGGGCTATCACGCACCCGGCGCAGGGTTG |
| R5#72 | GTTATTGGAGTTGCCCCGTGACGCAACGGCTCCAAAGCTGA |
| R5#73 | ATCGCCCCCTGACGCAAGGGTAGAGCCACTCATTCGGTCG |
| R5#74 | CTTCCGATGAGTACGCCCCGATCGCAGGGTACTGACTAGG |
| R5#75 | GTTATTGGAGTTGCCCCGTGACGCAACGGCTCCAACACTGA |
| R5#76 | ATCGCCCCCTGACGCAAGGGTAGAGCTACCCATTCGGTCG |
| R5#77 | CACGCGACCGGCGCAGGTCTGAGGGCAGATCCAGTAATAT |
| R5#78 | ACGCGCCGATCGCAGGCTAGAGCTAATGGTTCAGGTTTAT |
| R5#79 | TTGGAACACCAATCCTGCAACCGGAGCGTTGGTGTTACAA |
| R5#80 | CAGACGCACCTGGCGCAAGGTTCTGAAGATCAGCTACGTG |
| R5#81 | GATTGCCCCTCGACGCAGAGGTCTGAGTTGGTACAAATTA |
| R5#82 | ACGCGCCGATCGCAGGCTAGAGCTACTGGCTCAGGTTTAT |
| R5#83 | ATCGCCCCCTGACGCAAGGGTAGAGCTACTCATTCGGCCG |
| R5#84 | ACGCGCCGATCGCAGGCTAGAGCTAATGGCTTAGGTTTAT |
| R5#85 | GTTATTGGAGTTGCCCCGTGACGCAACGGCTCCACAACTGA |
| R5#86 | ATCGCCCCCTGACGCAAGGGAAGAGCTACTCATTCGGTCG |
| R5#87 | ATCGCCCCCTGACGCAAGGGTAGAGCTACTCATTTGGTCG |
| R5#88 | CAGACGCACCTGGCGCAAGGGTCTGAAGATCAGTTACGTG |
| R5#89 | ATCGCCCCCTGACGCAAGGGTAGAGCTACTTATTCGGTCG |
| R5#90 | CAGACGCACCTGGCGCAAGGTTCTGGAGATCAGTTACGTG |
| R5#91 | TGATCGCCCGGACGCGTGTGACGCAACACTCCGAGACGAA |
| R5#92 | ACGCGCCGATCGCAGGCTAGAGCTAGTGGCTCAGGTTTAT |
| R5#93 | ATCGCCCCCTGACGCAAGGGTAGAGCTACTCGTTCGGTCG |
| R5#94 | ACTTGGACCCGATGCCCGTGGCGCAACGGTCGGGGCCTTC |
| R5#95 | CAGCGAGTAGGCGGGCAGTTTCGCGCCGATTGCAGGCAAAC |
| R5#96 | CACGCGACCGGCGCAGGTCTGAGGGCAGGTCCAGTACTAT |
| R5#97 | CGGTCGCGTCCGTCGCAGGACACCGTTTTGCAGAAGGTAA |
| R5#98 | CAGACGCACCTGGCGCAAGGTTCTGAAGATCAGTTACGT |
| R5#99 | GTGTAGGATCATCGCCCCCACGACGCAGTGGGTGATCGTA |
| R5#100 | GTTATTGGAGTTGCCCCGTGACGCAACGGCTCCAAAACTGG |
| R6#1 | ACGCGCCGATCGCAGGCTAGAGCTAATGGCTCAGGTTTAT |
| R6#2 | CACGCGACCGGCGCAGGTCTGAGGGCAGGTCCAGTAATAT |
| R6#3 | CAGACGCACCTGGCGCAAGGTTCTGAAGATCAGTTACGTG |
| R6#4 | ACTTGGACCCGATGCCCGTGGCGCAACGGTCGGGTCCTTC |
| R6#5 | ATCGCCCCCTGACGCAAGGGTAGAGCTACTCATTCGGTCG |
| R6#6 | GATTGCCCCTCGACGCAGAGGTCTGAGTTGGTACAAACTC |
| R6#7 | TCGCCAATGTGACGCTTCCGGCGCAGGAATCACATTCTTC |
| R6#8 | ATCTCCGATTGCCCCACGACGCAGTGGTCGGAGTTACTTT |
| R6#9 | CCGACGCACCCGGCGCAGGGTTCGGAGATCTAGTTAATTA |
| R6#10 | GAACGCACGGATCGCCGTTTCAGGGCCGGTGCAGTGCTAC |
| R6#11 | GCCAGAACGCACCGATCGCAGGTTTCTGTGAGCAGGGGG |
| R6#12 | CATGAACGCTCCGGTTGCAGGATTCATGAAGCGCGTTAA |
| R6#13 | GATTGCCCCTCGACGCAGAGGTCTGAGTTGGTACAAATTA |
| R6#14 | GATTGCCCCTCGACGCAGAGGTCTGAGTTGGTACAAATTC |
| R6#15 | TTGGAACACCAATCCTGCAACCGGAGCGTTGGTGTTACAA |
| R6#16 | CAGTGGACATTCGCGCCGATCGCAGGCAATGACTATGGAG |
| R6#17 | ACGCGCCGATCGCAGGCTATGGGCGGGCTCGGAGTCATTT |
| R6#18 | ACGCGCCGATCGCAGGCTAGAGCTAATGGCTCAGGGTTAT |
| R6#19 | GTGACGCGGTCGACGCAGACCTCACTGTGGGCAGTAACGG |
| R6#20 | TCTTCCCATTCGGAGGCCTGCGCCGGCCGCGTCCGAGGGT |
| R6#21 | GTTATTGGAGTTGCCCCGTGACGCAACGGCTCCAAAACTGA |
| R6#22 | CACGCGACCGGCGCAGGTCTGAGGGCAGGTCCAGTAATAC |
| R6#23 | ATCATTCACGCGGCCGACGCAGGCCTGAAAGATATCCTCG |
| R6#24 | GTTCGATTGCCGGGCGACGCAGCCCTCGAACTCTCAGACG |
| R6#25 | ACACACGCGGATAGGCGCATCCTGGGTAGGGGATTACGAA |
| R6#26 | ACGCGCCGATCGCAGGCTAGAGCTAATGGCTCAGGTTTAC |
| R6#27 | GACGGTGCTGCTGTACTTGGGTGTTGCGCCACACGCGCC |
| R6#28 | ACGCGCCGATCGCAGGCTAGAGCTAATGGCTCAGGTTCA |
| R6#29 | ACGCGCCGATCGCAGGCTAGAGCTAATGGCTCCGGTTTAT |
| R6#30 | AGTGAGTTGCCCCCCGACGCAGGGGCTCACGCTTGCTTA |
| R6#31 | CAGACGCACCTGGCGCAAGGTTCTGAAGATCAGTTACGCG |
| R6#32 | GTCAGGCGCACCGGTCGCAGGTCCTGACAGTGAGCCCTAC |
| R6#33 | CTTGAATCCACGCGCCGATCGCAGGCTGGATTTAGACCGC |
| R6#34 | ACGCGCCGATCGCAGGCTAGAGCTAATGGCTCAGGCTTAT |
| R6#35 | ACGCGCCGATCGCAGGCTAGAGCTAATGGCTCAGGTTTAA |
| R6#36 | TCGCGCCGATCGCAGGCAAGAGCTCTCCCGTCACGAGTGG |
| R6#37 | ATCGTTGACCAGGACGCGTTCGGCGCAGAACTCATGGTCA |
| R6#38 | ATCGCCCCCTGACGCAAGGGTAGAGCTACTCATTCGGTCA |
| R6#39 | CACGCGACCGGCGCAGGTCTGAGGGCAGGTCCAGTAATAA |
| R6#40 | AATAGCCCCCTGACGCAAGGGTTAGAGCAGTAATCATGGT |
| R6#41 | ATCGCCACCTGACGCAAGGTTAGACCAGGGACCGATGCGC |
| R6#42 | GATCCCCGTGTAACCGCTTTTCGCCGCAGAAATTACACTT |
| R6#43 | CAGACGCACCTGGCGCAAGGTTCTGAAGATCAGTTACGTA |
| R6#44 | ACGCGCCGATCGCAGGCTAGAGCTAATGGCTCAGGTTTGT |
| R6#45 | GTCGCGGGTGGCGCAACCCACAGAGAAATAGTCGGCGATA |
| R6#46 | ACGCGCCGATCGCAGGCTAGAGCTAATGGCTCAGGTCTAT |
| R6#47 | CACGCGACCGGCGCAGGTCTGAGGGCAGGTCCAGTAGTAT |
| R6#48 | AATTGCTCCCCTGACGCAAGGGGTTAGACTTACGATTCC |
| R6#49 | CACGCGACCGGCGCAGGTCTGAGGGCAGGTCCAGTAATAG |
| R6#50 | ACGCGCCGATCGCAGGCTAGAGCTAATGGTTCAGGTTTAT |
| R6#51 | ACGCGCCGATCGCAGGCTAGAGCTAATGGCTCAGGTTTCT |
| R6#52 | GACTCCGGGACATGCCCATGTCGCAATGGTGGCCCGGATT |
| R6#53 | CCACGATTGCTCCCCAACGCCGGGGTCGGGGAAACGCGTC |
| R6#54 | TGATCGTCCGGACGCGTGTGGCGCAACACTCCGAGACGAA |
| R6#55 | CACGCGACCGGCGCAGGTCTGAGGGCAGGCCCAGTAATAT |
| R6#56 | CACGCGACCGGCGCAGGTCTGAGGGCAGATCCAGTAATAT |
| R6#57 | ACGCGCCGATCGCAGGCTAGAGCTAATGGCTTAGGTTTAT |
| R6#58 | ACGCGCCGATCGCAGGCTAGAGCTAATGGCCCAGGTTTAT |
| R6#59 | ACGCGCCGATCGCAGGCTAGAGCTAATGGCTCAGGATTAT |
| R6#60 | CAGACGCACCTGGCGCAAGGTTCTGAAGATCAGTTACGTC |
| R6#61 | GATTGCTCCCCGACGCAGGGGTCAGTGAGTATCTGGATAA |
| R6#62 | CAGACGCACCTGGCGCAAGGTTCTGAGGATCAGTTACGTG |
| R6#63 | CAGACGCACCTGGCGCAAGGTTCTGAAGATCAGTTACGTT |
| R6#64 | ACGCGCCGATCGCAGGCTAGAGCTAATGGCTCAGGTTGAT |
| R6#65 | TCGATGAGCCGAGGCAGGGAACTAGGTACTCGAGCTTCTG |
| R6#66 | TACGCGCCGATCGCAGGCTAAGCGGGGGCGGGGGAAGAG |
| R6#67 | CACGCGACCGGCGCAGGTCTGAGGGCAGGGCCAGTAATAT |
| R6#68 | ACTTGGACCCGATGCCCGTGGCGCAACGGTCGGGGCCTTC |
| R6#69 | ACGCGCCGATCGCAGGCTAGAGCTAGTGGCTCAGGTTTAT |
| R6#70 | CACGCGACCGGCGCAGGTCTGAGGGCAGGTCCAGTACTAT |
| R6#71 | GCTCCGGACATCGCTACCCGACGCAGGGTTGTCCTTGGTTA |
| R6#72 | TTCGTTGCCCATACGACGCAGTATGCGAAAGACAAATAGT |
| R6#73 | ATCGCCCCCTGACGCAAGGGTAGAGCTACTCATTCGGTCT |
| R6#74 | TCGATGAGCCGAGGCAGGGAACTAGGTACTCGAGCTTCAG |
| R6#75 | CCTATGACGCGTCCGACGCAGGACTCATACGGGTGCTCGT |
| R6#76 | ATCAGCAGGGTGGACTTCGATGTCGTTGTGCAAACCGGTC |
| R6#77 | CACGCTACTGTCGCAAGTATGAGAGCGGATCAGCGTAGT |
| R6#78 | ACGCGCCGATCGCAGGCTAGAGCTAACGGCTCAGGTTTAT |
| R6#79 | GTGTAGGATCATCGCCCCCACGACGCAGTGGGTGATCGTA |
| R6#80 | GATTGCCCCTCGACGCAGAGGTCTGAGTTGGTACAAACTA |
| R6#81 | ACGCGCCGATCGCAGGCTAGAGCTAATGACTCAGGTTTAT |
| R6#82 | ACGCGCCGATCGCAGGCTAGAGCTAATGGCTCAGGTTTAG |
| R6#83 | GCTTGACCGAACGCACGGATCGCCGTTTCGGAGATAATTG |
| R6#84 | AGTGAGTTGCTCCCCGACGCAGGGGCTCACGCTTGCTTA |
| R6#85 | GAACGCACGGATCGCCGTTTCAGGGCCGGTGCAGTGCTAT |
| R6#86 | CACGCGACCGGCGCAGGTCTGAGGGTAGGTCCAGTAATAT |
| R6#87 | CACGCGACCGGCGCAGGGCTGAGGGCAGGTCCAGTAATAT |
| R6#88 | TCGATGAGCCGAGGCAGGGAACTAGGTACTCGAGCTTCTA |
| R6#89 | CCGACGCACCCGGCGCAGGGTTCGGAGATCTGGTTAATTA |
| R6#90 | ACGCGCCGATCGCAGGCTAGAGCTACTGGCTCAGGTTTAT |
| R6#91 | ATCTCCGATTGCCCCACGACGCAGTGGTCGGAGTTACTTC |
| R6#92 | ACGCGCCGATCGCAGGCTAGAGCTAATGGCTCAGGTATAT |
| R6#93 | ATTCCTGCTCATTTGCGCTTCAGCCTGCAACCGGCGCGTG |
| R6#94 | CAGACGCACCTGGCGCAAGGTTCTGAAGATCAGCTACGTG |
| R6#95 | CACGCGACCGGCGCAGGTCTGAGGGCAGGTCCAGTCATAT |
| R6#96 | ACCGCTGTATGACGCAATACTAGAGCCACGATGCCTTCAG |
| R6#97 | CACGCGACCGGCGCAGGTCTGAGGGCAGGTCCAGTAATGT |
| R6#98 | CACGCGACCGGCGCAGGTCTGAGGGCGGGTCCAGTAATAT |
| R6#99 | ACGCGCCGATCGCAGGCTAAGCGGGGGCGGGGGAAGAG |
| R6#100 | CACGCGACCGGCGCAGGTCTGAGGGCAGGTCCCGTAATAT |
| R7#1 | CACGCGACCGGCGCAGGTCTGAGGGCAGGTCCAGTAATAT |
| R7#2 | ACGCGCCGATCGCAGGCTAGAGCTAATGGCTCAGGTTTAT |
| R7#3 | GATTGCCCCTCGACGCAGAGGTCTGAGTTGGTACAAACTC |
| R7#4 | ACTTGGACCCGATGCCCGTGGCGCAACGGTCGGGTCCTTC |
| R7#5 | CAGACGCACCTGGCGCAAGGTTCTGAAGATCAGTTACGTG |
| R7#6 | ATCGCCCCCTGACGCAAGGGTAGAGCTACTCATTCGGTCG |
| R7#7 | TCTTCCCATTCGGAGGCCTGCGCCGGCCGCGTCCGAGGGT |
| R7#8 | CCGACGCACCCGGCGCAGGGTTCGGAGATCTAGTTAATTA |
| R7#9 | ATCTCCGATTGCCCCACGACGCAGTGGTCGGAGTTACTTT |
| R7#10 | GATTGCCCCTCGACGCAGAGGTCTGAGTTGGTACAAATTA |
| R7#11 | GATTGCCCCTCGACGCAGAGGTCTGAGTTGGTACAAATTC |
| R7#12 | TCGCCAATGTGACGCTTCCGGCGCAGGAATCACATTCTTC |
| R7#13 | GTTCGATTGCCGGGCGACGCAGCCCTCGAACTCTCAGACG |
| R7#14 | GCCAGAACGCACCGATCGCAGGTTTCTGTGAGCAGGGGG |
| R7#15 | GATCCCCGTGTAACCGCTTTTCGCCGCAGAAATTACACTT |
| R7#16 | GTGACGCGGTCGACGCAGACCTCACTGTGGGCAGTAACGG |
| R7#17 | CATGAACGCTCCGGTTGCAGGATTCATGAAGCGCGTTAA |
| R7#18 | TTGGAACACCAATCCTGCAACCGGAGCGTTGGTGTTACAA |
| R7#19 | AATAGCCCCCTGACGCAAGGGTTAGAGCAGTAATCATGGT |
| R7#20 | GACTCCGGGACATGCCCATGTCGCAATGGTGGCCCGGATT |
| R7#21 | GTTATTGGAGTTGCCCCGTGACGCAACGGCTCCAAAACTGA |
| R7#22 | GAACGCACGGATCGCCGTTTCAGGGCCGGTGCAGTGCTAC |
| R7#23 | ACACACGCGGATAGGCGCATCCTGGGTAGGGGATTACGAA |
| R7#24 | TGATCGTCCGGACGCGTGTGGCGCAACACTCCGAGACGAA |
| R7#25 | ATCATTCACGCGGCCGACGCAGGCCTGAAAGATATCCTCG |
| R7#26 | GGCCTAGAGATTGCCGGTCGACGCAGACCTCTCTTGGATG |
| R7#27 | ATCGCCCCCTGACGCAAGGGTAGAGCTACTCATTCGGTCA |
| R7#28 | ACGCGCCGATCGCAGGCTATGGGCGGGCTCGGAGTCATTT |
| R7#29 | CACGCGACCGGCGCAGGTCTGAGGGCAGGTCCAGTAATAC |
| R7#30 | GATTGCCCCTCGACGCAGAGGTCTGAGTTGGTACGAACTC |
| R7#31 | GATTGCCCCTCGACGCAGAGGTCTGAGTTGGTACAAACTA |
| R7#32 | AGTGAGTTGCCCCCCGACGCAGGGGCTCACGCTTGCTTA |
| R7#33 | ACGCGCCGATCGCAGGCTAGAGCTAATGGCTCAGGTTCA |
| R7#34 | CAGACGCACCTGGCGCAAGGTTCTGAAGATCAGTTACGTA |
| R7#35 | CAGTGGACATTCGCGCCGATCGCAGGCAATGACTATGGAG |
| R7#36 | ATCGTTGACCAGGACGCGTTCGGCGCAGAACTCATGGTCA |
| R7#37 | GATTGCCCCTCGACGCAGAGGTCTGAGTTGGTACAAGCTC |
| R7#38 | ACGCGCCGATCGCAGGCTAGAGCTAATGGCTCAGGTTTAC |
| R7#39 | GTGTAGGATCATCGCCCCCACGACGCAGTGGGTGATCGTA |
| R7#40 | CAGACGCACCTGGCGCAAGGTTCTGAAGATCAGTTACGCG |
| R7#41 | ACGCGCCGATCGCAGGCTAGAGCTAATGGCTCAGGGTTAT |
| R7#42 | ATCGCCACCTGACGCAAGGTTAGACCAGGGACCGATGCGC |
| R7#43 | ACCGCTGTATGACGCAATACTAGAGCCACGATGCCTTCAG |
| R7#44 | GCTCCGGACATCGCTACCCGACGCAGGGTTGTCCTTGGTTA |
| R7#45 | ACGCGCCGATCGCAGGCTAGAGCTAATGGTTCAGGTTTAT |
| R7#46 | ACGCGCCGATCGCAGGCTAGAGCTAATGGCTCAGGTTTAA |
| R7#47 | CACGCGACCGGCGCAGGTCTGAGGGCAGGTCCAGTAATAA |
| R7#48 | GATTGCCCCTCGACGCAGAGGTCTGAGTTGGTACAAACTT |
| R7#49 | CACGCGACCGGCGCAGGTCTGAGGGCAGATCCAGTAATAT |
| R7#50 | CACGCGACCGGCGCAGGTCTGAGGGCAGGTCCAGTAGTAT |
| R7#51 | ACGCGCCGATCGCAGGCTAGAGCTAATGGCTCAGGTCTAT |
| R7#52 | ACGCGCCGATCGCAGGCTAGAGCTAATGGCTTAGGTTTAT |
| R7#53 | CCGACGCACCCGGCGCAGGGTTCGGAGATCTGGTTAATTA |
| R7#54 | GATTGCCCCTCGACGCAGAGGTCTGAGCTGGTACAAACTC |
| R7#55 | CAGACGCACCTGGCGCAAGGTTCTGAAGATCAGTTACGTC |
| R7#56 | ACGCGCCGATCGCAGGCTAGAGCTAATGGCTCAGGTTTCT |
| R7#57 | GATTGCTCCCCGACGCAGGGGTCAGTGAGTATCTGGATAA |
| R7#58 | ATCTCCGATTGCCCCACGACGCAGTGGTCGGAGTTACTTC |
| R7#59 | ACAGGCCCTGGGGGGCTATCACGCACCCGGCGCAGGGTTG |
| R7#60 | ACGCGCCGATCGCAGGCTAGAGCTAATGGCTCAGGTTTGT |
| R7#61 | CAGACGCACCTGGCGCAAGGTTCTGAAGATCAGTTACGTT |
| R7#62 | AGTGAGTTGCTCCCCGACGCAGGGGCTCACGCTTGCTTA |
| R7#63 | AATTGCTCCCCTGACGCAAGGGGTTAGACTTACGATTCC |
| R7#64 | GTCGCGGGTGGCGCAACCCACAGAGAAATAGTCGGCGATA |
| R7#65 | ATCGCCCCCTGACGCAAGGGTAGAGCTACTCATTCGGTCT |
| R7#66 | CACGCGACCGGCGCAGGTCTGAGGGCAGGTCCAGTAATAG |
| R7#67 | TCGATGAGCCGAGGCAGGGAACTAGGTACTCGAGCTTCTA |
| R7#68 | TAGGGCGATTAACGCAACCGGCGCAGGTTTTAAACGCGCT |
| R7#69 | ACGCGCCGATCGCAGGCTAGAGCTAATGGCTCCGGTTTAT |
| R7#70 | ACGCGCCGATCGCAGGCTAGAGCTAATGGCCCAGGTTTAT |
| R7#71 | CACGCGACCGGCGCAGGTCTGAGGGCAGGCCCAGTAATAT |
| R7#72 | TCGATGAGCCGAGGCAGGGAACTAGGTACTCGAGCTTCTG |
| R7#73 | GATTGCCCCTCGACGCAGAGGTCTGAGTTGGTACACACTC |
| R7#74 | ATCTCCGATTGCCCCACGACGCAGTGGTCGGAGTTACTAT |
| R7#75 | GATTGCCCCTCGACGCAGAGGTCTGAGTTGGTACAGACTC |
| R7#76 | CACCGCTATCCGCCGCAGGATTGGGAGCATAGATACACGC |
| R7#77 | ACGCGCCGATCGCAGGCTAGAGCTAATGGCTCAGGCTTAT |
| R7#78 | GTCAGGCGCACCGGTCGCAGGTCCTGACAGTGAGCCCTAC |
| R7#79 | CAGACGCACCTGGCGCAAGGTTCTGAGGATCAGTTACGTG |
| R7#80 | ACGCGCCGATCGCAGGCTAAGCGGGGGCGGGGGAAGAG |
| R7#81 | GATTGCCCCTCGACGCAGAGGTCTGAGTTGGTACAAACCC |
| R7#82 | CCTATGACGCGTCCGACGCAGGACTCATACGGGTGCTCGT |
| R7#83 | TTCGTTGCCCATACGACGCAGTATGCGAAAGACAAATAGT |
| R7#84 | ATTGCCCCCTGACGCAAGGGTAGAGCTACTCATTCGGTCG |
| R7#85 | ATTCCTGCTCATTTGCGCTTCAGCCTGCAACCGGCGCGTG |
| R7#86 | ATCGCCCCCTGACGCAAGGGTAGAGCTACTCATTCGGTCC |
| R7#87 | ATCAGCAGGGTGGACTTCGATGTCGTTGTGCAAACCGGTC |
| R7#88 | GATTGTCCGTCTTCGAGGATTGCGTCATCCGCGTCGAAGA |
| R7#89 | CCACGATTGCTCCCCAACGCCGGGGTCGGGGAAACGCGTC |
| R7#90 | ACTTGGACCCGATGCCCGTGGCGCAACGGTCGGGGCCTTC |
| R7#91 | TCGCGCCGATCGCAGGCAAGAGCTCTCCCGTCACGAGTGG |
| R7#92 | CTACATGAGGTTCTCAGATGCCCCTGTCGCAAGGGTCTG |
| R7#93 | TCGATGAGCCGAGGCAGGGAACTAGGTACTCGAGCTTCAG |
| R7#94 | CTTGAATCCACGCGCCGATCGCAGGCTGGATTTAGACCGC |
| R7#95 | CAGCGAGTAGGCGGGCAGTTTCGCGCCGATTGCAGGCAAAC |
| R7#96 | CGCCATGTCTCACACGCGGCCGGCGCAGGCCTGTGAGAT |
| R7#97 | GGTGGCTCGAACCTTGCGTCAGGTGCATCGGCCACATTGG |
| R7#98 | ACCTCCGATTGCCCCACGACGCAGTGGTCGGAGTTACTTT |
| R7#99 | CTGGCGTATTCGGACCGCTGGGTGACGCAACCCTCCTAAT |
| R7#100 | GGCTTAGAGATTGCCGGTCGACGCAGACCTCTCTTGGATG |
| R8#1 | ACGCGCCGATCGCAGGCTAGAGCTAATGGCTCAGGTTTAT |
| R8#2 | TCTTCCCATTCGGAGGCCTGCGCCGGCCGCGTCCGAGGGT |
| R8#3 | CACGCGACCGGCGCAGGTCTGAGGGCAGGTCCAGTAATAT |
| R8#4 | ACTTGGACCCGATGCCCGTGGCGCAACGGTCGGGTCCTTC |
| R8#5 | CAGACGCACCTGGCGCAAGGTTCTGAAGATCAGTTACGTG |
| R8#6 | GATTGCCCCTCGACGCAGAGGTCTGAGTTGGTACAAATTA |
| R8#7 | GATTGCCCCTCGACGCAGAGGTCTGAGTTGGTACAAACTC |
| R8#8 | CCGACGCACCCGGCGCAGGGTTCGGAGATCTAGTTAATTA |
| R8#9 | GATTGCCCCTCGACGCAGAGGTCTGAGTTGGTACAAATTC |
| R8#10 | ATCGCCCCCTGACGCAAGGGTAGAGCTACTCATTCGGTCG |
| R8#11 | GCCAGAACGCACCGATCGCAGGTTTCTGTGAGCAGGGGG |
| R8#12 | ATCTCCGATTGCCCCACGACGCAGTGGTCGGAGTTACTTT |
| R8#13 | GATCCCCGTGTAACCGCTTTTCGCCGCAGAAATTACACTT |
| R8#14 | GTGACGCGGTCGACGCAGACCTCACTGTGGGCAGTAACGG |
| R8#15 | GACTCCGGGACATGCCCATGTCGCAATGGTGGCCCGGATT |
| R8#16 | GGCCTAGAGATTGCCGGTCGACGCAGACCTCTCTTGGATG |
| R8#17 | GTTCGATTGCCGGGCGACGCAGCCCTCGAACTCTCAGACG |
| R8#18 | TGATCGTCCGGACGCGTGTGGCGCAACACTCCGAGACGAA |
| R8#19 | CATGAACGCTCCGGTTGCAGGATTCATGAAGCGCGTTAA |
| R8#20 | TTGGAACACCAATCCTGCAACCGGAGCGTTGGTGTTACAA |
| R8#21 | GAACGCACGGATCGCCGTTTCAGGGCCGGTGCAGTGCTAC |
| R8#22 | ACCGCTGTATGACGCAATACTAGAGCCACGATGCCTTCAG |
| R8#23 | AATAGCCCCCTGACGCAAGGGTTAGAGCAGTAATCATGGT |
| R8#24 | GTGTAGGATCATCGCCCCCACGACGCAGTGGGTGATCGTA |
| R8#25 | TCGCCAATGTGACGCTTCCGGCGCAGGAATCACATTCTTC |
| R8#26 | ACGCGCCGATCGCAGGCTATGGGCGGGCTCGGAGTCATTT |
| R8#27 | GTTATTGGAGTTGCCCCGTGACGCAACGGCTCCAAAACTGA |
| R8#28 | GATTGCCCCTCGACGCAGAGGTCTGAGTTGGTACGAACTC |
| R8#29 | ACACACGCGGATAGGCGCATCCTGGGTAGGGGATTACGAA |
| R8#30 | GATTGCCCCTCGACGCAGAGGTCTGAGTTGGTACAAACTA |
| R8#31 | CAGTGGACATTCGCGCCGATCGCAGGCAATGACTATGGAG |
| R8#32 | GATTGCCCCTCGACGCAGAGGTCTGAGTTGGTACAAGCTC |
| R8#33 | ATCGCCCCCTGACGCAAGGGTAGAGCTACTCATTCGGTCA |
| R8#34 | ACGCGCCGATCGCAGGCTAGAGCTAATGGCTCAGGTTCA |
| R8#35 | ATCGTTGACCAGGACGCGTTCGGCGCAGAACTCATGGTCA |
| R8#36 | ATCATTCACGCGGCCGACGCAGGCCTGAAAGATATCCTCG |
| R8#37 | GCTCCGGACATCGCTACCCGACGCAGGGTTGTCCTTGGTTA |
| R8#38 | AGTGAGTTGCCCCCCGACGCAGGGGCTCACGCTTGCTTA |
| R8#39 | CACGCGACCGGCGCAGGTCTGAGGGCAGGTCCAGTAATAC |
| R8#40 | CAGACGCACCTGGCGCAAGGTTCTGAAGATCAGTTACGTA |
| R8#41 | ACGCGCCGATCGCAGGCTAGAGCTAATGGTTCAGGTTTAT |
| R8#42 | ATCGCCACCTGACGCAAGGTTAGACCAGGGACCGATGCGC |
| R8#43 | CCGACGCACCCGGCGCAGGGTTCGGAGATCTGGTTAATTA |
| R8#44 | ACAGGCCCTGGGGGGCTATCACGCACCCGGCGCAGGGTTG |
| R8#45 | GATTGCCCCTCGACGCAGAGGTCTGAGTTGGTACAAACTT |
| R8#46 | AGTGAGTTGCTCCCCGACGCAGGGGCTCACGCTTGCTTA |
| R8#47 | ACGCGCCGATCGCAGGCTAGAGCTAATGGCTCAGGTTTAC |
| R8#48 | GATTGCCCCTCGACGCAGAGGTCTGAGCTGGTACAAACTC |
| R8#49 | ACGCGCCGATCGCAGGCTAAGCGGGGGCGGGGGAAGAG |
| R8#50 | CCTATGACGCGTCCGACGCAGGACTCATACGGGTGCTCGT |
| R8#51 | ACGCGCCGATCGCAGGCTAGAGCTAATGGCTCAGGGTTAT |
| R8#52 | ACGCGCCGATCGCAGGCTAGAGCTAATGGCTCAGGTCTAT |
| R8#53 | CAGACGCACCTGGCGCAAGGTTCTGAAGATCAGTTACGCG |
| R8#54 | TAGGGCGATTAACGCAACCGGCGCAGGTTTTAAACGCGCT |
| R8#55 | ACGCGCCGATCGCAGGCTAGAGCTAATGGCTTAGGTTTAT |
| R8#56 | ACGCGCCGATCGCAGGCTAGAGCTAATGGCTCAGGTTTCT |
| R8#57 | ACGCGCCGATCGCAGGCTAGAGCTAATGGCTCAGGTTTAA |
| R8#58 | CGCCATGTCTCACACGCGGCCGGCGCAGGCCTGTGAGAT |
| R8#59 | CACGCGACCGGCGCAGGTCTGAGGGCAGATCCAGTAATAT |
| R8#60 | ATTGCCCCCTGACGCAAGGGTAGAGCTACTCATTCGGTCG |
| R8#61 | GGCTTAGAGATTGCCGGTCGACGCAGACCTCTCTTGGATG |
| R8#62 | CGGGTTCACCGTTGCGTCACGGGGCAATTCACCACTCGCC |
| R8#63 | CAGCGAGTAGGCGGGCAGTTTCGCGCCGATTGCAGGCAAAC |
| R8#64 | TCGCGCCGATCGCAGGCAAGAGCTCTCCCGTCACGAGTGG |
| R8#65 | ATCTCCGATTGCCCCACGACGCAGTGGTCGGAGTTACTAT |
| R8#66 | GATTGCCCCTCGACGCAGAGGTCTGAGTTGGTACAAACCC |
| R8#67 | ACAGATGTTGCCCTCCGACGCAGGAGCATCTAAACTGCAC |
| R8#68 | ATCTCCGATTGCCCCACGACGCAGTGGTCGGAGTTACTTC |
| R8#69 | GTCGCGGGTGGCGCAACCCACAGAGAAATAGTCGGCGATA |
| R8#70 | GATTGCTCCCCGACGCAGGGGTCAGTGAGTATCGGATAG |
| R8#71 | CACCGCTATCCGCCGCAGGATTGGGAGCATAGATACACGC |
| R8#72 | ACTTTAGGGCTAACCTGCGATCGGTGCGTACCCCATGCTC |
| R8#73 | ATCGCCCCCTGACGCAAGGGTAGAGCTACTCATTCGGTCT |
| R8#74 | GATTGCTCCCCGACGCAGGGGTCAGTGAGTATCTGGATAA |
| R8#75 | ACGCGCCGATCGCAGGCTAGAGCTAATGGCCCAGGTTTAT |
| R8#76 | CTGGCGTATTCGGACCGCTGGGTGACGCAACCCTCCTAAT |
| R8#77 | GCTTGGAGCACGCCGCTGGTTGACGCAAACCCGATGCTTA |
| R8#78 | ACGCGCCGATCGCAGGCTAGAGCTAATGGCTCAGGTTTGT |
| R8#79 | CAGACGCACCTGGCGCAAGGTTCTGAAGATCAGTTACGTT |
| R8#80 | CTACGGACACGGACGCTCGTGTTGCTCGATCCGTGTGGTT |
| R8#81 | CACGCGACCGGCGCAGGTCTGAGGGCAGGTCCAGTAGTAT |
| R8#82 | ACCAGAACGCACCGATCGCAGGTTTCTGTGAGCAGGGGG |
| R8#83 | TCGATGAGCCGAGGCAGGGAACTAGGTACGCAAGCTCAC |
| R8#84 | CACGCGACCGGCGCAGGTCTGAGGGCAGGTCCAGTAATAA |
| R8#85 | CACGCGACCGGCGCAGGTCTGAGGGCAGGCCCAGTAATAT |
| R8#86 | GATTGCCCCTCGACGCAGAGGTCTGAGTTGGTACAGACTC |
| R8#87 | ACGCGCCGATCGCAGGCTAGAGCTAATGGCTCCGGTTTAT |
| R8#88 | ATGGCAGACGCACCCGGCGCAGGGTTCTGCTATCGACCTA |
| R8#89 | TTGTGTGCCCCCTGATTGCCCCTCGACGCAGAGGTCTGGG |
| R8#90 | TCTTCCCACTCGGAGGCCTGCGCCGGCCGCGTCCGAGGGT |
| R8#91 | TTCGGTGTACTCCAGATTGCCGCTCGACGCAGAGCTCTGG |
| R8#92 | ACCTCCGATTGCCCCACGACGCAGTGGTCGGAGTTACTTT |
| R8#93 | ACGCGCCGATCGCAGGCTAGAGCTAATGGCTCAGGCTTAT |
| R8#94 | CACGCGACCGGCGCAGGTCTGAGGGCAGGTCCAGTAATAG |
| R8#95 | ACTTCCCATTCGGAGGCCTGCGCCGGCCGCGTCCGAGGGT |
| R8#96 | TCTTCCCATTCGGAGGCCTGCGCCGGCCGCGTCCGCGGGT |
| R8#97 | GAACGCACGGATCGCCGTTTCAGGGCCGGTGCAGTGCTAT |
| R8#98 | CAGACGCACCTGGCGCAAGGTTCTGAGGATCAGTTACGTG |
| R8#99 | ATCAGCAGGGTGGACTTCGATGTCGTTGTGCAAACCGGTC |
| R8#100 | ACGCTTGCATCCGCCGCTAAGTGACGCAACTTCGGATCGT |
| R9#1 | TCTTCCCATTCGGAGGCCTGCGCCGGCCGCGTCCGAGGGT |
| R9#2 | CACGCGACCGGCGCAGGTCTGAGGGCAGGTCCAGTAATAT |
| R9#3 | ACGCGCCGATCGCAGGCTAGAGCTAATGGCTCAGGTTTAT |
| R9#4 | GATTGCCCCTCGACGCAGAGGTCTGAGTTGGTACAAATTA |
| R9#5 | GATTGCCCCTCGACGCAGAGGTCTGAGTTGGTACAAACTC |
| R9#6 | GATTGCCCCTCGACGCAGAGGTCTGAGTTGGTACAAATTC |
| R9#7 | GATCCCCGTGTAACCGCTTTTCGCCGCAGAAATTACACTT |
| R9#8 | CAGACGCACCTGGCGCAAGGTTCTGAAGATCAGTTACGTG |
| R9#9 | CCGACGCACCCGGCGCAGGGTTCGGAGATCTAGTTAATTA |
| R9#10 | ACTTGGACCCGATGCCCGTGGCGCAACGGTCGGGTCCTTC |
| R9#11 | TGATCGTCCGGACGCGTGTGGCGCAACACTCCGAGACGAA |
| R9#12 | ATCGCCCCCTGACGCAAGGGTAGAGCTACTCATTCGGTCG |
| R9#13 | GATTGCCCCTCGACGCAGAGGTCTGAGTTGGTACGAACTC |
| R9#14 | ATCTCCGATTGCCCCACGACGCAGTGGTCGGAGTTACTTT |
| R9#15 | GGCCTAGAGATTGCCGGTCGACGCAGACCTCTCTTGGATG |
| R9#16 | GTGACGCGGTCGACGCAGACCTCACTGTGGGCAGTAACGG |
| R9#17 | GACTCCGGGACATGCCCATGTCGCAATGGTGGCCCGGATT |
| R9#18 | CATGAACGCTCCGGTTGCAGGATTCATGAAGCGCGTTAA |
| R9#19 | AATAGCCCCCTGACGCAAGGGTTAGAGCAGTAATCATGGT |
| R9#20 | TTGGAACACCAATCCTGCAACCGGAGCGTTGGTGTTACAA |
| R9#21 | GTTCGATTGCCGGGCGACGCAGCCCTCGAACTCTCAGACG |
| R9#22 | ACCGCTGTATGACGCAATACTAGAGCCACGATGCCTTCAG |
| R9#23 | GATTGCCCCTCGACGCAGAGGTCTGAGTTGGTACAAGCTC |
| R9#24 | GATTGCCCCTCGACGCAGAGGTCTGAGCTGGTACAAACTC |
| R9#25 | TCGCCAATGTGACGCTTCCGGCGCAGGAATCACATTCTTC |
| R9#26 | GATTGCCCCTCGACGCAGAGGTCTGAGTTGGTACAAACTA |
| R9#27 | ACGCGCCGATCGCAGGCTAGAGCTAATGGCTCAGGTTCA |
| R9#28 | GAACGCACGGATCGCCGTTTCAGGGCCGGTGCAGTGCTAC |
| R9#29 | CGGGTTCACCGTTGCGTCACGGGGCAATTCACCACTCGCC |
| R9#30 | GCCAGAACGCACCGATCGCAGGTTTCTGTGAGCAGGGGG |
| R9#31 | ACGCGCCGATCGCAGGCTAGAGCTAATGGTTCAGGTTTAT |
| R9#32 | ACGCGCCGATCGCAGGCTAAGCGGGGGCGGGGGAAGAG |
| R9#33 | TCGATGAGCCGAGGCAGGGAACTAGGTACGCAAGCTCAC |
| R9#34 | ACGCGCCGATCGCAGGCTATGGGCGGGCTCGGAGTCATTT |
| R9#35 | CAGACGCACCTGGCGCAAGGTTCTGAAGATCAGTTACGTA |
| R9#36 | CACGCGACCGGCGCAGGTCTGAGGGCAGGTCCAGTAATAC |
| R9#37 | GATTGCCCCTCGACGCAGAGGTCTGAGTTGGTACAAACTT |
| R9#38 | CCGACGCACCCGGCGCAGGGTTCGGAGATCTGGTTAATTA |
| R9#39 | CACAACGGGATTGCCCTCTGACGCAAGAGTCCCGTTCTG |
| R9#40 | ACGCGCCGATCGCAGGCTAGAGCTAATGGCTTAGGTTTAT |
| R9#41 | ATCGCCCCCTGACGCAAGGGTAGAGCTACTCATTCGGTCA |
| R9#42 | CACCGCTATCCGCCGCAGGATTGGGAGCATAGATACACGC |
| R9#43 | GATTGCCCCTCGACGCAGAGGTCAGAGTTGGTACAAATTA |
| R9#44 | ACGCGCCGATCGCAGGCTAGAGCTAATGGCTCAGGTTTAC |
| R9#45 | ACTTTAGGGCTAACCTGCGATCGGTGCGTACCCCATGCTC |
| R9#46 | CACGCGACCGGCGCAGGTCTGAGGGCAGATCCAGTAATAT |
| R9#47 | CAGACGCACCTGGCGCAAGGTTCTGAAGATCAGTTACGCG |
| R9#48 | ACTTCCCATTCGGAGGCCTGCGCCGGCCGCGTCCGAGGGT |
| R9#49 | ACGCGCCGATCGCAGGCTAGAGCTAATGGCTCAGGTCTAT |
| R9#50 | GATTGCCCCTCGACGCAGAGGTCTGAGTTGGTACAGACTC |
| R9#51 | GTTATTGGAGTTGCCCCGTGACGCAACGGCTCCAAAACTGA |
| R9#52 | GATTGCCCCTCGACGCAGAGGTCTGAGTTGGTACAAACCC |
| R9#53 | ACACACGCGGATAGGCGCATCCTGGGTAGGGGATTACGAA |
| R9#54 | ATCATTCACGCGGCCGACGCAGGCCTGAAAGATATCCTCG |
| R9#55 | ACGCGCCGATCGCAGGCTAGAGCTAATGGCTCAGGTTTCT |
| R9#56 | TCTTCCCACTCGGAGGCCTGCGCCGGCCGCGTCCGAGGGT |
| R9#57 | ATCGTTGACCAGGACGCGTTCGGCGCAGAACTCATGGTCA |
| R9#58 | ATCGCCACCTGACGCAAGGTTAGACCAGGGACCGATGCGC |
| R9#59 | ACGCGCCGATCGCAGGCTAGAGCTAATGGCTCAGGGTTAT |
| R9#60 | CGATGGCGCGTACGCATGTGGCGCAACATTACGCGCCCAT |
| R9#61 | ACGCGCCGATCGCAGGCTAGAGCTAATGGCCCAGGTTTAT |
| R9#62 | AGTGAGTTGCTCCCCGACGCAGGGGCTCACGCTTGCTTA |
| R9#63 | CACGCGACCGGCGCAGGTCTGAGGGCAGGTCCAGTAGTAT |
| R9#64 | ACAGATGTTGCCCTCCGACGCAGGAGCATCTAAACTGCAC |
| R9#65 | CAGACGCACCTGGCGCAAGGTTCTGAAGATCAGTTACGTT |
| R9#66 | GATTGCCCCTCGACGCAGAGGTCTGAGTAGGTACAAACTC |
| R9#67 | GTGTAGGATCATCGCCCCCACGACGCAGTGGGTGATCGTA |
| R9#68 | GACGGTGCTGCCGTACTTGGGTGTTGCGCCACACGCGCC |
| R9#69 | CACTATTCGAAACGCATCCGGCGCAGGATTTTCGAATCTC |
| R9#70 | CAGTGGACATTCGCGCCGATCGCAGGCAATGACTATGGAG |
| R9#71 | ACGCGCCGATCGCAGGCTAGAGCTAATGACTCAGGTTTAT |
| R9#72 | AGTGAGTTGCCCCCCGACGCAGGGGCTCACGCTTGCTTA |
| R9#73 | GGTGGCTCGAACCTTGCGTCAGGTGCATCGGCCACATTGG |
| R9#74 | ACGCGCCGATCGCAGGCTAGAGCTAATGGCTCAGGTTTAA |
| R9#75 | ATCTCCGATTGCCCCACGACGCAGTGGTCGGAGTTACTAT |
| R9#76 | CACGCGACCGGCGCAGGTCTGAGGGCAGGTCCAGTAATAA |
| R9#77 | CACGCGACCGGCGCAGGTCTGAGGGCAGGCCCAGTAATAT |
| R9#78 | TAGGGCGATTAACGCAACCGGCGCAGGTTTTAAACGCGCT |
| R9#79 | ACAGGCCCTGGGGGGCTATCACGCACCCGGCGCAGGGTTG |
| R9#80 | CAGACGCACCTGGCGCAAGGTTCTGAAGATCAGTTACGTC |
| R9#81 | TCTTCCCATTCGGAGGCCTGCGCCGGCCGCGTCCGCGGGT |
| R9#82 | ACGCGCCGATCGCAGGCTAGAGCTAATGGCTCAGGTTTGT |
| R9#83 | GATTGCCCCTCGACGCAGAGGTCAGAGTTGGTACAAACTC |
| R9#84 | GATTGCCCCTCGACGCAGAGGTCTGAGTTGGTACAAATAT |
| R9#85 | CGGGTTCTCCGTTGCGTCACGGGGCAATTCACCACTCGCC |
| R9#86 | CACGCGACCGGCGCAGGTCTGAGGGCAGGTCCAGTAATAG |
| R9#87 | ACGCGCCGATCGCAGGCTAGAGCTAACGGCTCAGGTTTAT |
| R9#88 | GATTGCCCCTCGACGCAGAGGTCTGAGTTGGTACAGATTA |
| R9#89 | GCTCCGGACATCGCTACCCGACGCAGGGTTGTCCTTGGTTA |
| R9#90 | ATCGCCCCCTGACGCAAGGGTAGAGCTACTCATTCGGTCT |
| R9#91 | CGCCATGTCTCACACGCGGCCGGCGCAGGCCTGTGAGAT |
| R9#92 | GATTGCCCCTCGACGCAGAGGTCTGAGTTGGTACGAATTC |
| R9#93 | CAGACGCACCTGGCGCAAGGTTCTGAGGATCAGTTACGTG |
| R9#94 | ATTGCCCCCTGACGCAAGGGTAGAGCTACTCATTCGGTCG |
| R9#95 | GCTGCCGATAATTGACGCCCGTGTTGCTCGGTCAATCATG |
| R9#96 | ATCTCCGATTGCCCCACGACGCAGTGGTCGGAGTTACTTC |
| R9#97 | CAGACGCACCTGGCGCAAGGTTCTGAAGATCAGTTACGGG |
| R9#98 | ACGCGCCGATCGCAGGCTAGAGCTAGTGGCTCAGGTTTAT |
| R9#99 | ACGCGCCGATCGCAGGCTAGAGCTAATGGCTCAGGCTTAT |
| R9#100 | ACCTCCGATTGCCCCACGACGCAGTGGTCGGAGTTACTTT |
| R10#1 | TCTTCCCATTCGGAGGCCTGCGCCGGCCGCGTCCGAGGGT |
| R10#2 | GATTGCCCCTCGACGCAGAGGTCTGAGTTGGTACAAATTA |
| R10#3 | GATCCCCGTGTAACCGCTTTTCGCCGCAGAAATTACACTT |
| R10#4 | CACGCGACCGGCGCAGGTCTGAGGGCAGGTCCAGTAATAT |
| R10#5 | GATTGCCCCTCGACGCAGAGGTCTGAGTTGGTACAAATTC |
| R10#6 | ACGCGCCGATCGCAGGCTAGAGCTAATGGCTCAGGTTTAT |
| R10#7 | GATTGCCCCTCGACGCAGAGGTCTGAGTTGGTACAAACTC |
| R10#8 | CAGACGCACCTGGCGCAAGGTTCTGAAGATCAGTTACGTG |
| R10#9 | CCGACGCACCCGGCGCAGGGTTCGGAGATCTAGTTAATTA |
| R10#10 | ACTTGGACCCGATGCCCGTGGCGCAACGGTCGGGTCCTTC |
| R10#11 | GATTGCCCCTCGACGCAGAGGTCTGAGTTGGTACGAACTC |
| R10#12 | TGATCGTCCGGACGCGTGTGGCGCAACACTCCGAGACGAA |
| R10#13 | GATTGCCCCTCGACGCAGAGGTCTGAGCTGGTACAAACTC |
| R10#14 | GATTGCCCCTCGACGCAGAGGTCTGAGTTGGTACAAGCTC |
| R10#15 | GATTGCCCCTCGACGCAGAGGTCTGAGTTGGTACAAACTA |
| R10#16 | GGCCTAGAGATTGCCGGTCGACGCAGACCTCTCTTGGATG |
| R10#17 | ATCTCCGATTGCCCCACGACGCAGTGGTCGGAGTTACTTT |
| R10#18 | CGGGTTCACCGTTGCGTCACGGGGCAATTCACCACTCGCC |
| R10#19 | GACTCCGGGACATGCCCATGTCGCAATGGTGGCCCGGATT |
| R10#20 | GATTGCCCCTCGACGCAGAGGTCAGAGTTGGTACAAATTA |
| R10#21 | GTTCGATTGCCGGGCGACGCAGCCCTCGAACTCTCAGACG |
| R10#22 | ATCGCCCCCTGACGCAAGGGTAGAGCTACTCATTCGGTCG |
| R10#23 | GATTGCCCCTCGACGCAGAGGTCTGAGTTGGTACAAACTT |
| R10#24 | GTGACGCGGTCGACGCAGACCTCACTGTGGGCAGTAACGG |
| R10#25 | ACCGCTGTATGACGCAATACTAGAGCCACGATGCCTTCAG |
| R10#26 | CATGAACGCTCCGGTTGCAGGATTCATGAAGCGCGTTAA |
| R10#27 | TCGATGAGCCGAGGCAGGGAACTAGGTACGCAAGCTCAC |
| R10#28 | AATAGCCCCCTGACGCAAGGGTTAGAGCAGTAATCATGGT |
| R10#29 | CACAACGGGATTGCCCTCTGACGCAAGAGTCCCGTTCTG |
| R10#30 | TCGCCAATGTGACGCTTCCGGCGCAGGAATCACATTCTTC |
| R10#31 | TTGGAACACCAATCCTGCAACCGGAGCGTTGGTGTTACAA |
| R10#32 | GATTGCCCCTCGACGCAGAGGTCTGAGTAGGTACAAACTC |
| R10#33 | ACGCGCCGATCGCAGGCTAGAGCTAATGGCTCAGGTTCA |
| R10#34 | ACGCGCCGATCGCAGGCTAGAGCTAATGGTTCAGGTTTAT |
| R10#35 | CCGACGCACCCGGCGCAGGGTTCGGAGATCTGGTTAATTA |
| R10#36 | GATTGCCCCTCGACGCAGAGGTCTGAGTTGGTACAAACCC |
| R10#37 | CAGACGCACCTGGCGCAAGGTTCTGAAGATCAGTTACGTA |
| R10#38 | TCTTCCCACTCGGAGGCCTGCGCCGGCCGCGTCCGAGGGT |
| R10#39 | CACTATTCGAAACGCATCCGGCGCAGGATTTTCGAATCTC |
| R10#40 | ACTTCCCATTCGGAGGCCTGCGCCGGCCGCGTCCGAGGGT |
| R10#41 | CACGCGACCGGCGCAGGTCTGAGGGCAGGTCCAGTAATAC |
| R10#42 | GCCAGAACGCACCGATCGCAGGTTTCTGTGAGCAGGGGG |
| R10#43 | ACGCGCCGATCGCAGGCTAAGCGGGGGCGGGGGAAGAG |
| R10#44 | GATTGCCCCTCGACGCAGAGGTCAGAGTTGGTACAAACTC |
| R10#45 | GATTGCCCCTCGACGCAGAGGTCTGAGTTGGTACAGACTC |
| R10#46 | GATTGCCCCTCGACGCAGAGGTCTGAGTTGGTACAAATAT |
| R10#47 | ACGCGCCGATCGCAGGCTAGAGCTAATGGCTTAGGTTTAT |
| R10#48 | GAACGCACGGATCGCCGTTTCAGGGCCGGTGCAGTGCTAC |
| R10#49 | CACCGCTATCCGCCGCAGGATTGGGAGCATAGATACACGC |
| R10#50 | CGATGGCGCGTACGCATGTGGCGCAACATTACGCGCCCAT |
| R10#51 | GATTGCCCCTCGACGCAGAGGTCTGAGTTGGTACGAATTC |
| R10#52 | ACGCGCCGATCGCAGGCTATGGGCGGGCTCGGAGTCATTT |
| R10#53 | GATTGCCCCTCGACGCAGAGGTCTGAGCTGGTACAAATTC |
| R10#54 | ATCGCCCCCTGACGCAAGGGTAGAGCTACTCATTCGGTCA |
| R10#55 | CACGCGACCGGCGCAGGTCTGAGGGCAGATCCAGTAATAT |
| R10#56 | GATTGCCCCTCGACGCAGAGGTCTGAGTTGGTACAGATTC |
| R10#57 | GATTGCCCCTCGACGCAGAGGTCTGAGTTGGTACAGATTA |
| R10#58 | ACGCGCCGATCGCAGGCTAGAGCTAATGGCTCAGGTTTAC |
| R10#59 | ACAGATGTTGCCCTCCGACGCAGGAGCATCTAAACTGCAC |
| R10#60 | TCTTCCCATTCGGAGGCCTGCGCCGGCCGCGTCCGCGGGT |
| R10#61 | GATTGCCCCTCGACGCAGAGGTCTGAGTTGGTACAAATTG |
| R10#62 | ACTTTAGGGCTAACCTGCGATCGGTGCGTACCCCATGCTC |
| R10#63 | ATCATTCACGCGGCCGACGCAGGCCTGAAAGATATCCTCG |
| R10#64 | GATTGCCCCTCGACGCAGAGGTCTGAGCTGGTACAAATTA |
| R10#65 | GATTGCCCCTCGACGCAGAGGTCTGAGTTGGTACAAACTG |
| R10#66 | GTTATTGGAGTTGCCCCGTGACGCAACGGCTCCAAAACTGA |
| R10#67 | GATTGCCCCTCGACGCAGAGGTCTGAGTTGGTACAAATTT |
| R10#68 | GATCCCCGTGTAACCGCTTTTCGCCGCAGAAATTACACCT |
| R10#69 | CAGACGCACCTGGCGCAAGGTTCTGAAGATCAGTTACGCG |
| R10#70 | ACGCGCCGATCGCAGGCTAGAGCTAATGGCTCAGGTTTCT |
| R10#71 | GATTGCCCCTCGACGCAGAGGTCTGAGTTGGTACAAGTTA |
| R10#72 | AGTGAGTTGCTCCCCGACGCAGGGGCTCACGCTTGCTTA |
| R10#73 | GATTGCCCCTCGACGCAGAGGTCTGAGTTGGTACACATTA |
| R10#74 | ACGCGCCGATCGCAGGCTAGAGCTAATGGCTCAGGTCTAT |
| R10#75 | TACAGCACCAAGCAACGCACCGATCGCAGGTTTGCTTGTG |
| R10#76 | TCTTCCCATTCGGAGGCCTGCGCCGGCCGCGTCCGAAGGT |
| R10#77 | TAGGGCGATTAACGCAACCGGCGCAGGTTTTAAACGCGCT |
| R10#78 | CACGCGACCGGCGCAGGTCTGAGGGCAGGTCCAGTAGTAT |
| R10#79 | GATTGCCCCTCGACGCAGAGGTCTGAGTTGGTACGAATTA |
| R10#80 | CAGACGCACCTGGCGCAAGGTTCTGAAGATCAGTTACGTT |
| R10#81 | CACGCGACCGGCGCAGGTCTGAGGGCAGGTCCAGTAATAA |
| R10#82 | GATCCCCGTGTAACCGCTTTTCGCCGCAGACATTACACTT |
| R10#83 | ATCTCCGATTGCCCCACGACGCAGTGGTCGGAGTTACTAT |
| R10#84 | GATTGCCCCTCGACGCAGAGGTCTGAGTTGGTACTAATTC |
| R10#85 | CCGACGCACCCGGCGCAGGGTTCGGAGATCCAGTAATAT |
| R10#86 | ATCTCCGATTGCCCCACGACGCAGTGGTCGGAGATACTTT |
| R10#87 | GTGTAGGATCATCGCCCCCACGACGCAGTGGGTGATCGTA |
| R10#88 | CACGCGACCGGCGCAGGTCTGAGGGCAGGCCCAGTAATAT |
| R10#89 | CAGACGCACCTGGCGCAAGGTTCTGAAGATCAGTTACGGG |
| R10#90 | TCTTCCCATTCGGAGGCCTGCGCCGGCCGCGTCCGAGGGG |
| R10#91 | GATTGCCCCTCGACGCAGAGGTCTGAGTTGGTACACATTC |
| R10#92 | TTGCTGCCATGGGTCCTGCGTCGGACGCGCCATGTGGAAT |
| R10#93 | ACGCGCCGATCGCAGGCTAGAGCTAATGGCCCAGGTTTAT |
| R10#94 | GGTGGCTCGAACCTTGCGTCAGGTGCATCGGCCACATTGG |
| R10#95 | CACGCGACCGGCGCAGGTCTGAGGGCAGGTCCAGTAATAG |
| R10#96 | ACGCGCCGATCGCAGGCTAGAGCTAATGGCTCAGGTTTAA |
| R10#97 | CAGACGCACCTGGCGCAAGGTTCTGAAGATCAGTTACGTC |
| R10#98 | AGTGAGTTGCCCCCCGACGCAGGGGCTCACGCTTGCTTA |
| R10#99 | ATCGTTGACCAGGACGCGTTCGGCGCAGAACTCATGGTCA |
| R10#100 | ACGCGCCGATCGCAGGCTAGAGCTAATGACTCAGGTTTAT |

^a^ Candidate aptamer sequences were taken from Reference(Schütze T, Wilhelm B, Greiner N, Braun H, Peter F, et al. (2011) Probing the SELEX Process with Next-Generation Sequencing. PLoS ONE 6: e29604. doi: 10.1371/journal.pone.0029604.). Sequences R1#11,R1#60,R2#55,R2#63 and R2#94 without pair structure were deleted from the data set.
