# Supplementary material for: Support Vector Machine Classification of Streptavidin-Binding Aptamers
Source: PLoS One. 2014 Jun 13;9(6):e99964. doi: 10.1371/journal.pone.0099964 (PMC4057401; doi:10.1371/journal.pone.0099964)
Supplement: Table S2 — List of the topological charge index JGI2 , topological descriptor PW4 , connectivity index X3A , the free energy E , and predicted class labels for 995 candidate aptamer sequences. (DOC) [file pone.0099964.s002.doc]

**Table S2**  **Molecular descriptors used and class labels predicted.**

| **No.** | ***E*(kcal/mol)** | ***PW4*** | ***X3A*** | ***JGI2*** | **Prediction** |
| --- | --- | --- | --- | --- | --- |
| R1#1 | -10.8 | 0.176 | 0.173 | 0.083 | 2 |
| R1#2 | -6.5 | 0.176 | 0.174 | 0.087 | 1 |
| R1#3 | -5.0 | 0.183 | 0.169 | 0.082 | 1 |
| R1#4 | -1.4 | 0.183 | 0.170 | 0.085 | 1 |
| R1#5 | -1.1 | 0.179 | 0.172 | 0.085 | 1 |
| R1#6 | -2.1 | 0.178 | 0.172 | 0.087 | 1 |
| R1#7 | -3.7 | 0.173 | 0.175 | 0.088 | 1 |
| R1#8 | -6.0 | 0.176 | 0.173 | 0.086 | 1 |
| R1#9 | -4.0 | 0.175 | 0.174 | 0.087 | 1 |
| R1#10 | -2.9 | 0.180 | 0.171 | 0.084 | 1 |
| R1#12 | -6.0 | 0.182 | 0.170 | 0.083 | 1 |
| R1#13 | -6.6 | 0.181 | 0.170 | 0.083 | 1 |
| R1#14 | -11.0 | 0.178 | 0.172 | 0.086 | 1 |
| R1#15 | -11.7 | 0.185 | 0.168 | 0.081 | 1 |
| R1#16 | -4.5 | 0.176 | 0.173 | 0.087 | 1 |
| R1#17 | -6.7 | 0.182 | 0.170 | 0.084 | 1 |
| R1#18 | -0.7 | 0.176 | 0.173 | 0.085 | 1 |
| R1#19 | -2.2 | 0.183 | 0.170 | 0.083 | 1 |
| R1#20 | -2.2 | 0.174 | 0.175 | 0.085 | 1 |
| R1#21 | -0.5 | 0.176 | 0.174 | 0.087 | 1 |
| R1#22 | -0.8 | 0.176 | 0.173 | 0.086 | 1 |
| R1#23 | -4.3 | 0.181 | 0.171 | 0.086 | 1 |
| R1#24 | -4.1 | 0.182 | 0.170 | 0.085 | 1 |
| R1#25 | -5.2 | 0.185 | 0.168 | 0.082 | 1 |
| R1#26 | -4.6 | 0.176 | 0.173 | 0.085 | 1 |
| R1#27 | -4.0 | 0.178 | 0.173 | 0.087 | 1 |
| R1#28 | -4.2 | 0.180 | 0.171 | 0.085 | 1 |
| R1#29 | -2.9 | 0.177 | 0.173 | 0.086 | 1 |
| R1#30 | -1.3 | 0.176 | 0.173 | 0.086 | 1 |
| R1#31 | -6.6 | 0.175 | 0.174 | 0.088 | 1 |
| R1#32 | -6.2 | 0.179 | 0.172 | 0.085 | 1 |
| R1#33 | -10.1 | 0.182 | 0.170 | 0.084 | 1 |
| R1#34 | -4.9 | 0.183 | 0.170 | 0.085 | 1 |
| R1#35 | -6.0 | 0.181 | 0.170 | 0.082 | 1 |
| R1#36 | -6.4 | 0.180 | 0.171 | 0.085 | 1 |
| R1#37 | -3.6 | 0.172 | 0.176 | 0.091 | 1 |
| R1#38 | -7.7 | 0.178 | 0.172 | 0.085 | 1 |
| R1#39 | -2.5 | 0.180 | 0.171 | 0.086 | 1 |
| R1#40 | -6.3 | 0.183 | 0.170 | 0.085 | 1 |
| R1#41 | -4.7 | 0.181 | 0.170 | 0.081 | 1 |
| R1#42 | -1.6 | 0.180 | 0.171 | 0.085 | 1 |
| R1#43 | -3.7 | 0.183 | 0.170 | 0.085 | 1 |
| R1#44 | -4.0 | 0.177 | 0.172 | 0.083 | 1 |
| R1#45 | -2.0 | 0.179 | 0.172 | 0.084 | 1 |
| R1#46 | -2.6 | 0.175 | 0.174 | 0.088 | 1 |
| R1#47 | -5.2 | 0.182 | 0.170 | 0.084 | 1 |
| R1#48 | -13.9 | 0.183 | 0.170 | 0.085 | 1 |
| R1#49 | -5.6 | 0.182 | 0.170 | 0.085 | 1 |
| R1#50 | -0.7 | 0.176 | 0.173 | 0.088 | 1 |
| R1#51 | -8.6 | 0.180 | 0.171 | 0.086 | 1 |
| R1#52 | -8.0 | 0.175 | 0.174 | 0.087 | 1 |
| R1#53 | -1.7 | 0.184 | 0.169 | 0.083 | 1 |
| R1#54 | -5.6 | 0.180 | 0.171 | 0.085 | 1 |
| R1#55 | -3.8 | 0.184 | 0.169 | 0.083 | 1 |
| R1#56 | -0.8 | 0.181 | 0.171 | 0.085 | 1 |
| R1#57 | -6.3 | 0.179 | 0.172 | 0.085 | 1 |
| R1#58 | -6.0 | 0.176 | 0.173 | 0.086 | 1 |
| R1#59 | -1.9 | 0.186 | 0.169 | 0.084 | 1 |
| R1#61 | -4.7 | 0.183 | 0.169 | 0.083 | 1 |
| R1#62 | -9.0 | 0.182 | 0.170 | 0.083 | 1 |
| R1#63 | -1.5 | 0.175 | 0.174 | 0.087 | 1 |
| R1#64 | -5.8 | 0.176 | 0.173 | 0.085 | 1 |
| R1#65 | -4.6 | 0.182 | 0.170 | 0.084 | 1 |
| R1#66 | -2.9 | 0.179 | 0.172 | 0.085 | 1 |
| R1#67 | -4.5 | 0.184 | 0.169 | 0.084 | 1 |
| R1#68 | -11.8 | 0.178 | 0.172 | 0.085 | 1 |
| R1#69 | -8.0 | 0.176 | 0.174 | 0.086 | 1 |
| R1#70 | -3.2 | 0.180 | 0.171 | 0.086 | 1 |
| R1#71 | -6.7 | 0.184 | 0.169 | 0.081 | 1 |
| R1#72 | -5.2 | 0.178 | 0.172 | 0.086 | 1 |
| R1#73 | -9.3 | 0.184 | 0.169 | 0.083 | 1 |
| R1#74 | -6.5 | 0.173 | 0.175 | 0.088 | 1 |
| R1#75 | -7.7 | 0.176 | 0.174 | 0.087 | 1 |
| R1#76 | -6.2 | 0.183 | 0.169 | 0.082 | 1 |
| R1#77 | -9.1 | 0.176 | 0.173 | 0.086 | 1 |
| R1#78 | -2.1 | 0.179 | 0.172 | 0.085 | 1 |
| R1#79 | -2.1 | 0.176 | 0.173 | 0.086 | 1 |
| R1#80 | -2.6 | 0.184 | 0.169 | 0.082 | 1 |
| R1#81 | -3.3 | 0.173 | 0.175 | 0.089 | 1 |
| R1#82 | -5.7 | 0.183 | 0.170 | 0.083 | 1 |
| R1#83 | -4.5 | 0.173 | 0.175 | 0.086 | 1 |
| R1#84 | -8.3 | 0.178 | 0.172 | 0.085 | 1 |
| R1#85 | -2.0 | 0.178 | 0.172 | 0.086 | 1 |
| R1#86 | -4.8 | 0.181 | 0.171 | 0.084 | 1 |
| R1#87 | -6.5 | 0.183 | 0.170 | 0.086 | 1 |
| R1#88 | -0.9 | 0.175 | 0.174 | 0.088 | 1 |
| R1#89 | -4.5 | 0.183 | 0.170 | 0.084 | 1 |
| R1#90 | -8.2 | 0.182 | 0.170 | 0.083 | 1 |
| R1#91 | -3.6 | 0.180 | 0.171 | 0.085 | 1 |
| R1#92 | -6.5 | 0.186 | 0.168 | 0.083 | 1 |
| R1#93 | -3.0 | 0.176 | 0.173 | 0.085 | 1 |
| R1#94 | -2.3 | 0.178 | 0.172 | 0.087 | 1 |
| R1#95 | -2.7 | 0.176 | 0.173 | 0.086 | 1 |
| R1#96 | -4.0 | 0.177 | 0.172 | 0.084 | 1 |
| R1#97 | -5.0 | 0.181 | 0.171 | 0.084 | 1 |
| R1#98 | -1.2 | 0.180 | 0.171 | 0.086 | 1 |
| R1#99 | -8.3 | 0.179 | 0.171 | 0.084 | 1 |
| R1#100 | -1.9 | 0.181 | 0.171 | 0.085 | 1 |
| R2#1 | -10.1 | 0.177 | 0.173 | 0.084 | 1 |
| R2#2 | -3.2 | 0.182 | 0.170 | 0.083 | 1 |
| R2#3 | -6.5 | 0.176 | 0.174 | 0.087 | 1 |
| R2#4 | -9.3 | 0.176 | 0.173 | 0.083 | 2 |
| R2#5 | -3.5 | 0.180 | 0.171 | 0.085 | 1 |
| R2#6 | -4.2 | 0.173 | 0.175 | 0.086 | 1 |
| R2#7 | -1.9 | 0.182 | 0.170 | 0.084 | 1 |
| R2#8 | -4.2 | 0.180 | 0.171 | 0.084 | 1 |
| R2#9 | -7.0 | 0.179 | 0.172 | 0.084 | 2 |
| R2#10 | -9.4 | 0.179 | 0.172 | 0.085 | 1 |
| R2#11 | -6.7 | 0.185 | 0.168 | 0.081 | 1 |
| R2#12 | -5.0 | 0.181 | 0.171 | 0.086 | 1 |
| R2#13 | -1.7 | 0.179 | 0.171 | 0.084 | 1 |
| R2#14 | -5.6 | 0.177 | 0.172 | 0.083 | 2 |
| R2#15 | -2.7 | 0.178 | 0.172 | 0.085 | 1 |
| R2#16 | -1.9 | 0.182 | 0.170 | 0.084 | 1 |
| R2#17 | -2.2 | 0.182 | 0.170 | 0.084 | 1 |
| R2#18 | -7.1 | 0.185 | 0.168 | 0.082 | 2 |
| R2#19 | -6.4 | 0.178 | 0.173 | 0.087 | 2 |
| R2#20 | -5.7 | 0.177 | 0.173 | 0.084 | 2 |
| R2#21 | -4.0 | 0.180 | 0.171 | 0.084 | 2 |
| R2#22 | -2.8 | 0.176 | 0.174 | 0.085 | 1 |
| R2#23 | -9.5 | 0.180 | 0.171 | 0.084 | 2 |
| R2#24 | -5.5 | 0.182 | 0.170 | 0.084 | 2 |
| R2#25 | -1.3 | 0.181 | 0.171 | 0.087 | 1 |
| R2#26 | -2.4 | 0.185 | 0.169 | 0.083 | 1 |
| R2#27 | -2.5 | 0.185 | 0.169 | 0.083 | 2 |
| R2#28 | -2.5 | 0.180 | 0.171 | 0.085 | 1 |
| R2#29 | -5.5 | 0.182 | 0.170 | 0.084 | 2 |
| R2#30 | -7.9 | 0.181 | 0.171 | 0.086 | 2 |
| R2#31 | -1.7 | 0.176 | 0.173 | 0.086 | 1 |
| R2#32 | -5.3 | 0.178 | 0.172 | 0.085 | 1 |
| R2#33 | -3.9 | 0.181 | 0.171 | 0.085 | 1 |
| R2#34 | -8.4 | 0.179 | 0.172 | 0.085 | 2 |
| R2#35 | -6.2 | 0.181 | 0.170 | 0.082 | 2 |
| R2#36 | -3.7 | 0.181 | 0.170 | 0.084 | 1 |
| R2#37 | -9.6 | 0.180 | 0.171 | 0.085 | 2 |
| R2#38 | -7.6 | 0.183 | 0.170 | 0.082 | 2 |
| R2#39 | -3.1 | 0.179 | 0.171 | 0.084 | 1 |
| R2#40 | -4.3 | 0.173 | 0.175 | 0.088 | 2 |
| R2#41 | -10.1 | 0.177 | 0.173 | 0.084 | 1 |
| R2#42 | -8.8 | 0.178 | 0.172 | 0.085 | 1 |
| R2#43 | -5.8 | 0.176 | 0.173 | 0.087 | 1 |
| R2#44 | -11.3 | 0.177 | 0.173 | 0.084 | 1 |
| R2#45 | -7.7 | 0.178 | 0.172 | 0.085 | 1 |
| R2#46 | -3.1 | 0.181 | 0.171 | 0.084 | 1 |
| R2#47 | -9.8 | 0.181 | 0.171 | 0.084 | 1 |
| R2#48 | -2.8 | 0.173 | 0.175 | 0.089 | 1 |
| R2#49 | -0.9 | 0.184 | 0.169 | 0.084 | 1 |
| R2#50 | -7.9 | 0.182 | 0.170 | 0.083 | 2 |
| R2#51 | -6.2 | 0.178 | 0.172 | 0.084 | 2 |
| R2#52 | -5.1 | 0.177 | 0.173 | 0.083 | 2 |
| R2#53 | -2.0 | 0.178 | 0.172 | 0.087 | 1 |
| R2#54 | -5.7 | 0.177 | 0.173 | 0.086 | 2 |
| R2#56 | -1.9 | 0.179 | 0.171 | 0.083 | 2 |
| R2#57 | -12.8 | 0.183 | 0.169 | 0.083 | 1 |
| R2#58 | -4.5 | 0.176 | 0.173 | 0.085 | 1 |
| R2#59 | -4.4 | 0.183 | 0.170 | 0.084 | 1 |
| R2#60 | -5.5 | 0.178 | 0.173 | 0.086 | 2 |
| R2#61 | -1.8 | 0.179 | 0.172 | 0.083 | 1 |
| R2#62 | -2.0 | 0.181 | 0.171 | 0.084 | 1 |
| R2#64 | -9.4 | 0.179 | 0.172 | 0.084 | 1 |
| R2#65 | -1.8 | 0.187 | 0.168 | 0.083 | 1 |
| R2#66 | -4.9 | 0.179 | 0.171 | 0.084 | 2 |
| R2#67 | -5.2 | 0.180 | 0.171 | 0.086 | 1 |
| R2#68 | -6.8 | 0.173 | 0.175 | 0.087 | 1 |
| R2#69 | -3.9 | 0.184 | 0.169 | 0.082 | 1 |
| R2#70 | -5.6 | 0.185 | 0.168 | 0.083 | 2 |
| R2#71 | -7.1 | 0.182 | 0.170 | 0.083 | 2 |
| R2#72 | -8.2 | 0.177 | 0.173 | 0.087 | 2 |
| R2#73 | -4.4 | 0.179 | 0.172 | 0.086 | 1 |
| R2#74 | -1.0 | 0.179 | 0.171 | 0.084 | 1 |
| R2#75 | -2.5 | 0.181 | 0.171 | 0.085 | 1 |
| R2#76 | -2.5 | 0.180 | 0.171 | 0.085 | 1 |
| R2#77 | -2.7 | 0.178 | 0.172 | 0.086 | 1 |
| R2#78 | -3.9 | 0.185 | 0.169 | 0.084 | 1 |
| R2#79 | -7.4 | 0.185 | 0.168 | 0.081 | 2 |
| R2#80 | -5.5 | 0.184 | 0.168 | 0.081 | 2 |
| R2#81 | -6.5 | 0.179 | 0.172 | 0.085 | 1 |
| R2#82 | -2.8 | 0.179 | 0.172 | 0.087 | 1 |
| R2#83 | -2.4 | 0.175 | 0.174 | 0.088 | 1 |
| R2#84 | -2.6 | 0.181 | 0.170 | 0.083 | 1 |
| R2#85 | -0.3 | 0.178 | 0.172 | 0.085 | 1 |
| R2#86 | -2.7 | 0.177 | 0.173 | 0.084 | 1 |
| R2#87 | -1.8 | 0.182 | 0.171 | 0.086 | 1 |
| R2#88 | -1.4 | 0.182 | 0.170 | 0.085 | 1 |
| R2#89 | -9.1 | 0.183 | 0.169 | 0.084 | 2 |
| R2#90 | -6.4 | 0.180 | 0.171 | 0.084 | 1 |
| R2#91 | -3.4 | 0.181 | 0.171 | 0.086 | 1 |
| R2#92 | -2.4 | 0.180 | 0.171 | 0.085 | 1 |
| R2#93 | -0.3 | 0.179 | 0.172 | 0.086 | 1 |
| R2#95 | -5.1 | 0.185 | 0.168 | 0.083 | 2 |
| R2#96 | -6.5 | 0.178 | 0.172 | 0.085 | 1 |
| R2#97 | -9.0 | 0.176 | 0.173 | 0.085 | 2 |
| R2#98 | -6.6 | 0.182 | 0.170 | 0.083 | 1 |
| R2#99 | -5.1 | 0.180 | 0.171 | 0.084 | 2 |
| R2#100 | -0.8 | 0.181 | 0.170 | 0.083 | 1 |
| R3#1 | -2.9 | 0.177 | 0.173 | 0.085 | 1 |
| R3#2 | -7.9 | 0.179 | 0.172 | 0.084 | 2 |
| R3#3 | -3.5 | 0.180 | 0.171 | 0.085 | 1 |
| R3#4 | -10.1 | 0.177 | 0.173 | 0.084 | 1 |
| R3#5 | -5.2 | 0.182 | 0.170 | 0.083 | 2 |
| R3#6 | -5.1 | 0.183 | 0.170 | 0.082 | 2 |
| R3#7 | -7.0 | 0.181 | 0.171 | 0.083 | 2 |
| R3#8 | -5.2 | 0.177 | 0.172 | 0.082 | 2 |
| R3#9 | -4.2 | 0.180 | 0.171 | 0.084 | 1 |
| R3#10 | -6.5 | 0.176 | 0.174 | 0.087 | 1 |
| R3#11 | -1.9 | 0.183 | 0.169 | 0.083 | 2 |
| R3#12 | -4.9 | 0.182 | 0.170 | 0.082 | 2 |
| R3#13 | -7.0 | 0.182 | 0.170 | 0.084 | 2 |
| R3#14 | -5.2 | 0.181 | 0.171 | 0.083 | 2 |
| R3#15 | -7.0 | 0.179 | 0.172 | 0.084 | 2 |
| R3#16 | -5.7 | 0.177 | 0.173 | 0.084 | 2 |
| R3#17 | -4.2 | 0.173 | 0.175 | 0.086 | 1 |
| R3#18 | -6.4 | 0.176 | 0.173 | 0.085 | 2 |
| R3#19 | -11.8 | 0.182 | 0.170 | 0.083 | 2 |
| R3#20 | -5.6 | 0.183 | 0.170 | 0.082 | 2 |
| R3#21 | -2.9 | 0.177 | 0.173 | 0.085 | 1 |
| R3#22 | -7.6 | 0.179 | 0.172 | 0.084 | 2 |
| R3#23 | -3.9 | 0.180 | 0.171 | 0.085 | 1 |
| R3#24 | -4.8 | 0.184 | 0.169 | 0.084 | 1 |
| R3#25 | -3.2 | 0.182 | 0.170 | 0.083 | 1 |
| R3#26 | -6.0 | 0.182 | 0.170 | 0.082 | 1 |
| R3#27 | -3.9 | 0.180 | 0.171 | 0.085 | 1 |
| R3#28 | -6.5 | 0.181 | 0.170 | 0.082 | 1 |
| R3#29 | -7.4 | 0.179 | 0.171 | 0.083 | 2 |
| R3#30 | -2.7 | 0.178 | 0.172 | 0.085 | 1 |
| R3#31 | -6.2 | 0.182 | 0.170 | 0.083 | 1 |
| R3#32 | -2.9 | 0.177 | 0.173 | 0.085 | 1 |
| R3#33 | -9.3 | 0.176 | 0.173 | 0.086 | 2 |
| R3#34 | -2.9 | 0.177 | 0.173 | 0.085 | 1 |
| R3#35 | -4.0 | 0.182 | 0.170 | 0.084 | 2 |
| R3#36 | -8.1 | 0.180 | 0.171 | 0.084 | 2 |
| R3#37 | -1.7 | 0.179 | 0.171 | 0.084 | 1 |
| R3#38 | -5.7 | 0.180 | 0.171 | 0.085 | 2 |
| R3#39 | -6.2 | 0.174 | 0.174 | 0.083 | 2 |
| R3#40 | -5.2 | 0.177 | 0.172 | 0.082 | 2 |
| R3#41 | -2.8 | 0.176 | 0.174 | 0.085 | 1 |
| R3#42 | -4.3 | 0.179 | 0.171 | 0.084 | 2 |
| R3#43 | -2.2 | 0.180 | 0.171 | 0.085 | 1 |
| R3#44 | -9.4 | 0.179 | 0.172 | 0.085 | 1 |
| R3#45 | -10.1 | 0.177 | 0.173 | 0.084 | 1 |
| R3#46 | -4.8 | 0.179 | 0.172 | 0.085 | 1 |
| R3#47 | -2.4 | 0.185 | 0.169 | 0.083 | 1 |
| R3#48 | -2.9 | 0.179 | 0.172 | 0.086 | 1 |
| R3#49 | -15.0 | 0.176 | 0.173 | 0.087 | 1 |
| R3#50 | -2.5 | 0.180 | 0.171 | 0.085 | 1 |
| R3#51 | -6.7 | 0.185 | 0.168 | 0.081 | 1 |
| R3#52 | -8.2 | 0.177 | 0.172 | 0.083 | 2 |
| R3#53 | -4.0 | 0.180 | 0.171 | 0.084 | 2 |
| R3#54 | -5.0 | 0.181 | 0.171 | 0.086 | 1 |
| R3#55 | -9.5 | 0.176 | 0.173 | 0.086 | 2 |
| R3#56 | -8.5 | 0.180 | 0.171 | 0.085 | 2 |
| R3#57 | -5.1 | 0.179 | 0.171 | 0.083 | 2 |
| R3#58 | -2.9 | 0.181 | 0.171 | 0.083 | 2 |
| R3#59 | -2.5 | 0.185 | 0.169 | 0.083 | 2 |
| R3#60 | -5.0 | 0.185 | 0.169 | 0.084 | 1 |
| R3#61 | -2.9 | 0.181 | 0.171 | 0.083 | 2 |
| R3#62 | -3.7 | 0.179 | 0.172 | 0.086 | 1 |
| R3#63 | -5.1 | 0.180 | 0.171 | 0.084 | 2 |
| R3#64 | -4.4 | 0.181 | 0.171 | 0.084 | 1 |
| R3#65 | -2.6 | 0.180 | 0.171 | 0.083 | 1 |
| R3#66 | -6.5 | 0.178 | 0.172 | 0.085 | 1 |
| R3#67 | -5.4 | 0.182 | 0.170 | 0.084 | 1 |
| R3#68 | -7.1 | 0.185 | 0.168 | 0.082 | 2 |
| R3#69 | -6.4 | 0.178 | 0.173 | 0.087 | 2 |
| R3#70 | -3.2 | 0.178 | 0.173 | 0.086 | 1 |
| R3#71 | -5.1 | 0.181 | 0.171 | 0.084 | 2 |
| R3#72 | -2.0 | 0.179 | 0.171 | 0.084 | 1 |
| R3#73 | -3.1 | 0.182 | 0.170 | 0.084 | 1 |
| R3#74 | -7.2 | 0.179 | 0.172 | 0.084 | 2 |
| R3#75 | -4.5 | 0.181 | 0.171 | 0.083 | 1 |
| R3#76 | -5.8 | 0.181 | 0.171 | 0.084 | 2 |
| R3#77 | -2.2 | 0.179 | 0.172 | 0.086 | 1 |
| R3#78 | -5.4 | 0.177 | 0.173 | 0.086 | 1 |
| R3#79 | -4.5 | 0.174 | 0.174 | 0.084 | 1 |
| R3#80 | -7.8 | 0.176 | 0.173 | 0.086 | 2 |
| R3#81 | -4.0 | 0.182 | 0.170 | 0.084 | 2 |
| R3#82 | -9.5 | 0.180 | 0.171 | 0.084 | 2 |
| R3#83 | -7.7 | 0.181 | 0.171 | 0.085 | 1 |
| R3#84 | -7.1 | 0.179 | 0.171 | 0.084 | 2 |
| R3#85 | -3.1 | 0.178 | 0.172 | 0.086 | 1 |
| R3#86 | -3.5 | 0.182 | 0.170 | 0.084 | 1 |
| R3#87 | -6.6 | 0.181 | 0.171 | 0.085 | 1 |
| R3#88 | -9.3 | 0.176 | 0.173 | 0.083 | 2 |
| R3#89 | -8.7 | 0.181 | 0.171 | 0.085 | 2 |
| R3#90 | -5.5 | 0.182 | 0.170 | 0.084 | 2 |
| R3#91 | -2.9 | 0.181 | 0.171 | 0.083 | 2 |
| R3#92 | -5.7 | 0.181 | 0.171 | 0.085 | 2 |
| R3#93 | -3.2 | 0.178 | 0.173 | 0.084 | 1 |
| R3#94 | -4.0 | 0.182 | 0.170 | 0.084 | 2 |
| R3#95 | -4.7 | 0.182 | 0.170 | 0.083 | 1 |
| R3#96 | -3.8 | 0.179 | 0.172 | 0.085 | 1 |
| R3#97 | -3.9 | 0.181 | 0.171 | 0.085 | 1 |
| R3#98 | -2.9 | 0.177 | 0.173 | 0.085 | 1 |
| R3#99 | -4.4 | 0.183 | 0.170 | 0.082 | 1 |
| R3#100 | -7.6 | 0.178 | 0.172 | 0.085 | 2 |
| R4#1 | -2.9 | 0.181 | 0.171 | 0.083 | 2 |
| R4#2 | -7.9 | 0.179 | 0.172 | 0.084 | 2 |
| R4#3 | -5.2 | 0.182 | 0.170 | 0.083 | 2 |
| R4#4 | -4.9 | 0.182 | 0.170 | 0.082 | 2 |
| R4#5 | -5.2 | 0.177 | 0.172 | 0.082 | 2 |
| R4#6 | -7.0 | 0.181 | 0.171 | 0.083 | 2 |
| R4#7 | -11.8 | 0.182 | 0.170 | 0.083 | 2 |
| R4#8 | -7.0 | 0.182 | 0.170 | 0.084 | 2 |
| R4#9 | -5.6 | 0.183 | 0.170 | 0.082 | 2 |
| R4#10 | -5.1 | 0.183 | 0.170 | 0.082 | 2 |
| R4#11 | -6.4 | 0.176 | 0.173 | 0.085 | 2 |
| R4#12 | -5.2 | 0.181 | 0.171 | 0.083 | 2 |
| R4#13 | -7.6 | 0.179 | 0.172 | 0.084 | 2 |
| R4#14 | -6.5 | 0.181 | 0.170 | 0.082 | 1 |
| R4#15 | -6.2 | 0.181 | 0.170 | 0.082 | 2 |
| R4#16 | -4.8 | 0.184 | 0.169 | 0.084 | 1 |
| R4#17 | -3.9 | 0.180 | 0.171 | 0.085 | 1 |
| R4#18 | -6.0 | 0.182 | 0.170 | 0.082 | 1 |
| R4#19 | -5.2 | 0.177 | 0.172 | 0.083 | 2 |
| R4#20 | -4.3 | 0.179 | 0.171 | 0.084 | 2 |
| R4#21 | -4.0 | 0.182 | 0.170 | 0.084 | 2 |
| R4#22 | -2.9 | 0.181 | 0.171 | 0.083 | 2 |
| R4#23 | -8.1 | 0.181 | 0.170 | 0.082 | 2 |
| R4#24 | -7.4 | 0.179 | 0.171 | 0.083 | 2 |
| R4#25 | -4.7 | 0.179 | 0.172 | 0.083 | 1 |
| R4#26 | -8.1 | 0.181 | 0.170 | 0.082 | 2 |
| R4#27 | -7.4 | 0.181 | 0.171 | 0.085 | 2 |
| R4#28 | -6.2 | 0.182 | 0.170 | 0.083 | 1 |
| R4#29 | -4.9 | 0.181 | 0.171 | 0.083 | 2 |
| R4#30 | -9.3 | 0.176 | 0.173 | 0.086 | 2 |
| R4#31 | -5.7 | 0.180 | 0.171 | 0.085 | 2 |
| R4#32 | -4.8 | 0.182 | 0.170 | 0.085 | 1 |
| R4#33 | -5.1 | 0.181 | 0.170 | 0.083 | 2 |
| R4#34 | -5.0 | 0.185 | 0.169 | 0.084 | 1 |
| R4#35 | -2.9 | 0.181 | 0.171 | 0.083 | 2 |
| R4#36 | -3.9 | 0.180 | 0.171 | 0.085 | 1 |
| R4#37 | -15.0 | 0.176 | 0.173 | 0.085 | 1 |
| R4#38 | -2.9 | 0.181 | 0.171 | 0.083 | 2 |
| R4#39 | -5.7 | 0.181 | 0.171 | 0.085 | 2 |
| R4#40 | -4.8 | 0.178 | 0.172 | 0.084 | 1 |
| R4#41 | -2.9 | 0.181 | 0.171 | 0.083 | 2 |
| R4#42 | -5.8 | 0.183 | 0.170 | 0.082 | 2 |
| R4#43 | -5.4 | 0.177 | 0.173 | 0.086 | 1 |
| R4#44 | -4.5 | 0.181 | 0.171 | 0.083 | 1 |
| R4#45 | -5.1 | 0.181 | 0.170 | 0.082 | 2 |
| R4#46 | -5.6 | 0.181 | 0.170 | 0.082 | 2 |
| R4#47 | -5.4 | 0.182 | 0.170 | 0.084 | 1 |
| R4#48 | -7.7 | 0.181 | 0.171 | 0.085 | 1 |
| R4#49 | -2.2 | 0.180 | 0.171 | 0.085 | 1 |
| R4#50 | -8.7 | 0.179 | 0.172 | 0.084 | 2 |
| R4#51 | -4.9 | 0.182 | 0.170 | 0.082 | 2 |
| R4#52 | -3.3 | 0.177 | 0.173 | 0.086 | 1 |
| R4#53 | -9.3 | 0.176 | 0.173 | 0.083 | 2 |
| R4#54 | -9.5 | 0.178 | 0.172 | 0.085 | 2 |
| R4#55 | -7.9 | 0.179 | 0.172 | 0.084 | 2 |
| R4#56 | -9.6 | 0.182 | 0.170 | 0.083 | 2 |
| R4#57 | -4.4 | 0.181 | 0.171 | 0.084 | 1 |
| R4#58 | -7.4 | 0.181 | 0.171 | 0.085 | 2 |
| R4#59 | -5.8 | 0.182 | 0.170 | 0.083 | 2 |
| R4#60 | -4.0 | 0.182 | 0.170 | 0.084 | 2 |
| R4#61 | -2.6 | 0.180 | 0.171 | 0.083 | 1 |
| R4#62 | -6.5 | 0.179 | 0.172 | 0.086 | 1 |
| R4#63 | -2.9 | 0.181 | 0.171 | 0.083 | 2 |
| R4#64 | -5.1 | 0.181 | 0.171 | 0.084 | 2 |
| R4#65 | -3.6 | 0.180 | 0.171 | 0.084 | 1 |
| R4#66 | -7.9 | 0.179 | 0.172 | 0.084 | 2 |
| R4#67 | -8.5 | 0.181 | 0.170 | 0.082 | 2 |
| R4#68 | -4.9 | 0.182 | 0.170 | 0.082 | 2 |
| R4#69 | -8.6 | 0.179 | 0.171 | 0.084 | 1 |
| R4#70 | -4.1 | 0.182 | 0.170 | 0.084 | 1 |
| R4#71 | -6.6 | 0.181 | 0.171 | 0.085 | 1 |
| R4#72 | -7.3 | 0.174 | 0.174 | 0.084 | 2 |
| R4#73 | -2.9 | 0.181 | 0.171 | 0.083 | 2 |
| R4#74 | -6.4 | 0.182 | 0.170 | 0.084 | 1 |
| R4#75 | -7.4 | 0.181 | 0.171 | 0.085 | 2 |
| R4#76 | -3.5 | 0.182 | 0.170 | 0.084 | 1 |
| R4#77 | -3.1 | 0.182 | 0.170 | 0.084 | 1 |
| R4#78 | -6.3 | 0.182 | 0.170 | 0.084 | 1 |
| R4#79 | -7.9 | 0.179 | 0.172 | 0.084 | 2 |
| R4#80 | -4.4 | 0.179 | 0.172 | 0.084 | 1 |
| R4#81 | -2.9 | 0.181 | 0.171 | 0.083 | 2 |
| R4#82 | -6.4 | 0.183 | 0.169 | 0.082 | 1 |
| R4#83 | -5.1 | 0.183 | 0.169 | 0.082 | 2 |
| R4#84 | -7.5 | 0.178 | 0.172 | 0.085 | 1 |
| R4#85 | -4.3 | 0.179 | 0.171 | 0.084 | 2 |
| R4#86 | -8.8 | 0.177 | 0.173 | 0.084 | 1 |
| R4#87 | -5.7 | 0.182 | 0.170 | 0.084 | 1 |
| R4#88 | -7.0 | 0.182 | 0.170 | 0.084 | 2 |
| R4#89 | -7.9 | 0.179 | 0.172 | 0.084 | 2 |
| R4#90 | -5.5 | 0.182 | 0.170 | 0.084 | 2 |
| R4#91 | -5.2 | 0.182 | 0.170 | 0.083 | 2 |
| R4#92 | -6.0 | 0.182 | 0.170 | 0.082 | 1 |
| R4#93 | -9.9 | 0.182 | 0.170 | 0.083 | 1 |
| R4#94 | -7.9 | 0.179 | 0.172 | 0.084 | 2 |
| R4#95 | -4.4 | 0.178 | 0.172 | 0.086 | 1 |
| R4#96 | -3.1 | 0.178 | 0.172 | 0.086 | 1 |
| R4#97 | -2.7 | 0.182 | 0.170 | 0.084 | 1 |
| R4#98 | -2.9 | 0.181 | 0.171 | 0.083 | 2 |
| R4#99 | -9.7 | 0.177 | 0.173 | 0.086 | 2 |
| R4#100 | -4.0 | 0.182 | 0.170 | 0.084 | 2 |
| R5#1 | -7.9 | 0.179 | 0.172 | 0.084 | 2 |
| R5#2 | -2.9 | 0.181 | 0.171 | 0.083 | 2 |
| R5#3 | -5.2 | 0.182 | 0.170 | 0.083 | 2 |
| R5#4 | -4.9 | 0.182 | 0.170 | 0.082 | 2 |
| R5#5 | -11.8 | 0.182 | 0.170 | 0.083 | 2 |
| R5#6 | -7.0 | 0.182 | 0.170 | 0.084 | 2 |
| R5#7 | -6.4 | 0.176 | 0.173 | 0.085 | 2 |
| R5#8 | -7.0 | 0.181 | 0.171 | 0.083 | 2 |
| R5#9 | -2.9 | 0.181 | 0.171 | 0.083 | 2 |
| R5#10 | -7.4 | 0.179 | 0.171 | 0.083 | 2 |
| R5#11 | -6.0 | 0.182 | 0.170 | 0.082 | 1 |
| R5#12 | -9.3 | 0.176 | 0.173 | 0.083 | 2 |
| R5#13 | -4.0 | 0.182 | 0.170 | 0.084 | 2 |
| R5#14 | -4.8 | 0.184 | 0.169 | 0.084 | 1 |
| R5#15 | -5.2 | 0.177 | 0.172 | 0.082 | 2 |
| R5#16 | -5.2 | 0.177 | 0.172 | 0.082 | 2 |
| R5#17 | -6.5 | 0.181 | 0.170 | 0.082 | 1 |
| R5#18 | -7.4 | 0.181 | 0.171 | 0.085 | 2 |
| R5#19 | -8.1 | 0.180 | 0.171 | 0.084 | 2 |
| R5#20 | -8.7 | 0.183 | 0.170 | 0.082 | 2 |
| R5#21 | -2.9 | 0.181 | 0.171 | 0.083 | 2 |
| R5#22 | -5.1 | 0.181 | 0.170 | 0.083 | 2 |
| R5#23 | -4.9 | 0.182 | 0.170 | 0.082 | 2 |
| R5#24 | -2.9 | 0.181 | 0.171 | 0.083 | 2 |
| R5#25 | -5.2 | 0.181 | 0.171 | 0.083 | 2 |
| R5#26 | -4.3 | 0.179 | 0.171 | 0.084 | 2 |
| R5#27 | -7.9 | 0.181 | 0.171 | 0.085 | 2 |
| R5#28 | -5.8 | 0.182 | 0.170 | 0.083 | 2 |
| R5#29 | -7.7 | 0.181 | 0.171 | 0.085 | 1 |
| R5#30 | -7.6 | 0.179 | 0.172 | 0.084 | 2 |
| R5#31 | -8.7 | 0.181 | 0.171 | 0.085 | 2 |
| R5#32 | -7.9 | 0.181 | 0.171 | 0.085 | 2 |
| R5#33 | -5.6 | 0.183 | 0.170 | 0.082 | 2 |
| R5#34 | -5.2 | 0.182 | 0.170 | 0.083 | 2 |
| R5#35 | -2.9 | 0.181 | 0.171 | 0.083 | 2 |
| R5#36 | -5.8 | 0.183 | 0.170 | 0.082 | 2 |
| R5#37 | -4.0 | 0.182 | 0.170 | 0.084 | 2 |
| R5#38 | -9.6 | 0.182 | 0.170 | 0.083 | 2 |
| R5#39 | -7.6 | 0.183 | 0.170 | 0.082 | 2 |
| R5#40 | -4.7 | 0.180 | 0.171 | 0.085 | 1 |
| R5#41 | -6.2 | 0.182 | 0.170 | 0.083 | 1 |
| R5#42 | -4.9 | 0.182 | 0.170 | 0.082 | 2 |
| R5#43 | -7.9 | 0.181 | 0.171 | 0.085 | 2 |
| R5#44 | -4.9 | 0.181 | 0.171 | 0.083 | 2 |
| R5#45 | -8.1 | 0.177 | 0.172 | 0.083 | 2 |
| R5#46 | -7.9 | 0.181 | 0.171 | 0.085 | 2 |
| R5#47 | -5.6 | 0.177 | 0.172 | 0.083 | 2 |
| R5#48 | -3.9 | 0.180 | 0.171 | 0.085 | 1 |
| R5#49 | -5.1 | 0.183 | 0.170 | 0.082 | 2 |
| R5#50 | -2.9 | 0.181 | 0.171 | 0.083 | 2 |
| R5#51 | -5.4 | 0.182 | 0.170 | 0.084 | 1 |
| R5#52 | -2.9 | 0.181 | 0.171 | 0.083 | 2 |
| R5#53 | -3.2 | 0.178 | 0.173 | 0.084 | 1 |
| R5#54 | -4.9 | 0.182 | 0.170 | 0.082 | 2 |
| R5#55 | -4.9 | 0.182 | 0.170 | 0.082 | 2 |
| R5#56 | -9.7 | 0.177 | 0.173 | 0.086 | 2 |
| R5#57 | -4.5 | 0.181 | 0.171 | 0.083 | 1 |
| R5#58 | -7.4 | 0.181 | 0.171 | 0.085 | 2 |
| R5#59 | -7.4 | 0.181 | 0.171 | 0.085 | 2 |
| R5#60 | -7.9 | 0.181 | 0.171 | 0.085 | 2 |
| R5#61 | -4.1 | 0.182 | 0.170 | 0.084 | 1 |
| R5#62 | -8.5 | 0.180 | 0.171 | 0.085 | 2 |
| R5#63 | -6.7 | 0.182 | 0.170 | 0.083 | 1 |
| R5#64 | -2.9 | 0.181 | 0.171 | 0.083 | 2 |
| R5#65 | -2.6 | 0.180 | 0.171 | 0.083 | 1 |
| R5#66 | -5.2 | 0.182 | 0.170 | 0.083 | 2 |
| R5#67 | -7.9 | 0.181 | 0.171 | 0.085 | 2 |
| R5#68 | -5.0 | 0.179 | 0.172 | 0.084 | 2 |
| R5#69 | -7.1 | 0.182 | 0.170 | 0.082 | 2 |
| R5#70 | -4.4 | 0.179 | 0.172 | 0.084 | 1 |
| R5#71 | -7.5 | 0.178 | 0.172 | 0.085 | 1 |
| R5#72 | -6.4 | 0.182 | 0.170 | 0.084 | 1 |
| R5#73 | -2.9 | 0.181 | 0.171 | 0.083 | 2 |
| R5#74 | -6.2 | 0.174 | 0.174 | 0.083 | 2 |
| R5#75 | -6.3 | 0.182 | 0.170 | 0.084 | 1 |
| R5#76 | -3.5 | 0.182 | 0.170 | 0.084 | 1 |
| R5#77 | -6.6 | 0.182 | 0.170 | 0.082 | 2 |
| R5#78 | -4.3 | 0.181 | 0.171 | 0.085 | 1 |
| R5#79 | -9.3 | 0.179 | 0.171 | 0.083 | 2 |
| R5#80 | -5.0 | 0.182 | 0.170 | 0.083 | 1 |
| R5#81 | -7.3 | 0.185 | 0.169 | 0.082 | 2 |
| R5#82 | -7.9 | 0.178 | 0.172 | 0.085 | 2 |
| R5#83 | -3.1 | 0.182 | 0.170 | 0.084 | 1 |
| R5#84 | -6.7 | 0.181 | 0.171 | 0.085 | 1 |
| R5#85 | -5.7 | 0.182 | 0.170 | 0.084 | 1 |
| R5#86 | -2.7 | 0.182 | 0.170 | 0.084 | 1 |
| R5#87 | -2.9 | 0.181 | 0.171 | 0.083 | 2 |
| R5#88 | -4.4 | 0.179 | 0.172 | 0.084 | 1 |
| R5#89 | -2.5 | 0.182 | 0.170 | 0.084 | 1 |
| R5#90 | -4.6 | 0.182 | 0.170 | 0.083 | 2 |
| R5#91 | -5.5 | 0.182 | 0.170 | 0.084 | 2 |
| R5#92 | -7.9 | 0.181 | 0.171 | 0.084 | 2 |
| R5#93 | -2.9 | 0.181 | 0.171 | 0.083 | 2 |
| R5#94 | -9.9 | 0.182 | 0.170 | 0.083 | 1 |
| R5#95 | -4.4 | 0.181 | 0.171 | 0.084 | 1 |
| R5#96 | -4.9 | 0.182 | 0.170 | 0.082 | 2 |
| R5#97 | -6.6 | 0.181 | 0.171 | 0.085 | 1 |
| R5#98 | -5.2 | 0.182 | 0.170 | 0.083 | 2 |
| R5#99 | -8.4 | 0.179 | 0.172 | 0.085 | 2 |
| R5#100 | -7.0 | 0.182 | 0.170 | 0.084 | 2 |
| R6#1 | -7.9 | 0.179 | 0.172 | 0.084 | 2 |
| R6#2 | -4.9 | 0.182 | 0.170 | 0.082 | 2 |
| R6#3 | -5.2 | 0.182 | 0.170 | 0.083 | 2 |
| R6#4 | -11.8 | 0.182 | 0.170 | 0.083 | 2 |
| R6#5 | -2.9 | 0.181 | 0.171 | 0.083 | 2 |
| R6#6 | -8.7 | 0.183 | 0.170 | 0.082 | 2 |
| R6#7 | -6.4 | 0.176 | 0.173 | 0.085 | 2 |
| R6#8 | -9.3 | 0.176 | 0.173 | 0.083 | 2 |
| R6#9 | -7.4 | 0.179 | 0.171 | 0.083 | 2 |
| R6#10 | -4.3 | 0.179 | 0.171 | 0.084 | 2 |
| R6#11 | -5.6 | 0.177 | 0.172 | 0.083 | 2 |
| R6#12 | -4.0 | 0.182 | 0.170 | 0.084 | 2 |
| R6#13 | -7.3 | 0.185 | 0.169 | 0.082 | 2 |
| R6#14 | -7.3 | 0.185 | 0.169 | 0.082 | 2 |
| R6#15 | -9.3 | 0.179 | 0.171 | 0.083 | 2 |
| R6#16 | -5.2 | 0.177 | 0.172 | 0.082 | 2 |
| R6#17 | -8.1 | 0.180 | 0.171 | 0.084 | 2 |
| R6#18 | -7.4 | 0.179 | 0.172 | 0.084 | 2 |
| R6#19 | -5.1 | 0.181 | 0.170 | 0.083 | 2 |
| R6#20 | -9.1 | 0.180 | 0.171 | 0.082 | 2 |
| R6#21 | -7.0 | 0.182 | 0.170 | 0.084 | 2 |
| R6#22 | -4.9 | 0.182 | 0.170 | 0.082 | 2 |
| R6#23 | -6.0 | 0.182 | 0.170 | 0.082 | 1 |
| R6#24 | -7.6 | 0.183 | 0.170 | 0.082 | 2 |
| R6#25 | -6.2 | 0.182 | 0.170 | 0.083 | 1 |
| R6#26 | -7.9 | 0.179 | 0.172 | 0.084 | 2 |
| R6#27 | -4.7 | 0.180 | 0.171 | 0.085 | 1 |
| R6#28 | -7.9 | 0.179 | 0.172 | 0.084 | 2 |
| R6#29 | -7.7 | 0.179 | 0.172 | 0.084 | 1 |
| R6#30 | -7.0 | 0.181 | 0.171 | 0.083 | 2 |
| R6#31 | -5.8 | 0.182 | 0.170 | 0.083 | 2 |
| R6#32 | -8.1 | 0.177 | 0.172 | 0.083 | 2 |
| R6#33 | -6.5 | 0.181 | 0.170 | 0.082 | 1 |
| R6#34 | -8.7 | 0.179 | 0.172 | 0.084 | 2 |
| R6#35 | -7.9 | 0.179 | 0.172 | 0.084 | 2 |
| R6#36 | -5.2 | 0.177 | 0.172 | 0.082 | 2 |
| R6#37 | -7.6 | 0.179 | 0.172 | 0.084 | 2 |
| R6#38 | -2.9 | 0.181 | 0.171 | 0.083 | 2 |
| R6#39 | -4.9 | 0.182 | 0.170 | 0.082 | 2 |
| R6#40 | -1.9 | 0.182 | 0.170 | 0.084 | 1 |
| R6#41 | -4.8 | 0.184 | 0.169 | 0.084 | 1 |
| R6#42 | -4.3 | 0.173 | 0.175 | 0.088 | 2 |
| R6#43 | -5.2 | 0.182 | 0.170 | 0.083 | 2 |
| R6#44 | -7.9 | 0.179 | 0.172 | 0.084 | 2 |
| R6#45 | -4.9 | 0.181 | 0.171 | 0.083 | 2 |
| R6#46 | -7.4 | 0.179 | 0.172 | 0.084 | 2 |
| R6#47 | -4.9 | 0.182 | 0.170 | 0.082 | 2 |
| R6#48 | -4.0 | 0.182 | 0.170 | 0.084 | 2 |
| R6#49 | -4.9 | 0.182 | 0.170 | 0.082 | 2 |
| R6#50 | -4.3 | 0.179 | 0.172 | 0.084 | 2 |
| R6#51 | -7.9 | 0.179 | 0.172 | 0.084 | 2 |
| R6#52 | -9.6 | 0.180 | 0.171 | 0.085 | 2 |
| R6#53 | -5.2 | 0.181 | 0.171 | 0.083 | 2 |
| R6#54 | -9.7 | 0.181 | 0.170 | 0.084 | 2 |
| R6#55 | -7.1 | 0.182 | 0.170 | 0.082 | 2 |
| R6#56 | -6.6 | 0.182 | 0.170 | 0.082 | 2 |
| R6#57 | -6.7 | 0.179 | 0.172 | 0.084 | 2 |
| R6#58 | -5.0 | 0.179 | 0.172 | 0.084 | 2 |
| R6#59 | -7.4 | 0.179 | 0.172 | 0.084 | 2 |
| R6#60 | -6.7 | 0.182 | 0.170 | 0.083 | 1 |
| R6#61 | -6.9 | 0.183 | 0.170 | 0.082 | 2 |
| R6#62 | -9.6 | 0.182 | 0.170 | 0.083 | 2 |
| R6#63 | -5.2 | 0.182 | 0.170 | 0.083 | 2 |
| R6#64 | -7.9 | 0.179 | 0.172 | 0.084 | 2 |
| R6#65 | -10.4 | 0.182 | 0.170 | 0.083 | 1 |
| R6#66 | -8.5 | 0.180 | 0.171 | 0.085 | 2 |
| R6#67 | -5.0 | 0.182 | 0.170 | 0.082 | 1 |
| R6#68 | -9.9 | 0.182 | 0.170 | 0.083 | 1 |
| R6#69 | -7.9 | 0.181 | 0.171 | 0.084 | 2 |
| R6#70 | -4.9 | 0.182 | 0.170 | 0.082 | 2 |
| R6#71 | -5.6 | 0.183 | 0.170 | 0.082 | 2 |
| R6#72 | -9.7 | 0.177 | 0.173 | 0.086 | 2 |
| R6#73 | -2.9 | 0.181 | 0.171 | 0.083 | 2 |
| R6#74 | -10.4 | 0.182 | 0.170 | 0.083 | 1 |
| R6#75 | -5.1 | 0.183 | 0.170 | 0.082 | 2 |
| R6#76 | -4.8 | 0.179 | 0.172 | 0.085 | 1 |
| R6#77 | -5.7 | 0.180 | 0.171 | 0.085 | 2 |
| R6#78 | -7.9 | 0.181 | 0.171 | 0.084 | 2 |
| R6#79 | -8.4 | 0.179 | 0.172 | 0.085 | 2 |
| R6#80 | -7.3 | 0.183 | 0.170 | 0.082 | 2 |
| R6#81 | -5.1 | 0.181 | 0.171 | 0.085 | 2 |
| R6#82 | -7.9 | 0.179 | 0.172 | 0.084 | 2 |
| R6#83 | -8.6 | 0.179 | 0.171 | 0.084 | 1 |
| R6#84 | -5.8 | 0.183 | 0.170 | 0.082 | 2 |
| R6#85 | -4.3 | 0.179 | 0.171 | 0.084 | 2 |
| R6#86 | -3.1 | 0.182 | 0.170 | 0.082 | 1 |
| R6#87 | -5.9 | 0.182 | 0.170 | 0.082 | 1 |
| R6#88 | -10.4 | 0.182 | 0.170 | 0.083 | 1 |
| R6#89 | -8.3 | 0.177 | 0.173 | 0.083 | 1 |
| R6#90 | -7.9 | 0.178 | 0.172 | 0.085 | 2 |
| R6#91 | -9.3 | 0.176 | 0.173 | 0.083 | 2 |
| R6#92 | -7.4 | 0.179 | 0.172 | 0.084 | 2 |
| R6#93 | -3.6 | 0.180 | 0.171 | 0.084 | 1 |
| R6#94 | -5.0 | 0.182 | 0.170 | 0.083 | 1 |
| R6#95 | -4.9 | 0.182 | 0.170 | 0.082 | 2 |
| R6#96 | -1.4 | 0.177 | 0.173 | 0.084 | 2 |
| R6#97 | -8.0 | 0.182 | 0.170 | 0.082 | 1 |
| R6#98 | -4.0 | 0.183 | 0.169 | 0.083 | 2 |
| R6#99 | -8.5 | 0.180 | 0.171 | 0.085 | 2 |
| R6#100 | -5.4 | 0.184 | 0.169 | 0.083 | 1 |
| R7#1 | -4.9 | 0.182 | 0.170 | 0.082 | 2 |
| R7#2 | -7.9 | 0.179 | 0.172 | 0.084 | 2 |
| R7#3 | -8.7 | 0.183 | 0.170 | 0.082 | 2 |
| R7#4 | -11.8 | 0.182 | 0.170 | 0.083 | 2 |
| R7#5 | -5.2 | 0.182 | 0.170 | 0.083 | 2 |
| R7#6 | -2.9 | 0.181 | 0.171 | 0.083 | 2 |
| R7#7 | -9.1 | 0.180 | 0.171 | 0.082 | 2 |
| R7#8 | -7.4 | 0.179 | 0.171 | 0.083 | 2 |
| R7#9 | -9.3 | 0.176 | 0.173 | 0.083 | 2 |
| R7#10 | -7.3 | 0.185 | 0.169 | 0.082 | 2 |
| R7#11 | -7.3 | 0.185 | 0.169 | 0.082 | 2 |
| R7#12 | -6.4 | 0.176 | 0.173 | 0.085 | 2 |
| R7#13 | -7.6 | 0.183 | 0.170 | 0.082 | 2 |
| R7#14 | -5.6 | 0.177 | 0.172 | 0.083 | 2 |
| R7#15 | -4.3 | 0.173 | 0.175 | 0.088 | 2 |
| R7#16 | -5.1 | 0.181 | 0.170 | 0.083 | 2 |
| R7#17 | -4.0 | 0.182 | 0.170 | 0.084 | 2 |
| R7#18 | -9.3 | 0.179 | 0.171 | 0.083 | 2 |
| R7#19 | -1.9 | 0.182 | 0.170 | 0.084 | 1 |
| R7#20 | -9.6 | 0.180 | 0.171 | 0.085 | 2 |
| R7#21 | -7.0 | 0.182 | 0.170 | 0.084 | 2 |
| R7#22 | -4.3 | 0.179 | 0.171 | 0.084 | 2 |
| R7#23 | -6.2 | 0.182 | 0.170 | 0.083 | 1 |
| R7#24 | -9.7 | 0.181 | 0.170 | 0.084 | 2 |
| R7#25 | -6.0 | 0.182 | 0.170 | 0.082 | 1 |
| R7#26 | -5.5 | 0.183 | 0.170 | 0.082 | 2 |
| R7#27 | -2.9 | 0.181 | 0.171 | 0.083 | 2 |
| R7#28 | -8.1 | 0.181 | 0.170 | 0.082 | 2 |
| R7#29 | -4.9 | 0.182 | 0.170 | 0.082 | 2 |
| R7#30 | -8.1 | 0.183 | 0.170 | 0.082 | 2 |
| R7#31 | -7.3 | 0.183 | 0.170 | 0.082 | 2 |
| R7#32 | -7.0 | 0.181 | 0.171 | 0.083 | 2 |
| R7#33 | -7.9 | 0.179 | 0.172 | 0.084 | 2 |
| R7#34 | -5.2 | 0.182 | 0.170 | 0.083 | 2 |
| R7#35 | -5.2 | 0.181 | 0.170 | 0.082 | 2 |
| R7#36 | -7.6 | 0.179 | 0.172 | 0.084 | 2 |
| R7#37 | -8.1 | 0.183 | 0.170 | 0.082 | 2 |
| R7#38 | -7.9 | 0.179 | 0.172 | 0.084 | 2 |
| R7#39 | 8.4 | 0.179 | 0.172 | 0.085 | 1 |
| R7#40 | -5.8 | 0.182 | 0.170 | 0.083 | 2 |
| R7#41 | -7.4 | 0.181 | 0.171 | 0.085 | 2 |
| R7#42 | -4.8 | 0.184 | 0.169 | 0.084 | 1 |
| R7#43 | -1.4 | 0.177 | 0.173 | 0.084 | 2 |
| R7#44 | -5.6 | 0.183 | 0.170 | 0.082 | 2 |
| R7#45 | -4.3 | 0.179 | 0.172 | 0.084 | 2 |
| R7#46 | -7.9 | 0.179 | 0.172 | 0.084 | 2 |
| R7#47 | -4.9 | 0.182 | 0.170 | 0.082 | 2 |
| R7#48 | -7.3 | 0.183 | 0.170 | 0.082 | 2 |
| R7#49 | -6.6 | 0.182 | 0.170 | 0.082 | 2 |
| R7#50 | -4.9 | 0.182 | 0.170 | 0.082 | 2 |
| R7#51 | -7.4 | 0.181 | 0.171 | 0.085 | 2 |
| R7#52 | -6.7 | 0.179 | 0.172 | 0.084 | 2 |
| R7#53 | -8.3 | 0.177 | 0.173 | 0.083 | 1 |
| R7#54 | -7.2 | 0.185 | 0.169 | 0.082 | 2 |
| R7#55 | -6.7 | 0.182 | 0.170 | 0.083 | 1 |
| R7#56 | -7.9 | 0.179 | 0.172 | 0.084 | 2 |
| R7#57 | -6.9 | 0.183 | 0.170 | 0.082 | 2 |
| R7#58 | -9.3 | 0.176 | 0.173 | 0.083 | 2 |
| R7#59 | -7.5 | 0.178 | 0.172 | 0.085 | 1 |
| R7#60 | -7.9 | 0.179 | 0.172 | 0.084 | 2 |
| R7#61 | -5.2 | 0.182 | 0.170 | 0.083 | 2 |
| R7#62 | -5.8 | 0.183 | 0.170 | 0.082 | 2 |
| R7#63 | -4.0 | 0.182 | 0.170 | 0.084 | 2 |
| R7#64 | -4.9 | 0.181 | 0.171 | 0.083 | 2 |
| R7#65 | -2.9 | 0.181 | 0.171 | 0.083 | 2 |
| R7#66 | -4.9 | 0.182 | 0.170 | 0.082 | 2 |
| R7#67 | -10.4 | 0.182 | 0.170 | 0.083 | 1 |
| R7#68 | -7.9 | 0.181 | 0.171 | 0.086 | 2 |
| R7#69 | -7.7 | 0.181 | 0.171 | 0.085 | 1 |
| R7#70 | -5.0 | 0.179 | 0.172 | 0.084 | 2 |
| R7#71 | -7.1 | 0.182 | 0.170 | 0.082 | 2 |
| R7#72 | -10.4 | 0.182 | 0.170 | 0.083 | 1 |
| R7#73 | -8.1 | 0.183 | 0.170 | 0.082 | 2 |
| R7#74 | -9.3 | 0.176 | 0.173 | 0.083 | 2 |
| R7#75 | -8.1 | 0.183 | 0.170 | 0.082 | 2 |
| R7#76 | -6.2 | 0.181 | 0.170 | 0.082 | 2 |
| R7#77 | -8.7 | 0.179 | 0.172 | 0.084 | 2 |
| R7#78 | -8.1 | 0.181 | 0.170 | 0.082 | 2 |
| R7#79 | -9.6 | 0.182 | 0.170 | 0.083 | 2 |
| R7#80 | -8.5 | 0.181 | 0.170 | 0.082 | 2 |
| R7#81 | -7.3 | 0.185 | 0.169 | 0.082 | 2 |
| R7#82 | -5.1 | 0.183 | 0.170 | 0.082 | 2 |
| R7#83 | -9.7 | 0.177 | 0.173 | 0.086 | 2 |
| R7#84 | -3.2 | 0.178 | 0.173 | 0.084 | 1 |
| R7#85 | -3.6 | 0.180 | 0.171 | 0.084 | 1 |
| R7#86 | -2.9 | 0.181 | 0.171 | 0.083 | 2 |
| R7#87 | -4.8 | 0.182 | 0.170 | 0.085 | 1 |
| R7#88 | -10.3 | 0.176 | 0.173 | 0.086 | 2 |
| R7#89 | -5.2 | 0.181 | 0.171 | 0.083 | 2 |
| R7#90 | -9.9 | 0.182 | 0.170 | 0.083 | 1 |
| R7#91 | -5.2 | 0.177 | 0.172 | 0.082 | 2 |
| R7#92 | -3.9 | 0.180 | 0.171 | 0.085 | 1 |
| R7#93 | -10.4 | 0.182 | 0.170 | 0.083 | 1 |
| R7#94 | -6.5 | 0.181 | 0.170 | 0.082 | 1 |
| R7#95 | -4.4 | 0.181 | 0.171 | 0.084 | 1 |
| R7#96 | -8.9 | 0.183 | 0.169 | 0.082 | 2 |
| R7#97 | -8.9 | 0.181 | 0.171 | 0.084 | 2 |
| R7#98 | -9.0 | 0.176 | 0.173 | 0.083 | 2 |
| R7#99 | -5.5 | 0.182 | 0.170 | 0.084 | 2 |
| R7#100 | -4.1 | 0.183 | 0.170 | 0.082 | 1 |
| R8#1 | -7.9 | 0.179 | 0.172 | 0.084 | 2 |
| R8#2 | -9.1 | 0.180 | 0.171 | 0.082 | 2 |
| R8#3 | -4.9 | 0.182 | 0.170 | 0.082 | 2 |
| R8#4 | -11.8 | 0.182 | 0.170 | 0.083 | 2 |
| R8#5 | -5.2 | 0.182 | 0.170 | 0.083 | 2 |
| R8#6 | -7.3 | 0.185 | 0.169 | 0.082 | 2 |
| R8#7 | -8.7 | 0.183 | 0.170 | 0.082 | 2 |
| R8#8 | -7.4 | 0.179 | 0.171 | 0.083 | 2 |
| R8#9 | -7.3 | 0.185 | 0.169 | 0.082 | 2 |
| R8#10 | -2.9 | 0.181 | 0.171 | 0.083 | 2 |
| R8#11 | -5.6 | 0.177 | 0.172 | 0.083 | 2 |
| R8#12 | -9.3 | 0.176 | 0.173 | 0.083 | 2 |
| R8#13 | -4.3 | 0.173 | 0.175 | 0.088 | 2 |
| R8#14 | -5.1 | 0.181 | 0.170 | 0.083 | 2 |
| R8#15 | -9.6 | 0.180 | 0.171 | 0.085 | 2 |
| R8#16 | -5.5 | 0.183 | 0.170 | 0.082 | 2 |
| R8#17 | -7.6 | 0.183 | 0.170 | 0.082 | 2 |
| R8#18 | -9.2 | 0.181 | 0.170 | 0.084 | 1 |
| R8#19 | -4.0 | 0.182 | 0.170 | 0.084 | 2 |
| R8#20 | -9.3 | 0.179 | 0.171 | 0.083 | 2 |
| R8#21 | -4.3 | 0.179 | 0.171 | 0.084 | 2 |
| R8#22 | -1.4 | 0.177 | 0.173 | 0.084 | 2 |
| R8#23 | -1.9 | 0.182 | 0.170 | 0.084 | 1 |
| R8#24 | -8.4 | 0.179 | 0.172 | 0.085 | 2 |
| R8#25 | -6.4 | 0.176 | 0.173 | 0.085 | 2 |
| R8#26 | -8.1 | 0.180 | 0.171 | 0.084 | 2 |
| R8#27 | -7.0 | 0.182 | 0.170 | 0.084 | 2 |
| R8#28 | -8.1 | 0.183 | 0.170 | 0.082 | 2 |
| R8#29 | -6.2 | 0.182 | 0.170 | 0.083 | 1 |
| R8#30 | -7.3 | 0.183 | 0.170 | 0.082 | 2 |
| R8#31 | -5.2 | 0.177 | 0.172 | 0.082 | 2 |
| R8#32 | -8.1 | 0.183 | 0.170 | 0.082 | 2 |
| R8#33 | -2.9 | 0.181 | 0.171 | 0.083 | 2 |
| R8#34 | -7.9 | 0.179 | 0.172 | 0.084 | 2 |
| R8#35 | -7.6 | 0.179 | 0.172 | 0.084 | 2 |
| R8#36 | -6.0 | 0.182 | 0.170 | 0.082 | 1 |
| R8#37 | -5.6 | 0.183 | 0.170 | 0.082 | 2 |
| R8#38 | -7.0 | 0.181 | 0.171 | 0.083 | 2 |
| R8#39 | -4.9 | 0.182 | 0.170 | 0.082 | 2 |
| R8#40 | -5.2 | 0.182 | 0.170 | 0.083 | 2 |
| R8#41 | -4.3 | 0.179 | 0.172 | 0.084 | 2 |
| R8#42 | -4.8 | 0.184 | 0.169 | 0.084 | 1 |
| R8#43 | -8.3 | 0.177 | 0.173 | 0.083 | 1 |
| R8#44 | -7.5 | 0.178 | 0.172 | 0.085 | 1 |
| R8#45 | -7.3 | 0.183 | 0.170 | 0.082 | 2 |
| R8#46 | -5.8 | 0.183 | 0.170 | 0.082 | 2 |
| R8#47 | -7.9 | 0.179 | 0.172 | 0.084 | 2 |
| R8#48 | -7.2 | 0.185 | 0.169 | 0.082 | 2 |
| R8#49 | -8.5 | 0.180 | 0.171 | 0.085 | 2 |
| R8#50 | -5.1 | 0.183 | 0.170 | 0.082 | 2 |
| R8#51 | -7.4 | 0.179 | 0.172 | 0.084 | 2 |
| R8#52 | -7.4 | 0.179 | 0.172 | 0.084 | 2 |
| R8#53 | -5.8 | 0.182 | 0.170 | 0.083 | 2 |
| R8#54 | -7.9 | 0.181 | 0.171 | 0.086 | 2 |
| R8#55 | -6.7 | 0.179 | 0.172 | 0.084 | 2 |
| R8#56 | -7.9 | 0.179 | 0.172 | 0.084 | 2 |
| R8#57 | -7.9 | 0.179 | 0.172 | 0.084 | 2 |
| R8#58 | -8.9 | 0.183 | 0.169 | 0.082 | 2 |
| R8#59 | -6.6 | 0.182 | 0.170 | 0.082 | 2 |
| R8#60 | -3.2 | 0.178 | 0.173 | 0.084 | 1 |
| R8#61 | -4.1 | 0.183 | 0.170 | 0.082 | 1 |
| R8#62 | -4.9 | 0.178 | 0.172 | 0.085 | 2 |
| R8#63 | -4.4 | 0.181 | 0.171 | 0.084 | 1 |
| R8#64 | -5.2 | 0.177 | 0.172 | 0.082 | 2 |
| R8#65 | -9.3 | 0.176 | 0.173 | 0.083 | 2 |
| R8#66 | -7.3 | 0.185 | 0.169 | 0.082 | 2 |
| R8#67 | -5.7 | 0.180 | 0.171 | 0.085 | 2 |
| R8#68 | -9.3 | 0.176 | 0.173 | 0.083 | 2 |
| R8#69 | -4.9 | 0.181 | 0.171 | 0.083 | 2 |
| R8#70 | -7.8 | 0.183 | 0.170 | 0.082 | 2 |
| R8#71 | -6.2 | 0.181 | 0.170 | 0.082 | 2 |
| R8#72 | -2.9 | 0.179 | 0.172 | 0.085 | 1 |
| R8#73 | -2.9 | 0.181 | 0.171 | 0.083 | 2 |
| R8#74 | -6.9 | 0.183 | 0.170 | 0.082 | 2 |
| R8#75 | -5.0 | 0.179 | 0.172 | 0.084 | 2 |
| R8#76 | -5.5 | 0.182 | 0.170 | 0.084 | 2 |
| R8#77 | -3.9 | 0.182 | 0.170 | 0.084 | 1 |
| R8#78 | -7.9 | 0.179 | 0.172 | 0.084 | 2 |
| R8#79 | -5.2 | 0.182 | 0.170 | 0.083 | 2 |
| R8#80 | -7.9 | 0.177 | 0.173 | 0.086 | 2 |
| R8#81 | -4.9 | 0.182 | 0.170 | 0.082 | 2 |
| R8#82 | -5.4 | 0.177 | 0.172 | 0.083 | 1 |
| R8#83 | -6.3 | 0.184 | 0.169 | 0.083 | 2 |
| R8#84 | -4.9 | 0.182 | 0.170 | 0.082 | 2 |
| R8#85 | -7.1 | 0.182 | 0.170 | 0.082 | 2 |
| R8#86 | -8.1 | 0.183 | 0.170 | 0.082 | 2 |
| R8#87 | -7.7 | 0.179 | 0.172 | 0.084 | 1 |
| R8#88 | -6.6 | 0.183 | 0.169 | 0.082 | 2 |
| R8#89 | -3.8 | 0.183 | 0.170 | 0.082 | 1 |
| R8#90 | -9.5 | 0.180 | 0.171 | 0.082 | 2 |
| R8#91 | -5.1 | 0.183 | 0.170 | 0.082 | 2 |
| R8#92 | -9.0 | 0.176 | 0.173 | 0.083 | 2 |
| R8#93 | -8.7 | 0.179 | 0.172 | 0.084 | 2 |
| R8#94 | -4.9 | 0.182 | 0.170 | 0.082 | 2 |
| R8#95 | -9.1 | 0.180 | 0.171 | 0.082 | 2 |
| R8#96 | -10.3 | 0.180 | 0.171 | 0.082 | 2 |
| R8#97 | -4.3 | 0.179 | 0.171 | 0.084 | 2 |
| R8#98 | -9.6 | 0.182 | 0.170 | 0.083 | 2 |
| R8#99 | -4.8 | 0.179 | 0.172 | 0.085 | 1 |
| R8#100 | -3.9 | 0.182 | 0.170 | 0.084 | 1 |
| R9#1 | -9.1 | 0.180 | 0.171 | 0.082 | 2 |
| R9#2 | -4.9 | 0.182 | 0.170 | 0.082 | 2 |
| R9#3 | -7.9 | 0.179 | 0.172 | 0.084 | 2 |
| R9#4 | -7.3 | 0.185 | 0.169 | 0.082 | 2 |
| R9#5 | -8.7 | 0.183 | 0.170 | 0.082 | 2 |
| R9#6 | -7.3 | 0.185 | 0.169 | 0.082 | 2 |
| R9#7 | -4.3 | 0.173 | 0.175 | 0.088 | 2 |
| R9#8 | -5.2 | 0.182 | 0.170 | 0.083 | 2 |
| R9#9 | -7.4 | 0.179 | 0.171 | 0.083 | 2 |
| R9#10 | -11.8 | 0.182 | 0.170 | 0.083 | 2 |
| R9#11 | -9.7 | 0.181 | 0.170 | 0.084 | 2 |
| R9#12 | -2.9 | 0.181 | 0.171 | 0.083 | 2 |
| R9#13 | -8.1 | 0.183 | 0.170 | 0.082 | 2 |
| R9#14 | -9.3 | 0.176 | 0.173 | 0.083 | 2 |
| R9#15 | -5.5 | 0.183 | 0.170 | 0.082 | 2 |
| R9#16 | -5.1 | 0.181 | 0.170 | 0.083 | 2 |
| R9#17 | -9.6 | 0.180 | 0.171 | 0.085 | 2 |
| R9#18 | -4.0 | 0.182 | 0.170 | 0.084 | 2 |
| R9#19 | -1.9 | 0.182 | 0.170 | 0.084 | 1 |
| R9#20 | -9.3 | 0.179 | 0.171 | 0.083 | 2 |
| R9#21 | -7.6 | 0.183 | 0.170 | 0.082 | 2 |
| R9#22 | -1.4 | 0.177 | 0.173 | 0.084 | 2 |
| R9#23 | -8.1 | 0.183 | 0.170 | 0.082 | 2 |
| R9#24 | -7.2 | 0.185 | 0.169 | 0.082 | 2 |
| R9#25 | -6.4 | 0.176 | 0.173 | 0.085 | 2 |
| R9#26 | -7.3 | 0.185 | 0.169 | 0.082 | 2 |
| R9#27 | -7.9 | 0.179 | 0.172 | 0.084 | 2 |
| R9#28 | -4.3 | 0.179 | 0.171 | 0.084 | 2 |
| R9#29 | -4.9 | 0.178 | 0.172 | 0.085 | 2 |
| R9#30 | -5.6 | 0.177 | 0.172 | 0.083 | 2 |
| R9#31 | -4.3 | 0.179 | 0.172 | 0.084 | 2 |
| R9#32 | -8.5 | 0.180 | 0.171 | 0.085 | 2 |
| R9#33 | -6.3 | 0.184 | 0.169 | 0.083 | 2 |
| R9#34 | -8.1 | 0.180 | 0.171 | 0.084 | 2 |
| R9#35 | -5.2 | 0.182 | 0.170 | 0.083 | 2 |
| R9#36 | -4.9 | 0.182 | 0.170 | 0.082 | 2 |
| R9#37 | -7.3 | 0.183 | 0.170 | 0.082 | 2 |
| R9#38 | -8.3 | 0.177 | 0.173 | 0.083 | 1 |
| R9#39 | -8.2 | 0.177 | 0.173 | 0.084 | 2 |
| R9#40 | -6.7 | 0.179 | 0.172 | 0.084 | 2 |
| R9#41 | -2.9 | 0.181 | 0.171 | 0.083 | 2 |
| R9#42 | -6.2 | 0.181 | 0.170 | 0.082 | 2 |
| R9#43 | -8.3 | 0.183 | 0.170 | 0.082 | 2 |
| R9#44 | -7.9 | 0.179 | 0.172 | 0.084 | 2 |
| R9#45 | -2.9 | 0.179 | 0.172 | 0.085 | 1 |
| R9#46 | -6.6 | 0.182 | 0.170 | 0.082 | 2 |
| R9#47 | -5.8 | 0.182 | 0.170 | 0.083 | 2 |
| R9#48 | -9.1 | 0.180 | 0.171 | 0.082 | 2 |
| R9#49 | -7.4 | 0.179 | 0.172 | 0.084 | 2 |
| R9#50 | -8.1 | 0.183 | 0.170 | 0.082 | 2 |
| R9#51 | -7.0 | 0.182 | 0.170 | 0.084 | 2 |
| R9#52 | -7.3 | 0.185 | 0.169 | 0.082 | 2 |
| R9#53 | -6.2 | 0.182 | 0.170 | 0.083 | 1 |
| R9#54 | -6.0 | 0.182 | 0.170 | 0.082 | 1 |
| R9#55 | -7.9 | 0.179 | 0.172 | 0.084 | 2 |
| R9#56 | -9.5 | 0.180 | 0.171 | 0.082 | 2 |
| R9#57 | -7.6 | 0.179 | 0.172 | 0.084 | 2 |
| R9#58 | -4.8 | 0.184 | 0.169 | 0.084 | 1 |
| R9#59 | -7.4 | 0.179 | 0.172 | 0.084 | 2 |
| R9#60 | -11.9 | 0.181 | 0.171 | 0.084 | 2 |
| R9#61 | -5.0 | 0.179 | 0.172 | 0.084 | 2 |
| R9#62 | -5.8 | 0.183 | 0.170 | 0.082 | 2 |
| R9#63 | -4.9 | 0.182 | 0.170 | 0.082 | 2 |
| R9#64 | -5.7 | 0.180 | 0.171 | 0.085 | 2 |
| R9#65 | -5.2 | 0.182 | 0.170 | 0.083 | 2 |
| R9#66 | -3.3 | 0.182 | 0.170 | 0.083 | 2 |
| R9#67 | -8.4 | 0.179 | 0.172 | 0.085 | 2 |
| R9#68 | -7.9 | 0.180 | 0.171 | 0.085 | 2 |
| R9#69 | -7.8 | 0.179 | 0.172 | 0.084 | 2 |
| R9#70 | -5.2 | 0.177 | 0.172 | 0.082 | 2 |
| R9#71 | -5.1 | 0.179 | 0.172 | 0.084 | 2 |
| R9#72 | -7.0 | 0.181 | 0.171 | 0.083 | 2 |
| R9#73 | -8.9 | 0.181 | 0.171 | 0.084 | 2 |
| R9#74 | -7.9 | 0.179 | 0.172 | 0.084 | 2 |
| R9#75 | -9.3 | 0.176 | 0.173 | 0.083 | 2 |
| R9#76 | -4.9 | 0.182 | 0.170 | 0.082 | 2 |
| R9#77 | -7.1 | 0.182 | 0.170 | 0.082 | 2 |
| R9#78 | -7.9 | 0.181 | 0.171 | 0.086 | 2 |
| R9#79 | -7.5 | 0.178 | 0.172 | 0.085 | 1 |
| R9#80 | -6.7 | 0.182 | 0.170 | 0.083 | 1 |
| R9#81 | -10.3 | 0.180 | 0.171 | 0.082 | 2 |
| R9#82 | -7.9 | 0.179 | 0.172 | 0.084 | 2 |
| R9#83 | -8.9 | 0.183 | 0.170 | 0.082 | 2 |
| R9#84 | -7.3 | 0.185 | 0.169 | 0.082 | 2 |
| R9#85 | -4.7 | 0.176 | 0.173 | 0.086 | 1 |
| R9#86 | -4.9 | 0.182 | 0.170 | 0.082 | 2 |
| R9#87 | -7.9 | 0.179 | 0.172 | 0.084 | 2 |
| R9#88 | -7.6 | 0.185 | 0.169 | 0.082 | 2 |
| R9#89 | -5.6 | 0.183 | 0.170 | 0.082 | 2 |
| R9#90 | -2.9 | 0.181 | 0.171 | 0.083 | 2 |
| R9#91 | -8.9 | 0.183 | 0.169 | 0.082 | 2 |
| R9#92 | -6.9 | 0.185 | 0.169 | 0.082 | 2 |
| R9#93 | -9.6 | 0.182 | 0.170 | 0.083 | 2 |
| R9#94 | -3.2 | 0.182 | 0.170 | 0.084 | 1 |
| R9#95 | -3.4 | 0.177 | 0.173 | 0.086 | 1 |
| R9#96 | -9.3 | 0.180 | 0.171 | 0.082 | 2 |
| R9#97 | -4.6 | 0.182 | 0.170 | 0.083 | 2 |
| R9#98 | -7.9 | 0.181 | 0.171 | 0.084 | 2 |
| R9#99 | -8.7 | 0.179 | 0.172 | 0.084 | 2 |
| R9#100 | -9.0 | 0.176 | 0.173 | 0.083 | 2 |
| R10#1 | -9.1 | 0.180 | 0.171 | 0.082 | 2 |
| R10#2 | -7.3 | 0.185 | 0.169 | 0.082 | 2 |
| R10#3 | -4.3 | 0.173 | 0.175 | 0.088 | 2 |
| R10#4 | -4.9 | 0.182 | 0.170 | 0.082 | 2 |
| R10#5 | -7.3 | 0.185 | 0.169 | 0.082 | 2 |
| R10#6 | -7.9 | 0.179 | 0.172 | 0.084 | 2 |
| R10#7 | -8.7 | 0.183 | 0.170 | 0.082 | 2 |
| R10#8 | -5.2 | 0.182 | 0.170 | 0.083 | 2 |
| R10#9 | -7.4 | 0.179 | 0.171 | 0.083 | 2 |
| R10#10 | -11.8 | 0.182 | 0.170 | 0.083 | 2 |
| R10#11 | -8.1 | 0.183 | 0.170 | 0.082 | 2 |
| R10#12 | -9.7 | 0.181 | 0.170 | 0.084 | 2 |
| R10#13 | -7.2 | 0.185 | 0.169 | 0.082 | 2 |
| R10#14 | -8.1 | 0.183 | 0.170 | 0.082 | 2 |
| R10#15 | -7.3 | 0.183 | 0.170 | 0.082 | 2 |
| R10#16 | -5.5 | 0.183 | 0.170 | 0.082 | 2 |
| R10#17 | -9.3 | 0.176 | 0.173 | 0.083 | 2 |
| R10#18 | -4.9 | 0.178 | 0.172 | 0.085 | 2 |
| R10#19 | -9.6 | 0.180 | 0.171 | 0.085 | 2 |
| R10#20 | -8.3 | 0.183 | 0.170 | 0.082 | 2 |
| R10#21 | -7.6 | 0.183 | 0.170 | 0.082 | 2 |
| R10#22 | -2.9 | 0.181 | 0.171 | 0.083 | 2 |
| R10#23 | -7.3 | 0.183 | 0.170 | 0.082 | 2 |
| R10#24 | -5.1 | 0.181 | 0.170 | 0.083 | 2 |
| R10#25 | -1.4 | 0.177 | 0.173 | 0.084 | 2 |
| R10#26 | -4.0 | 0.182 | 0.170 | 0.084 | 2 |
| R10#27 | -6.3 | 0.184 | 0.169 | 0.083 | 2 |
| R10#28 | -1.9 | 0.182 | 0.170 | 0.084 | 1 |
| R10#29 | -8.2 | 0.177 | 0.173 | 0.084 | 2 |
| R10#30 | -6.4 | 0.176 | 0.173 | 0.085 | 2 |
| R10#31 | -9.3 | 0.179 | 0.171 | 0.083 | 2 |
| R10#32 | -3.3 | 0.182 | 0.170 | 0.083 | 2 |
| R10#33 | -7.9 | 0.179 | 0.172 | 0.084 | 2 |
| R10#34 | -4.3 | 0.179 | 0.172 | 0.084 | 2 |
| R10#35 | -8.3 | 0.180 | 0.171 | 0.083 | 2 |
| R10#36 | -7.3 | 0.185 | 0.169 | 0.082 | 2 |
| R10#37 | -5.2 | 0.182 | 0.170 | 0.083 | 2 |
| R10#38 | -9.5 | 0.180 | 0.171 | 0.082 | 2 |
| R10#39 | -7.8 | 0.179 | 0.172 | 0.084 | 2 |
| R10#40 | -9.1 | 0.180 | 0.171 | 0.082 | 2 |
| R10#41 | -4.9 | 0.182 | 0.170 | 0.082 | 2 |
| R10#42 | -5.6 | 0.181 | 0.170 | 0.082 | 2 |
| R10#43 | -8.5 | 0.181 | 0.170 | 0.082 | 2 |
| R10#44 | -8.9 | 0.183 | 0.170 | 0.082 | 2 |
| R10#45 | -8.1 | 0.183 | 0.170 | 0.082 | 2 |
| R10#46 | -7.3 | 0.185 | 0.169 | 0.082 | 2 |
| R10#47 | -6.7 | 0.179 | 0.172 | 0.084 | 2 |
| R10#48 | -4.3 | 0.179 | 0.171 | 0.084 | 2 |
| R10#49 | -6.2 | 0.181 | 0.170 | 0.082 | 2 |
| R10#50 | -11.9 | 0.181 | 0.171 | 0.084 | 2 |
| R10#51 | -6.9 | 0.185 | 0.169 | 0.082 | 2 |
| R10#52 | -8.1 | 0.181 | 0.170 | 0.082 | 2 |
| R10#53 | -7.2 | 0.185 | 0.169 | 0.082 | 2 |
| R10#54 | -2.9 | 0.181 | 0.171 | 0.083 | 2 |
| R10#55 | -6.6 | 0.182 | 0.170 | 0.082 | 2 |
| R10#56 | -7.6 | 0.185 | 0.169 | 0.082 | 2 |
| R10#57 | -7.6 | 0.185 | 0.169 | 0.082 | 2 |
| R10#58 | -7.9 | 0.179 | 0.172 | 0.084 | 2 |
| R10#59 | -5.7 | 0.180 | 0.171 | 0.085 | 2 |
| R10#60 | -10.3 | 0.180 | 0.171 | 0.082 | 2 |
| R10#61 | -7.3 | 0.185 | 0.169 | 0.082 | 2 |
| R10#62 | -2.9 | 0.179 | 0.172 | 0.085 | 1 |
| R10#63 | -6.0 | 0.182 | 0.170 | 0.082 | 1 |
| R10#64 | -7.2 | 0.185 | 0.169 | 0.082 | 2 |
| R10#65 | -7.3 | 0.183 | 0.170 | 0.082 | 2 |
| R10#66 | -7.0 | 0.182 | 0.170 | 0.084 | 2 |
| R10#67 | -7.3 | 0.185 | 0.169 | 0.082 | 2 |
| R10#68 | -4.3 | 0.173 | 0.175 | 0.088 | 2 |
| R10#69 | -5.8 | 0.182 | 0.170 | 0.083 | 2 |
| R10#70 | -7.9 | 0.179 | 0.172 | 0.084 | 2 |
| R10#71 | -7.3 | 0.185 | 0.169 | 0.082 | 2 |
| R10#72 | -5.8 | 0.183 | 0.170 | 0.082 | 2 |
| R10#73 | -7.2 | 0.185 | 0.169 | 0.082 | 2 |
| R10#74 | -7.4 | 0.181 | 0.171 | 0.085 | 2 |
| R10#75 | -9.3 | 0.177 | 0.172 | 0.083 | 2 |
| R10#76 | -11.6 | 0.179 | 0.171 | 0.082 | 2 |
| R10#77 | -7.9 | 0.181 | 0.171 | 0.086 | 2 |
| R10#78 | -4.9 | 0.182 | 0.170 | 0.082 | 2 |
| R10#79 | -6.9 | 0.185 | 0.169 | 0.082 | 2 |
| R10#80 | -5.2 | 0.182 | 0.170 | 0.083 | 2 |
| R10#81 | -4.9 | 0.182 | 0.170 | 0.082 | 2 |
| R10#82 | -4.3 | 0.173 | 0.175 | 0.088 | 2 |
| R10#83 | -9.3 | 0.176 | 0.173 | 0.083 | 2 |
| R10#84 | -2.5 | 0.185 | 0.169 | 0.082 | 2 |
| R10#85 | -8.1 | 0.177 | 0.173 | 0.083 | 2 |
| R10#86 | -10.8 | 0.176 | 0.173 | 0.083 | 2 |
| R10#87 | -8.4 | 0.179 | 0.172 | 0.085 | 2 |
| R10#88 | -7.1 | 0.182 | 0.170 | 0.082 | 2 |
| R10#89 | -4.6 | 0.182 | 0.170 | 0.083 | 2 |
| R10#90 | -9.0 | 0.180 | 0.171 | 0.082 | 2 |
| R10#91 | -7.2 | 0.185 | 0.169 | 0.082 | 2 |
| R10#92 | -8.3 | 0.180 | 0.171 | 0.084 | 2 |
| R10#93 | -5.0 | 0.179 | 0.172 | 0.084 | 2 |
| R10#94 | -8.9 | 0.181 | 0.171 | 0.084 | 2 |
| R10#95 | -4.9 | 0.182 | 0.170 | 0.082 | 2 |
| R10#96 | -7.9 | 0.179 | 0.172 | 0.084 | 2 |
| R10#97 | -6.7 | 0.182 | 0.170 | 0.083 | 1 |
| R10#98 | -7.0 | 0.181 | 0.171 | 0.083 | 2 |
| R10#99 | -7.6 | 0.179 | 0.172 | 0.084 | 2 |
| R10#100 | -5.1 | 0.179 | 0.172 | 0.084 | 2 |
